# Identification and characterization of short leader and trailer RNAs synthesized by the Ebola virus RNA polymerase

**DOI:** 10.1371/journal.ppat.1010002

**Published:** 2021-10-26

**Authors:** Simone Bach, Jana-Christin Demper, Paul Klemm, Julia Schlereth, Marcus Lechner, Andreas Schoen, Lennart Kämper, Friedemann Weber, Stephan Becker, Nadine Biedenkopf, Roland K. Hartmann

**Affiliations:** 1 Institut für Pharmazeutische Chemie, Philipps-Universität Marburg, Marburg, Germany; 2 Zentrum für Synthetische Mikrobiologie, Philipps-Universität Marburg, Marburg, Germany; 3 Institut für Virologie, Justus-Liebig-Universität Gießen, Gießen, Germany; 4 Institut für Virologie, Philipps-Universität Marburg, Marburg, Germany; Friedrich-Loeffler-Institut, GERMANY

## Abstract

Transcription of non-segmented negative sense (NNS) RNA viruses follows a stop-start mechanism and is thought to be initiated at the genome’s very 3’-end. The synthesis of short abortive leader transcripts (*leader*RNAs) has been linked to transcription initiation for some NNS viruses. Here, we identified the synthesis of abortive *leader*RNAs (as well as *trailer* RNAs) that are specifically initiated opposite to (anti)genome nt 2; *leader*RNAs are predominantly terminated in the region of nt ~ 60–80. *Leader*RNA synthesis requires hexamer phasing in the 3’-leader promoter. We determined a steady-state NP mRNA:*leader*RNA ratio of ~10 to 30-fold at 48 h after Ebola virus (EBOV) infection, and this ratio was higher (70 to 190-fold) for minigenome-transfected cells. *Leader*RNA initiation at nt 2 and the range of termination sites were not affected by structure and length variation between promoter elements 1 and 2, nor the presence or absence of VP30. Synthesis of *leader*RNA is suppressed in the presence of VP30 and termination of *leader*RNA is not mediated by cryptic gene end (GE) signals in the 3’-leader promoter. We further found different genomic 3’-end nucleotide requirements for transcription versus replication, suggesting that promoter recognition is different in the replication and transcription mode of the EBOV polymerase. We further provide evidence arguing against a potential role of EBOV *leader*RNAs as effector molecules in innate immunity. Taken together, our findings are consistent with a model according to which *leader*RNAs are abortive replicative RNAs whose synthesis is not linked to transcription initiation. Rather, replication and transcription complexes are proposed to independently initiate RNA synthesis at separate sites in the 3’-leader promoter, i.e., at the second nucleotide of the genome 3’-end and at the more internally positioned transcription start site preceding the first gene, respectively, as reported for Vesicular stomatitis virus.

## Author summary

The RNA polymerase (RdRp) of Ebola virus (EBOV) initiates RNA synthesis at the 3’-leader promoter of its encapsidated, non-segmented negative sense (NNS) RNA genome, either at the penultimate 3’-end position of the genome in the replicative mode or more internally (position 56) at the transcription start site (TSS) in its transcription mode. Here we identified the synthesis of abortive replicative RNAs that are specifically initiated opposite to genome nt 2 (termed *leader*RNAs) and predominantly terminated in the region of nt ~ 60–80 near the TSS. The functional role of abortive *leader*RNA synthesis is still enigmatic; a role in interferon induction could be excluded. Our findings indirectly link *leader*RNA termination to nucleoprotein (NP) availability for encapsidation of nascent replicative RNA or to NP removal from the template RNA. Our findings further argue against the model that *leader*RNA synthesis is a prerequisite for each transcription initiation event at the TSS. Rather, our findings are in line with the existence of distinct replicase and transcriptase complexes of RdRp that interact differently with the 3’-leader promoter and intiate RNA synthesis independently at different sites (position 2 or 56 of the genome), mechanistically similar to another NNS virus, Vesicular stomatitis virus.

## Introduction

**Fig 1:**
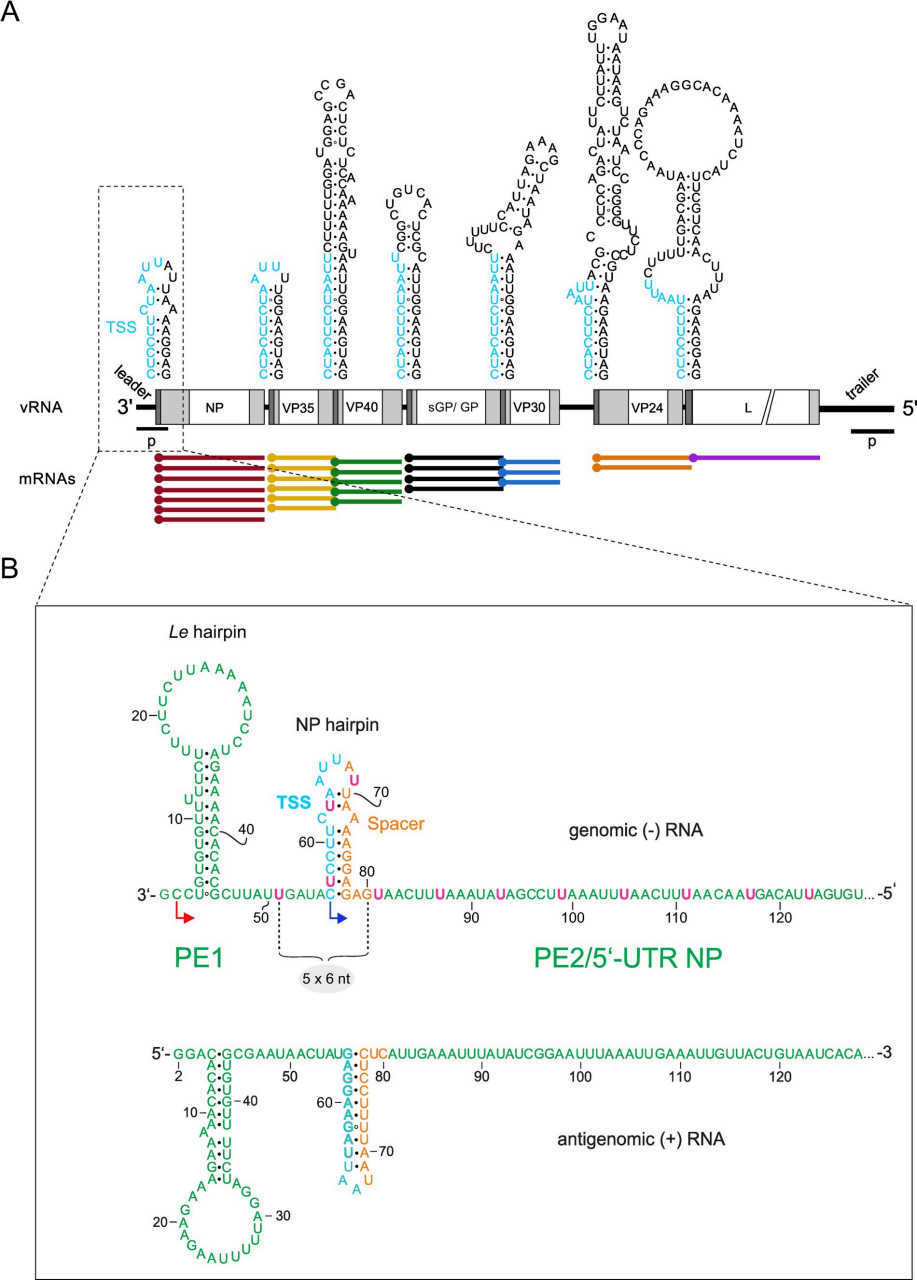
(**A**) Schematic presentation of the EBOV genome. The terminal 3’-leader and 5’-trailer regions (p, promoters) are relevant to viral RNA synthesis and encapsidation. The genome harbors 7 protein genes whose mRNA expression levels (schematically shown below the genome, terminal dots indicating the 5’-cap) decrease from the first (NP) to the last (L) mRNA. Potential secondary structures involving the 7 transcription start signals (TSS, in light blue) are depicted above the genome. White boxes indicate the coding regions for EBOV proteins (NP, VP35, VP40, GP, VP30, VP24, L) and light gray boxes 5’- and 3’-UTRs. Dark gray areas mark the position of the potential secondary structures depicted above the genome. (**B**) Close-up of the genomic 3’-leader promoter (top) and the complementary antigenomic sequence (bottom). The shown secondary structures form in the free RNAs as confirmed by structure probing [13,26,44]. Promoter elements (PE) 1 and 2 of the bipartite leader promoter are marked in green, with pink U residues denoting UN_5_ hexamers in the region between nt 51 to 128. The TSS (in cyan) and and a spacer sequence (orange residues) form the NP hairpin. Hexamer phasing in the leader promoter, manifesting as the need for a multiple of 6 nt between position 51 and 80, is crucial for EBOV replication and transcription initiation at the 3’-leader promoter [13,18,40].

Ebola virus (EBOV), a member of the *Filoviridae* family in the order *Mononegavirales*, causes a severe febrile illness with high fatality rates [1]. Its non-segmented negative sense (NNS) genome (Fig 1A), 19 kb in length, serves as template for 9 mRNAs that are translated into 7 structural proteins and two non-structural proteins. The latter two are generated by co-transcriptional editing of the glycoprotein (GP) mRNA that provides two additional versions of the GP protein, soluble (sGP) and small soluble GP (ssGP) [2,3]. The seven open reading frames are flanked by 5’- and 3’- untranslated regions (UTRs). The terminal regions of the EBOV genome encode promoters for initiation of RNA synthesis by the viral polymerase complex as well as signals important for encapsidation. The genomic 3’-leader sequence harbors the EBOV replication and transcription promoter (approx. nt 1 to 156; counted from the genome 3’-end) and the antigenomic replication promoter is encoded in the 5’-terminal 176 nt of the RNA genome [4,5,6]. The 3’-leader harbors the transcription start sequence (TSS; nt 56 to 67), and the transcription promoter is assumed to be located 3’ of the TSS but has not been pinpointed to specific nucleotide positions yet [7]. The filoviral replication promoter was shown to be bipartite, a feature that is only shared with the *Paramyxoviridae* among NNS viruses [8–11]. In the 3’-leader, the first promoter element (PE1) is located in the 3’-terminal 55 nt of the genome, preceding the TSS and a spacer sequence that together separate PE1 from the second promoter element (PE2) spanning positions 81 and 128 (Fig 1B). PE2 harbors eight consecutive 3’-UN_5_ hexamers that may encode encapsidation signals as assumed for similar sequence elements in the promoter of the paramyxovirus Nipah virus [12]. Mutational analysis of the EBOV 3’-leader promoter suggested that base identities at positions 10 to 13 as well as 44 to 55 are crucial for efficient replication [13]. Deletion of the terminal 55 or 56 nt of the trailer promoter prevented rescue of full-length EBOV, suggesting that this region is important for replication and/or encapsidation [14,15]. Likewise, removal of the terminal 25 nt of the trailer promoter reduced replication to a single round, thus abrogating *de novo* vRNA synthesis [16,17]. Furthermore, hexamer phasing between PE1 and PE2 of the 3’-leader promoter has been shown to play a key role in both, productive replication and transcription [13,18]. Transcription and replication occur at the helical viral nucleocapsid consisting of NP filaments that enwrap the template RNA [19]. In the nucleocapsid, 6 nt of the RNA are bound per NP molecule [19,20]. This makes it likely that hexamer phasing in the 3’-leader promoter is functionally linked to NP coverage.

While replication of the RNA genome is conducted by the viral polymerase L and its cofactor VP35, EBOV transcription additionally requires protein VP30 [21]. In the presence of VP30, viral replication is concurrently down-regulated [22]. VP30’s capability to enhance transcription is dependent on RNA binding, interaction with VP35 (mediated by RNA) and its phosphorylation status. While non-phosphorylated VP30 supports transcription, phosphorylation of VP30 enhances its binding affinity for NP while impairing binding to VP35, which presumably leads to release of VP30 from the viral transcription complex to favor viral replication [23–26]. Filoviral mRNA synthesis follows a stop-start mechanism regulated by highly conserved gene start (GS) and gene end (GE) sequence signals that are recognized by the viral RNA polymerase complex (RdRp) in the internal intergenic regions [27; reviewed in 28,29]. Attenuated reinitiation at downstream genes results in a progressive reduction of viral mRNA levels from the first (NP) to the last (L) gene (Fig 1A) [30,31]. Interestingly, EBOV antigenome synthesis was recently shown to be initiated not at the genome’s very 3’-end but at genome position 2. The genome’s 3’-terminal nucleotide is thought to be added to newly synthesized vRNA genomes in a non-templated manner [16].

The filoviral polymerase is thought to initiate RNA synthesis exclusively at the 3’-end of the RNA template (single-entry model). It has been shown for other NNS viruses also utilizing a sequential stop-start-transcription mechanism that the polymerase initiates RNA synthesis at the genome 3’-end, either uniformly at the 3’-terminal nucleotide (Sendai virus, SeV) or additionally at position 3 (respiratory syncytial virus, RSV). In RSV, polymerases initiating RNA synthesis 2 nt upstream of the 3’-end are thought to be unable to adopt a stable elongation mode and thus release abortive *leader*RNAs of heterogeneous length, the majority ~25 nt long; the RdRps then scan the template RNA for the next GS signal to initiate mRNA transcription [32–35]. The model further considers that replicative RNAs initiated at the 3’-terminal nucleotide may be aborted as well when the amounts of nucleoprotein are insufficient for encapsidation [36]. This raises the question as to whether EBOV shares at least some of these mechanistic features with RSV and other NNS viruses. For filoviruses, however, the production of leader transcripts has to our knowledge not been proven yet. In the present study, we were able to detect such abortive *leader*RNAs in EBOV-infected cells and EBOV minigenome (MG) systems by RNA-Seq, Northern Blot as well as qRT-PCR analyses. Further, we investigated the VP30 dependency of *leader*RNA synthesis, possible reasons for their observed length spectrum, and whether *leader*RNAs may act as effectors in innate immunity. Our findings thus provide new insights into filoviral RNA synthesis initiation.

## Material and methods

### Chemically and enzymatically synthesized RNAs

The chemically synthesized *leader*RNA mimics (65- and 73-mer) and the duplex RNA were obtained from AXOlabs (Kulmbach, Germany; see S2 Table). The 157-meric T7 transcript, encoded in plasmid pUC19_2–158 and prepared as described [26], as well as the 170- and 181-meric RNAs (S2 Table) were transcribed by T7 runoff transcription from pUC19_2–158 (157-mer) or pUC18 (170- and 181-mer) derivatives linearized with EcoR1 and purified by denaturing PAGE as described [37]. Cloning of the pUC18 derivatives was achieved by purchasing DNA oligonucleotides (Metabion, Planegg/Steinkirchen, Germany) encoding the respective RNA sequence and several extra nucleotides with terminal BamH1 and EcoRI sites, respectively. Using complementary end primers, the DNA single strands were converted to double strands by PCR, followed by cleavage with BamH1 and EcoRI and ligation into pUC18 cleaved with the same restriction enzymes.

### Cell culture

Human embryo kidney cells (HEK293; DMSZ ACC 305) and human hepatocellular carcinoma cells (HuH-7; JCRB Cell Bank, JCRB0403) were cultivated at 37°C and 5% CO_2_ in a humidified atmosphere in Dulbecco’s Modified Eagle Medium (DMEM) supplemented with 50 U/ml penicillin, 50 μg/ml streptomycin, 2 mM L-glutamine and 10% fetal calf serum (FCS). All components were purchased from Thermo Fisher Scientific (TFS). Cloning and propagation of MG plasmids was conducted in the *E*. *coli* DH5α strain using standard microbiological procedures.

### Plasmids

Plasmids used in MG assays, such as derivatives of plasmid pCAGGS encoding the Zaire EBOV nucleocapsid proteins VP30, NP, VP35 or L, as well as plasmids coding for the T7 RNA polymerase, the EBOV-specific wild-type MG (pANDY 3E5E) or a replication-deficient variant thereof, were described earlier [38,14]. For the construction of bicistronic minigenomes plasmids pA-3E5E-GLuc-GFP and pCAGGS_Luc2 were used. For details on cloning of pA-3E5E-GLuc-GFP see S1 Table. Construction of plasmid pCAGGS_Luc2 was described previously [39].

### Cloning of replication-competent (RC), replication-deficient (RD) monocistronic and RC bicistronic MG variants

MG variants were constructed as described [18,40,41], using Dpn I-based site-directed mutagenesis techniques (see S1 Fig). Cloning primers are specified in S1 Table. 3’-leader monocistronic MG variants were derived from the replication-competent (RC) EBOV minigenome pANDY 3E5E. Corresponding replication-deficient (RD) MGs were based on a pANDY 3E5E derivative lacking the terminal 55 nucleotides of the antigenome 3’-trailer replication promoter [14] using the same primers and mutagenesis setup as described for the RC MG 3’-leader variants. RC bicistronic MGs used in this study are based on a wild-type RC bicistronic (bici) MG harboring the first two EBOV cistrons, but the NP and VP35 ORFs replaced with the Renilla (RLuc) and Firefly (FFLuc/Luc2) luciferase ORF, respectively. The two ORFs, separated by the authentic NP-VP35 intergenic region (IGR), are flanked by the terminal EBOV leader and trailer regions, the NP 5’- and 3’-UTRs as well as the VP35 5’-UTR and the L 3’-UTR. Cloning of the wt bici MG and mutant variants thereof was conducted by standard restriction cloning and site-directed mutagenesis, specified and detailed in S1 Text, S1 Fig and S1 Table.

### Infection of HuH7 cells with EBOV

Work with EBOV variant Mayinga (Accession number AF086833) was conducted at the biosafety level 4 (BSL4) laboratory at the Philipps University Marburg. For EBOV RNA preparations, 8 x 10^6^ HuH7 cells in DMEM supplemented with 50 U/ml penicillin (P), 50 μg/ml streptomycin (S) and 2 mM L-glutamine (Q) (abbreviated as DMEM_P+S+Q_) were subjected to EBOV at a multiplicity of infection (MOI) of 3 for 1 h at 37°C. The inoculum was removed and cells were incubated in DMEM_P+S+Q_ additionally containing 3% FCS at 37°C and 5% CO_2_ in a humidified atmosphere. Viral RNA was extracted from EBOV-infected cells 1 day post infection.

### EBOV-specific MG assay

EBOV-specific MG assays were conducted as described earlier [18]. Briefly, 8 x 10^5^ HEK293 cells per well were seeded in 6-well plates (Greiner) and cultivated at 37°C and 5% CO_2_ in a humidified atmosphere in DMEM_P+S+Q_ supplemented with 10% FCS (3 mL/well) 18–24 h before transfection. At 60–80% cell confluency plasmids coding for the T7 promoter-driven EBOV-specific MG variants (250 ng), the EBOV nucleocapsid proteins NP (125 ng), VP35 (125 ng), VP30 (100 ng), L (1000 ng), as well as a plasmid encoding T7 RNA polymerase (250 ng) were transfected into HEK293 cells using TransIT (Mirus). In the case of monocistronic MGs, the plasmid pGL4.13 (Promega) encoding FFLuc was additionally transfected for normalization of transfection efficiencies. Cells were harvested 48 h post transfection and lysed for luciferase reporter gene assays or RNA extraction.

### Luciferase assays

Luciferase assays were performed as described [18]. For details, see the S1 Text.

### RNA-Seq: cDNA library preparation and Illumina Sequencing

Total RNA was prepared from EBOV-infected HuH7 cells or from MG-transfected HEK293 cells and either enriched for polyA+ RNA or small RNAs (< 200 nt). Preparation of cDNA libraries was performed at vertis Biotechnologie AG (Freising, Germany). For details, see S1 Text.

### Extraction and purification of total RNA for qRT-PCR analysis and Northern blotting

Total RNA of EBOV-infected HuH7 cells was isolated and purified using the mirVana miRNA Isolation Kit (with phenol; TFS) according to the manufacturer’s protocol. Total RNA of MG-transfected HEK293 cells was isolated using the RNeasy mini kit (QIAGEN) following the manufacturer’s protocol. An on-column digestion step using the RNase-Free DNase Set (QIAGEN) was included during isolation. RNA was eluted in RNase-free water. A second DNase treatment in the presence of 20 U RiboLock RNase Inhibitor (TFS) was performed by incubation with Ambion DNase I (TFS) at 37°C for 1 h. Purification was conducted using Roti-Phenol/Chloroform/Isoamyl alcohol (Carl Roth). RNA was precipitated by addition of three volumes EtOH:NaOAc [30:1; 3 M NaOAc (pH 5)]. The RNA pellet was washed with 70% EtOH, air-dried and finally redissolved in RNase-free water.

### Northern blotting

*Leader*RNA detection by Northern Blotting was performed essentially as described [42], using a digoxigenin-labeled RNA probe and immunological RNA detection with the DIG northern starter Kit (Sigma Aldrich/Merck). For more details of the protocol, see S1 Text.

### qRT-PCR

The strategies for the detection and quantification of the different viral RNAs are schematically illustrated in S2 Fig (strategies 1–4). RNAs used for the generation of qRT-PCR standard curves are summarized in S2 Table. For further experimental details, see S1 Text.

### Quantification and statistical analysis

Statistical analysis was performed using GraphPad Prism (version 8.1.1). For details (such as *p* values), see figure legends. A statistical significance level of 0.05 was chosen for analysis by the unpaired parametric Welch’s *t* test or the non-parametric Mann Whitney test.

### qRT-PCR assay for innate immune induction

Analysis of innate immune induction by qRT-PCR was done essentially as described [43]. For details, see S1 Text.

## Results

### Transcriptome analysis of EBOV-infected HuH7 cells reveals synthesis of abortive *leader*RNAs

**Fig 2:**
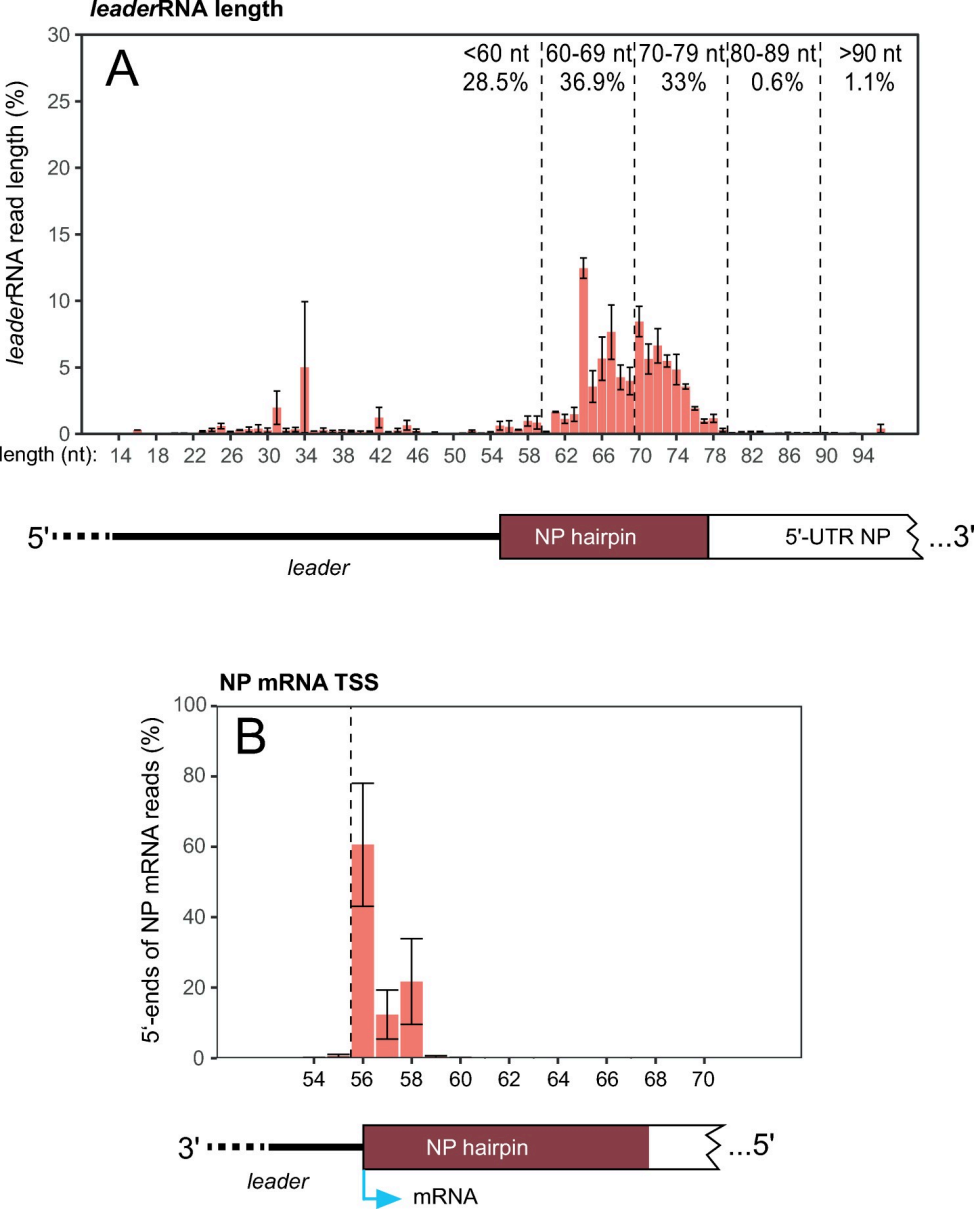
RNA-Seq analysis of (**A**) small RNAs (<200 nt) or (**B**) poly(A) RNAs derived from EBOV-infected HuH7 cells. (**A**) Length of EBOV leader transcripts in small RNA libraries. The vast majority of reads had their 5’-end at position 2, thus a 55-meric *leader*RNA had its 3’-end at the TSS (position 56); *leader*RNA reads with lengths between 15 and 100 nt were were defined as viral leader transcripts and used as read pool for *leader*RNA length/3’-end analysis. Error bars represent standard errors of the mean (SEM) calculated for each transcript length based on three biological replicates (S3 Table). Dashed vertical lines demarcate arbitrary length windows, with percentages indicated; the antigenomic leader and part of the 5’-UTR of the NP mRNA are shown schematically below the graph. (**B**) Analysis of NP mRNA reads with 5’-ends between antigenome position 54 and 70 (% of reads at each position). The dashed vertical line marks the annotated EBOV transcription start site (TSS). Error bars indicate SEM based on three biological replicates (S3 Table). The sketch at the bottom shows the genomic 3’-leader with the expected transcription start site at nt 56 (light blue arrow). The RNA-Seq analysis is consistent with position 56 being the major TSS, but left the possibility open that some transcripts may be initiated at position 57 and 58 in EBOV-infected cells.

We performed a transcriptome (RNA-Seq) analysis of EBOV-infected HuH7 cells either enriching for small RNAs <200 nt to detect potential *leader*RNAs or enriching for poly(A) RNA to detect NP mRNAs. In the small RNA libraries, considerable amounts of short transcripts antisense to the genome 3’-leader sequence were identified. The majority (~70%) was 60–80 nt in length (Fig 2A), thus resulting from termination shortly after the transcription start site (TSS) and before the PE2 region (Fig 1B). A smaller fraction of reads had a length of ~35 ± 10 nt. Hence, the RNA-Seq data provide evidence for the synthesis of abortive *leader*RNAs by the EBOV polymerase complex. The majority of termination sites coincide with the region of the NP hairpin (HP) structure. The NP HP is predicted to form on the genomic and/or antigenomic RNA level (nt 56–78, see Fig 1B) and is involved in regulation of transcription by VP30 [26,41,44,45]. In the mRNA-enriched library, most NP mRNA reads had their 5’-end at the expected position 56 and minor fractions at nt 57 and 58 (Fig 2B). This implies that the viral polymerase can also initiate transcription at positions 57/58, although at present we cannot exclude that all transcripts start with G_56_, but lost this (and the following) nucleotide during library construction or sequencing.

### Northern Blot and qRT-PCR confirm the presence of *leader*RNAs in EBOV-infected cells

**Fig 3:**
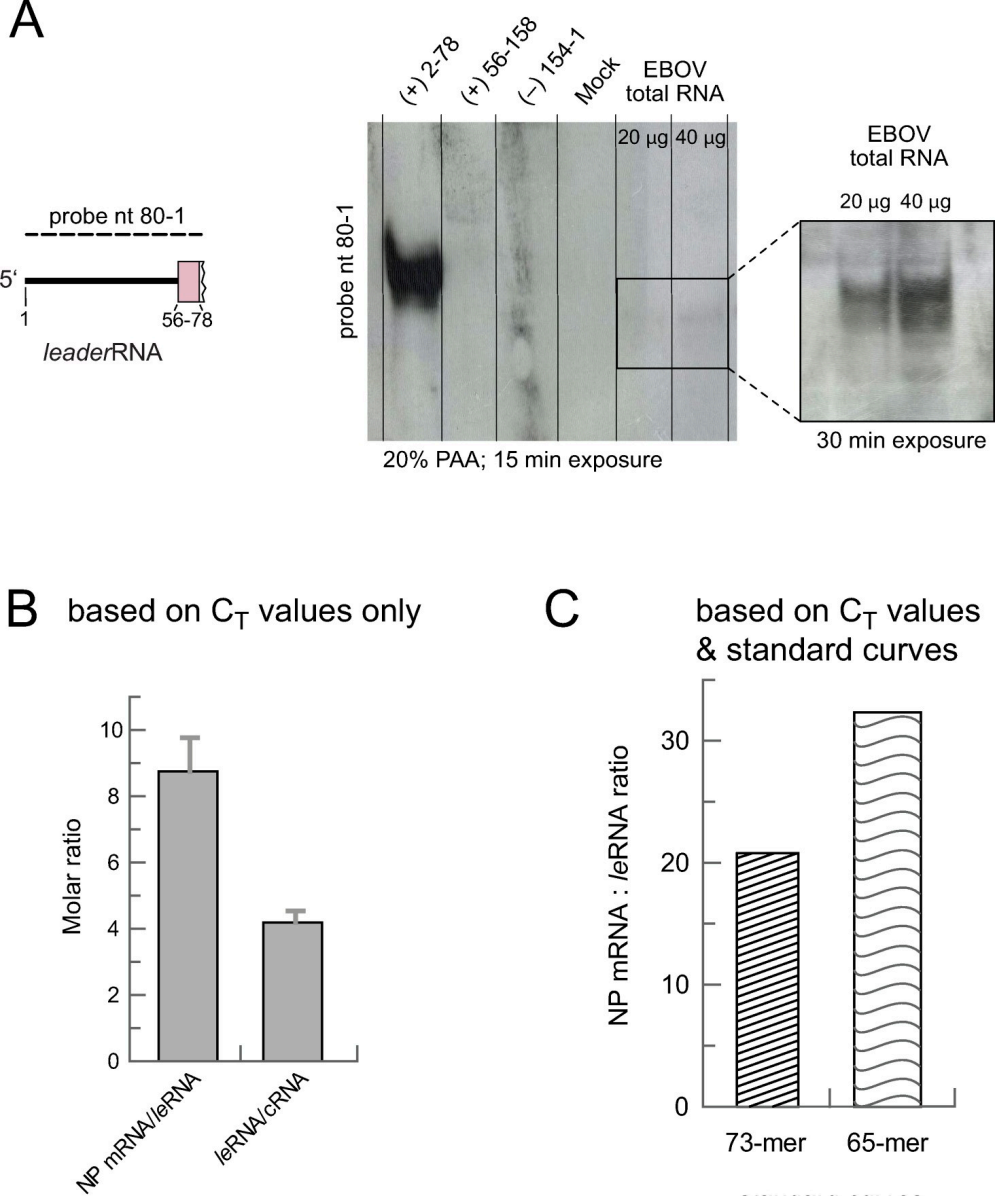
Northern Blot and qRT-PCR analysis of total RNA derived from EBOV-infected HuH7 cells. (**A**) After electrophoresis on a native 20% PAA gel, *leader*RNAs were detected by Northern blotting using a complementary, digoxigenin-labeled *in vitro*-transcribed RNA probe (nt 80–1 of the genomic RNA; schematically illustrated on the left). A T7-transcribed reference RNA (+) 2–78, corresponding to nt 2–78 of the antigenome, served as positive control and size marker; T7 transcripts (+) 56–158 and (-) 154–1 (representing the corresponding antigenome or genome sequence; see Fig 1B) were included as negative controls. Total RNA from Mock (non-infected) cells was used as additional negative control. Leader transcripts of ~ 60–70 nt were detected 15 min and 30 min post exposure. (**B**) Quantification of the NP mRNA:*leader*RNA and *leader*RNA:cRNA ratios for 3 biological replicates with 2 technical replicates each based on C_T_ values determined according to strategy 1 depicted in S2A Fig. The column graph illustrates the resulting mean ratio ± SEM for NP mRNA/*leader*RNA (8.75 ± 1.02) and *leader*RNA/cRNA (4.19 ± 0.34). An exemplary calculation for one of the replicates is shown in S3 Fig. (**C**) Molar NP mRNA/*leader*RNA ratio based on C_T_ values obtained according to RT-PCR strategy 4 (S2D Fig) and including standard curves, either based on the synthetic 73- or 65-meric *leader*RNA mimic as standard.

To validate our finding of abortive *leader*RNAs in EBOV-infected cells by RNA-Seq (Fig 2A), we performed Northern Blot analyses with a leader-specific T7-transcribed and digoxigenin-labeled probe that exhibits full complementarity to nt 1–80 of the expected leader transcript (Fig 3A, left). Indeed, we were able to detect *leader*RNAs by using 20–40 μg of total RNA extracted from EBOV-infected HuH7 cells. *In vitro*-transcribed (ivt) RNAs identical to antigenome nt 2–78 and 56–158 as well as genomic nt 154–1 (Fig 1B) were used as positive and negative controls, respectively. The major fraction of leader transcripts migrated somewhat faster than the 2–78 nt reference RNA in native 20% PAA gels. Heterogeneous transcript lengths are consistent with the RNA-Seq data (Fig 2A).

### *Leader*RNA amounts are lower than those of NP mRNA

In a third approach we used quantitative real-time PCR (qRT-PCR) to estimate the molar ratio of *leader*RNA to NP mRNA. For this purpose, we designed three primer sets for amplification of NP mRNA, *leader*RNA and longer antigenomic RNA as a measure of replicative RNA (cRNA; S2A Fig). Notably, only two of the three primer sets are specific for a single viral RNA species (NP mRNA and cRNA), whereas the leader primer set amplified *leader*RNA as well as cRNA. Using this qRT-PCR setup, we again confirmed leader transcript synthesis since the primer set detecting both, *leader*RNA and cRNA, resulted in lower C_T_ values of ~16 than the cRNA-specific primer set (C_T_ ~18) (exemplary experiment shown in S3B Fig). C_T_ values obtained with the NP mRNA-specific primer pair were clearly lower than those obtained with the *leader*RNA+cRNA primer pair (C_T_ ~13 versus C_T_ ~16), indicating that NP mRNAs are more abundant than *leader*RNAs. As the *leader*RNA primer set simultaneously detected *leader*RNA and cRNA, we indirectly calculated the ratio of NP mRNA:*leader*RNA (see S3 Fig for details). A total of three biological replicates were analyzed in technical duplicates to determine an average ratio of ~9:1 for NP mRNA:*leader*RNA amounts and ~4:1 for *leader*RNA:cRNA amounts (Fig 3B). To consider possible differences in RT primer efficiencies, we also determined the relation of RNA copy number and C_T_ values by use of enzymatically or chemically synthesized RNAs (see S2 Table for sequences) that are sequence-identical to the amplified portions of the different viral RNA species. The used primer sets are illustrated in S2D Fig (strategy 4; for primer sequences, see S1 Text, paragraph qRT-PCR, Strategy 4) and the derived standard curves are shown in S4A Fig (see also S4B Fig for specificity of the primer pairs). In this setup, the molar NP mRNA:*leader*RNA ratio increased to ~21 to 32-fold (see example calculations in S4C Fig), depending on the *leader*RNA mimic (73- or 65-mer) used for generating the standard curve (Fig 3C). The *leader*RNA levels may be somewhat underestimated, as the RT primer for *leader*RNA (covering nt 64–39), may anneal inefficiently or not at all to *leader*RNAs <60 nt. Based on *leader*RNAs <60 nt contributing ≤30% to all *leader*RNAs (Fig 2A), the mRNA:*leader*RNA ratio could maximally reduce by a factor of ~1.5. This would still correspond to a more than 10-fold molar excess of mRNA over *leader*RNA.

### *Leader*RNAs are also produced in MG systems

**Fig 4:**
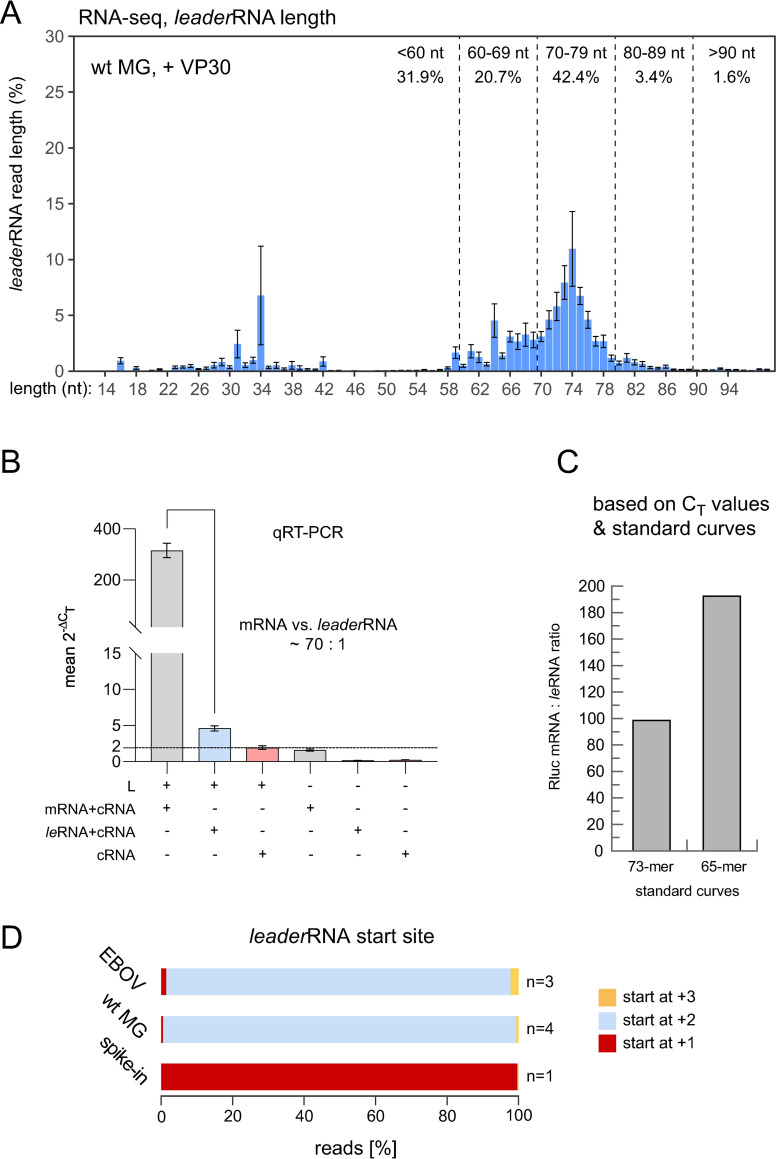
**(A)** RNA-Seq analysis of *leader*RNA length/3’ termination sites in small RNA (<200 nt) libraries derived from cells transfected with the wt MG and coexpressing VP30. The shown mean values (± SEM) are based on 3 to 5 biological replicates each. For more details, see legend to Fig 2A and S3 Table. (**B**) Quantification of viral mRNA to *leader*RNA ratio in MG-transfected HEK293 cells by a two-step qRT-PCR. The qRT-PCR setup is schematically depicted in S2B Fig (strategy 2). Relative levels (2^-ΔC_T_^ values; normalized to Firefly luciferase mRNA) of mRNA+cRNA, *le*RNA+cRNA, and cRNA in the presence (+) or absence (-) of the viral polymerase L are shown (mean 2^-ΔC_T_^ = ~314 for mRNA+cRNA; mean 2^-ΔC_T_^ = ~4.6 for *leader*RNA+cRNA, and mean 2^-ΔC_T_^ = ~2 for cRNA alone). The horizontal line marks the cRNA level. Mean values (± SEM) were derived from 5 independent experiments (exp.) with 2 (1 of 5 exp.) or 3 (4 of 5 exp.) technical replicates each. (**C**) Molar Rluc mRNA/*leader*RNA ratio based on C_T_ values obtained according to RT-PCR strategy 4 (S2D Fig) and including standard curves, either based on the synthetic 73- or 65-meric *leader*RNA mimic as standard. (**D**) *Leader*RNA 5’-ends (start sites) in RNA-Seq libraries derived from EBOV-infected HuH7 cells (upper bar), wt MG-transfected HEK293 cells (central bar), or a mock (non-infected/non-transfected) HEK293 control spiked with a synthetic 65-meric *leader*RNA (lower bar; see S2 Table for sequence). The color code is specified on the right. The number of biological replicates (n) is indicated on the right (for details, see S3 Table).

We also performed RNA-Seq using small RNA (<200 nt) preparations derived from cells transfected with monocistronic EBOV MGs. This revealed the presence of abortive *leader*RNAs as well (Fig 4A), the majority (~63%) also 60–80 nt in length owing to termination in the region between PE1 and PE2 (see Fig 2A). This demonstrated that the synthesis of abortive *leader*RNAs is not restricted to virus infections but can be investigated using EBOV-specific MG systems as well, thereby enabling mechanistic and biochemical studies outside BSL-4 laboratories. We initially quantified leader transcripts by qRT-PCR using the approach specified in S2B Fig. qRT-PCR of Firefly luciferase mRNA, synthesized from a cotransfected plasmid, served as internal standard to cancel out fluctuations in plasmid transfection efficiency [18]. As both primer sets used for Rluc mRNA or *leader*RNA amplification (S2B Fig) also amplified cRNA and primer efficiencies were comparable (both had E values of ~2.00), we could directly calculate an mRNA:*leader*RNA ratio of ~70:1 by dividing the 2^-ΔC_T_^ values for mRNA+cRNA and *leader*RNA+cRNA. Hence, in the MG context, mRNA levels exceeded those of *leader*RNA to a larger extent than in the EBOV infection setup (Figs 3B vs. 4B). As for the EBOV infection model, we also validated this finding on the basis of qRT-PCR standard curves (S2D and S4A Figs). A ~100 to 190-fold molar excess of Rluc mRNA was derived from this approach, depending on the *leader*RNA mimic (73- or 65-mer) used for generating the standard curve (Fig 4C). Again, the ratio reduces by a factor of ≤1.5 due to inefficient reverse transcription of *leader*RNA reads <60 nt (see above).

### Viral *leader*RNA synthesis is specifically initiated opposite of genome nt 2

A recent study reported that antigenome synthesis is not initiated opposite to the 3’-terminal G, but opposite to C_2_ [16]. This raised the question whether the viral polymerase initiates *leader*RNA opposite to C_2_ as well or at another site as shown for RSV (see Introduction). The vast majority of *leader*RNAs in our RNA-Seq libraries from EBOV-infected and MG-transfected cells also lacked the nucleotide complementary to genome position 1 (Fig 4D, upper and central bar). To ascertain that the absence of the first nucleotide is not an artefact of the RNA-Seq procedure or data processing, we performed an RNA-Seq experiment under identical conditions using a small RNA (<200 nt) preparation from HEK293 cells spiked with a synthetic 65-meric *leader*RNA mimic (carrying a 5’-triphosphate; for sequence information see S2 Table) that included nt C_1_. Sequencing revealed that almost 100% of the *spike-in* RNA reads included the 5’-terminal C_1_ nucleotide (Fig 4D, bottom bar), while it was predominantly lacking in RNA samples derived from either EBOV-infected or MG-transfected cells (top and central bar). This indicates that not only viral antigenomes (cRNAs) but also *leader*RNAs are intiated at genome position 2.

### First genome nucleotide is dispensable for replication but preferred in transcription

**Fig 5:**
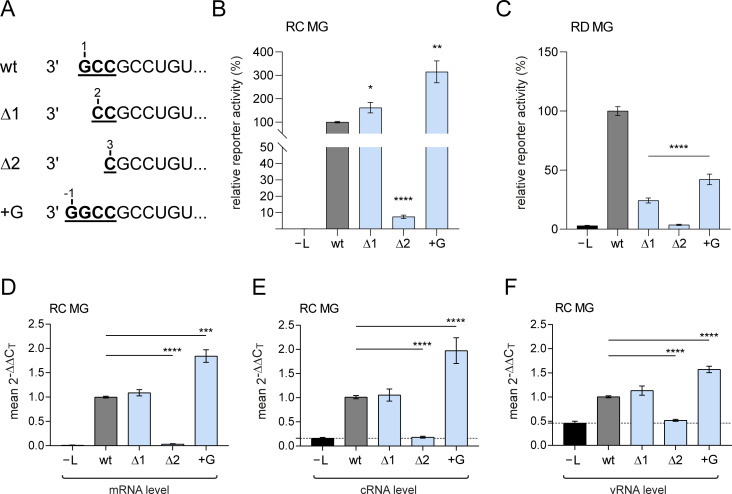
Analysis of replication-competent (RC) and replication-deficient (RD) MGs with mutated genome 3’-ends. (**A**) Genome 3’-end of the wt MG and three mutant derivatives that either lack the 3’-terminal G residue (Δ1), the 3’-terminal two residues (Δ2) or carry an extra 3’-G residue (+G). (**B**-**C**) Corresponding reporter gene assays of lysates from cells transfected with MG variants illustrated in panel A, either as part of the (**B**) RC MG or (**C**) RD MG backbone. Mean activity values (± SEM) of the native 3’-leader MG (dark gray bars) and mutant MGs (light blue bars) were derived from 3 independent experiments with 3 technical replicates each; data for the wt MG were set to 100%. As negative control, the plasmid encoding the L gene was omitted during transfection (–L; black bars). (**D**-**F**) Corresponding two-step qRT-PCR of RC MG samples using the same cells as in panel B (for qRT-PCR setup, see S2C Fig); color code as in panel B and C. Mean 2^-ΔΔCT^ values (± SEM) of viral mRNA (**D**), cRNA (**E**) and vRNA (**F**) derived from 3 independent experiments with 3 technical replicates each. *p < 0.05; **p < 0.01; ***p < 0.001; ****p < 0.0001 (unpaired Welch’s *t* test).

As the results by Deflubé et al. [16] and our data (Fig 4D) indicated genome position 2 as the start site for RNA synthesis by the viral polymerase, we addressed the question whether the genome’s first nucleotide is not only dispensable for replication but for mRNA transcription as well, considering that *leader*RNAs might be obligatory pre-products of viral NP mRNA synthesis [32]. We therefore constructed MGs lacking the 3’-terminal first (G_1_; variant Δ1) or the first two (G_1_ and C_2_; variant Δ2) nucleotides, as well as a variant carrying an additional 3’-nucleotide (variant +G, Fig 5A). We first measured reporter activity of these variants in the context of a replication-competent MG (RC MG) backbone. The Δ1 variant was even slightly more efficient than the wt construct (Fig 5B). Deletion of nucleotides 1 and 2 (variant Δ2), however, strongly decreased reporter activity. Remarkably, an additional G residue at the genome 3’-end (variant +G) substantially increased reporter activity relative to the wt MG (Fig 5B). We then analyzed the same genome variants as part of the replication-deficient MG (RD MG) backbone. In the RD MG, the viral polymerase can utilize the vRNA genomes synthesized by T7 RNAP for mRNA transcription and production of replicative cRNA, but is unable to synthesize new vRNA genomes to boost viral transcription and replication. Strikingly, all three mutant variants showed a clear defect in the RD MG setup (Fig 5C), indicating that the wt 3’-end is optimal for viral transcription; even the +G variant, most active in the RC MG system, showed decreased transcriptional activity in the RD MG context. Variant Δ2 was essentially reduced to background (–L) levels, supporting the key role of C_2_ for initiation of EBOV transcription and replication [16]. The levels of mRNA, cRNA and vRNA, derived from the same RC MG-transfected cells as used for reporter gene assays, showed the same ranking of the tested variants as observed in the reporter assay (cf. Fig 5D–5F and 5B).

### Abortive *leader*RNA synthesis does not result from alternative GE signals

**Fig 6:**
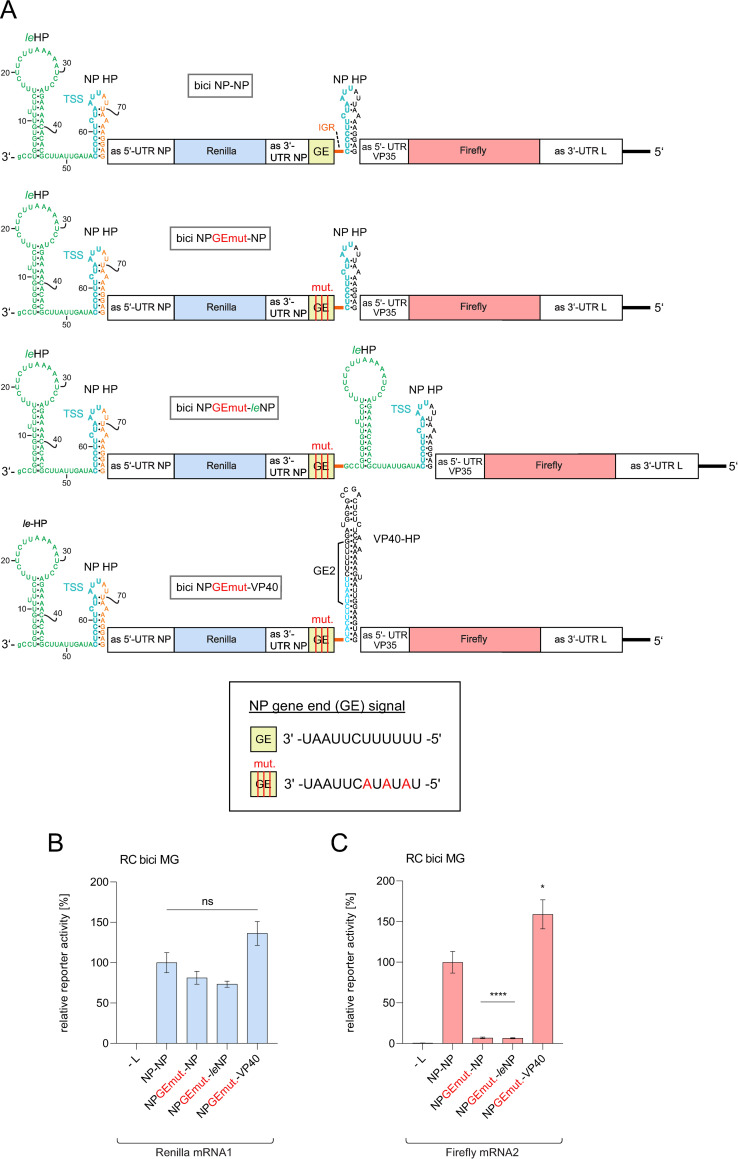
Testing for alternative gene end (GE) signals in the genomic 3’-leader promoter using a bicistronic (bici) MG system reflecting the EBOV NP-VP35 gene border. (**A**) Bici MGs encode a Renilla (light blue box) and Firefly (light red box) luciferase (for further details on color coding, see Fig 1). In construct bici NP-NP, the native VP35 5’-UTR hairpin at the second cistron was exchanged with the NP 5’-UTR hairpin. Then, the MG variant bici NPGEmut-NP was constructed, in which the native NP GE sequence (GEmut, light green box) was mutated (marked by red vertical lines, mutations shown in the box at the bottom). For this construct, one would expect Firefly luciferase expression only if the NP HP harbored an alternative GE signal. In the third test construct (bici NPGEmut-*le*NP), a derivative of bici NPGEmut-NP, the entire 3’-terminal 78 nt of the 3’-leader promoter were inserted into the intergenic region. In construct bici NPGEmut-VP40, the NP HP at the second cistron was replaced with the VP40 5’-UTR HP that introduces a functional second GE signal (GE2) to replace the inactivated GE signal at the end of the NP gene; this variant served as positive control. (**B**-**C**) Corresponding Renilla (**B**) and Firefly (**C**) luciferase reporter gene assays were used as an indirect readout for viral transcription of mRNA 1 encoding Renilla and mRNA 2 encoding Firefly luciferase. Activity values of the wt NP-NP MG were set to 100%.–L, negative control in which the plasmid encoding L was omitted during transfection. Mean values ± SEM were derived from 3 independent experiments with at least 2 technical replicates each. *p < 0.05; ****p < 0.0001; n.s., not significant (unpaired Welch’s *t* test).

The RNA-Seq results indicated that leader transcripts are predominantly terminated immediately after the polymerase has passed the NP GS signal in EBOV-infected (Fig 2A) or MG-transfected cells (Fig 4A). For RSV, it was noticed that leader promoter nucleotides essential for both transcription and replication are also conserved at the corresponding positions in the GS of the L gene [33]. This suggested the possibility that *leader*RNAs may be terminated by an unidentified termination (GE) signal, thus also employing a mechanism similar to the stop-start mechanism ubiquitously used by NNS viruses for mRNA transcription at internal genes. To address the possible presence of an alternative GE signal that could explain the production of abortive *leader*RNAs in EBOV, we constructed a bicistronic MG mimicking the first two EBOV genes, which had the following features (Fig 6A, top): it comprised the authentic 3’-leader and 5’-trailer sequences, the native NP-VP35 gene border, but the protein-coding regions of the NP and VP35 genes replaced with two different luciferase genes (corresponding to mRNA 1 and mRNA 2); in addition, the L 3’-UTR substituted for the VP35 3’-UTR, and the VP35 5’-UTR hairpin was replaced with the NP 5’-UTR hairpin (construct termed “bici NP-NP”). To find out if the NP hairpin itself or if *cis*-acting sequences within the NP hairpin structure are sufficient for alternative transcript termination, we first inactivated the NP GE signal at the NP-VP35 gene border by mutation (Fig 6A, bici NPGEmut-NP, second from top). In the third construct, a derivative of the second, we further inserted the 3’-terminal 55 leader nt (PE1) to investigate if either sequences between leader end and NP hairpin are recognized as alternative GE signals or if nucleotides in PE1 and the NP hairpin may somehow interact to induce transcript termination (Fig 6A, bici NPGEmut-*le*NP). Note that we expected synthesis of mRNA 2 and thus Firefly luciferase (FF) reporter gene activity only if an alternative GE signal compensated the inactivated NP GE signal at the NP-VP35 gene border. As positive control we included an additional derivative construct in which we replaced the NP hairpin at the 2^nd^ cistron with the VP40 5’-UTR hairpin (see Figs 1A and 6A, bottom). The VP40 HP harbors an overlapping GS-GE signal, thus in our bicistronic MG context it introduces a functional GE signal that enables termination of mRNA 1 despite the defective GE signal at the NP-VP35 gene border (Fig 6A, bici NPGEmut-VP40). In luciferase reporter assays, we did not observe substantial FF reporter activity for MG constructs 2 (NPGEmut-NP) and 3 (NPGEmut-*le*NP) that carried the mutant NP GE signal, while our positive control resulted in FF reporter activity comparable and even higher than that of the bici NP-NP MG construct (Fig 6B and 6C). Hence, it is unlikely that an alternative GE signal within the 3’-terminal 78 nt of the leader mediates transcript termination. Residual reporter gene levels above the background (–L) control in the case of constructs 2 and 3 might be explained by residual induction of termination by the mutated GE signal. Alternatively, this residual firefly luciferase activity could originate from translation of readthrough mRNAs (cotranscripts of mRNA 1 and 2).

### VP30 suppresses leader transcript synthesis

**Fig 7:**
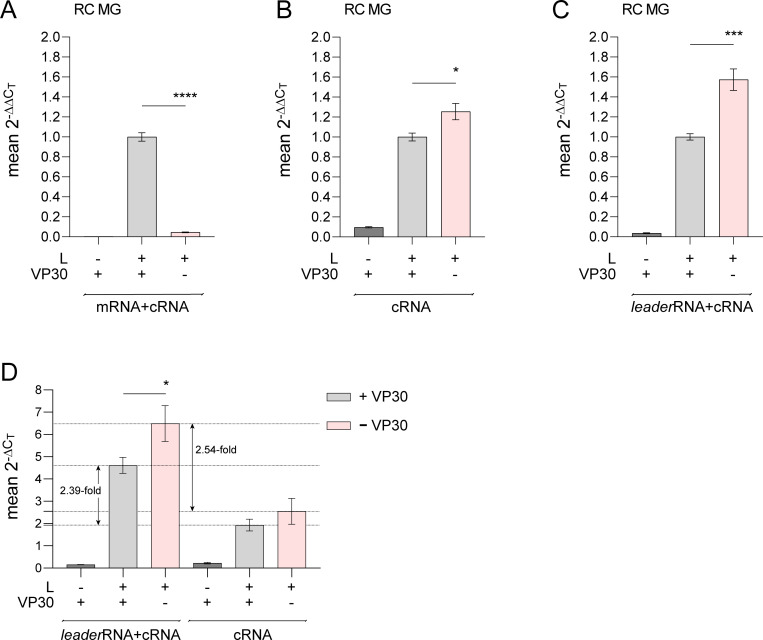
*leader*RNA synthesis in the presence or absence of VP30. (**A**-**C**) Two-step strand-specific qRT-PCR (see S2B Fig) quantification of (**A**) mRNA+cRNA, (**B**) cRNA or (**C**) *leader*RNA+cRNA using the 2^-ΔΔC_T_^ method as described [18]. HEK293 cells were transfected with the RC MG encoding the native 3’-leader, either with or without coexpression of VP30. Transfections without the plasmid encoding L served as negative controls. Mean 2^-ΔΔC_T_^ values (± SEM) were derived from 5 independent experiments (exp.) with 3 (4 of 5 exp.) or 2 (1 of 5 exp.) technical replicates. (**D**) Mean 2^-ΔC_T_^ values (± SEM) of *leader*RNA+cRNA versus cRNA, corresponding to samples analyzed in panel C and B, respectively. 2^-ΔC_T_^ values were determined according to strategy 2 (S1 Text, S2B Fig). *p < 0.05; ***p < 0.001; ****p < 0.0001 (unpaired Welch’s *t* test).

The NP hairpin structure regulates VP30-dependent transcription initiation at the TSS [41,44]. To examine if VP30 affects *leader*RNA synthesis, we applied the qRT-PCR setup illustrated in S2B Fig. As expected, mRNA+cRNA levels, predominantly representing mRNA levels (Fig 4B), largely decreased in the absence of VP30, while cRNA alone was significantly increased (Fig 7A and 7B). This is in line with previous qRT-PCR data demonstrating that a decrease of transcription correlates with an increase of replication [23,25,41]. In addition, *leader*RNA+cRNA levels were also increased in the absence of VP30 (Fig 7C). This increase was even more pronounced (~1.6-fold; Fig 7C) compared to the increase observed for cRNA alone (~1.2-fold; Fig 7B), emphasizing that *leader*RNA levels increase as well in the absence of VP30. A direct comparison of 2^-ΔC_T_^ values obtained by the *leader*RNA+cRNA and the cRNA-specific primer sets illustrates the overall relations (Fig 7D): in the presence of VP30 (gray columns), *leader*RNA+cRNA levels are 2.39 fold higher than the cRNA level; this ratio increases to 2.54 fold based on the mean 2^-ΔC_T_^ values obtained in the absence of VP30 (pink columns), in line with enhanced *leader*RNA and cRNA synthesis in the absence of VP30. As mentioned before, the measured *leader*RNA levels are lower estimates due to inefficient RT-PCR amplification of *leader*RNAs <60 nt.

**Fig 8:**
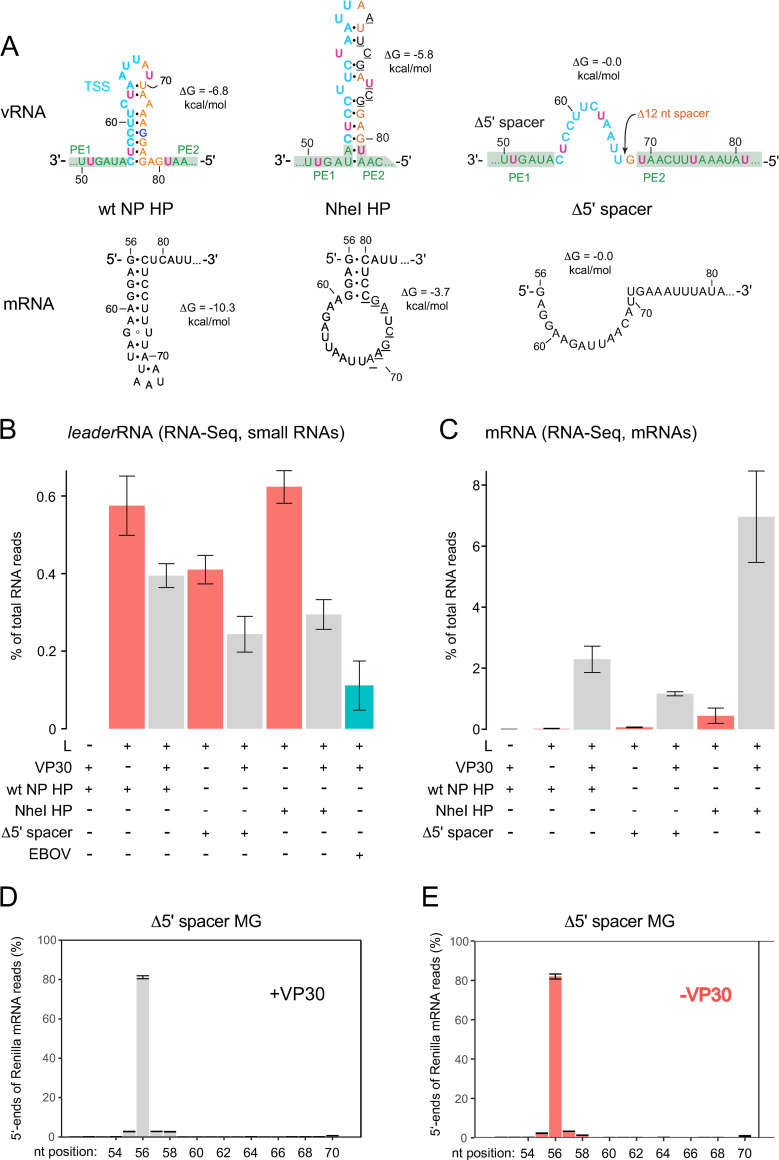
RNA-Seq analysis of relative leader transcript and Renilla mRNA levels in RC MG systems, with (+) and without (-) cotransfection of the plasmid encoding VP30. (**A**) Illustration of the MG encoding the wt NP HP and mutant derivatives either encoding the NheI HP [44] destabilized particularly on the mRNA level or the Δ5’ spacer variant [40] devoid of any hairpin structure on the genomic and mRNA level. Minimum free energies (MFE; ΔG) of the secondary structures were predicted by RNAfold using the default parameters [80]. Black underlined residues in the NheI HP mark mutated residues. For more contextual details, see Fig 1B. (**B**) Fraction of *leader*RNA reads (in %) in small RNA (< 200 nt) libraries derived from cells transfected with the MGs illustrated in panel A; the corresponding fraction (0.11 ± 0.06) is also shown for EBOV-infected cells. For the definition of l*eader*RNAs, see legend to Fig 2A; error bars are standard errors. The difference between the relative read numbers of *leader*RNAs with and without VP30 cotransfection is significant if all MGs are pooled together (***p = 0.00025, Welch’s *t* test). The individual difference for the NheI NP construct is significant as well (**p = 0.0022, Welch’s *t* test) but not for the other MGs (p = 0.13 for wt NP, p = 0.054 for Δ5’ spacer, Welch’s *t* test). (**C**) Fraction of Rluc mRNA reads (in %) in corresponding poly(A) RNA-enriched libraries. For details (panel B and C) on sample and library preparation, Illumina Sequencing, sequencing analyses and biological replicates, see the S1 Text and S3 Table. (**D**, **E**) Analysis of Rluc mRNA reads with 5’-ends between antigenome position 54 and 70 (% of reads at each position) in poly(A) RNA-enriched libraries derived from cells transfected with the Δ5’ spacer MG in (**D**) the presence (+) or (**E**) absence (-) of VP30. Percent values were normalized to the sum of all mRNA reads with 5’-ends mapping to positions 54 to 70, based on two biological replicates (± SEM) in panel D and three biological replicates (± SEM) in panel E (see S3 Table for details). The data indicate position 56 as the major transcription initiation site.

RNA-Seq, as introduced in Fig 2, was performed with RNA from cells transfected with the wt NP MG, with a MG construct harboring a destabilized hairpin (NheI NP) and with the MG variant Δ5’ spacer lacking the capacity to form a hairpin structure owing to a 12-nt deletion in the spacer between PE1 and PE2 (Fig 8A) [40,44]. In line with the qRT-PCR results, we observed an increase in *leader*RNA amounts in the absence of VP30 for the wt (NP) MG and the two mutant MGs (Fig 8B, red columns), while mRNA levels largely decreased in the absence of VP30 (Fig 8C). The findings show that this reverse regulation of *leader*RNA and mRNA 1 levels by VP30 is not dependent on the presence of the native NP hairpin in the 3’-leader.

### The position of transcription initiation is neither affected by deviations from the native NP hairpin structure nor by the presence or absence of VP30

We wondered if deviations from the native NP hairpin structure and presence versus absence of VP30 might affect selection of the transcription initiation site. However, RNA-Seq revealed that, even in the absence of VP30, mRNA synthesis was predominantly initiated opposite to genome nt -56 in case of the Δ5’ spacer MG (Fig 8D and 8E), the NheI NP MG (S5 Fig) and the wt (NP) MG (S5 Fig). Thus, recognition of the TSS and site-specific initiation of mRNA synthesis is basically the same in the presence or absence of VP30, although the overall efficiency of viral transcription is very low in the absence of VP30 [41].

### *Leader*RNA termination is independent of RNA structure formation potential at the TSS

**Fig 9:**
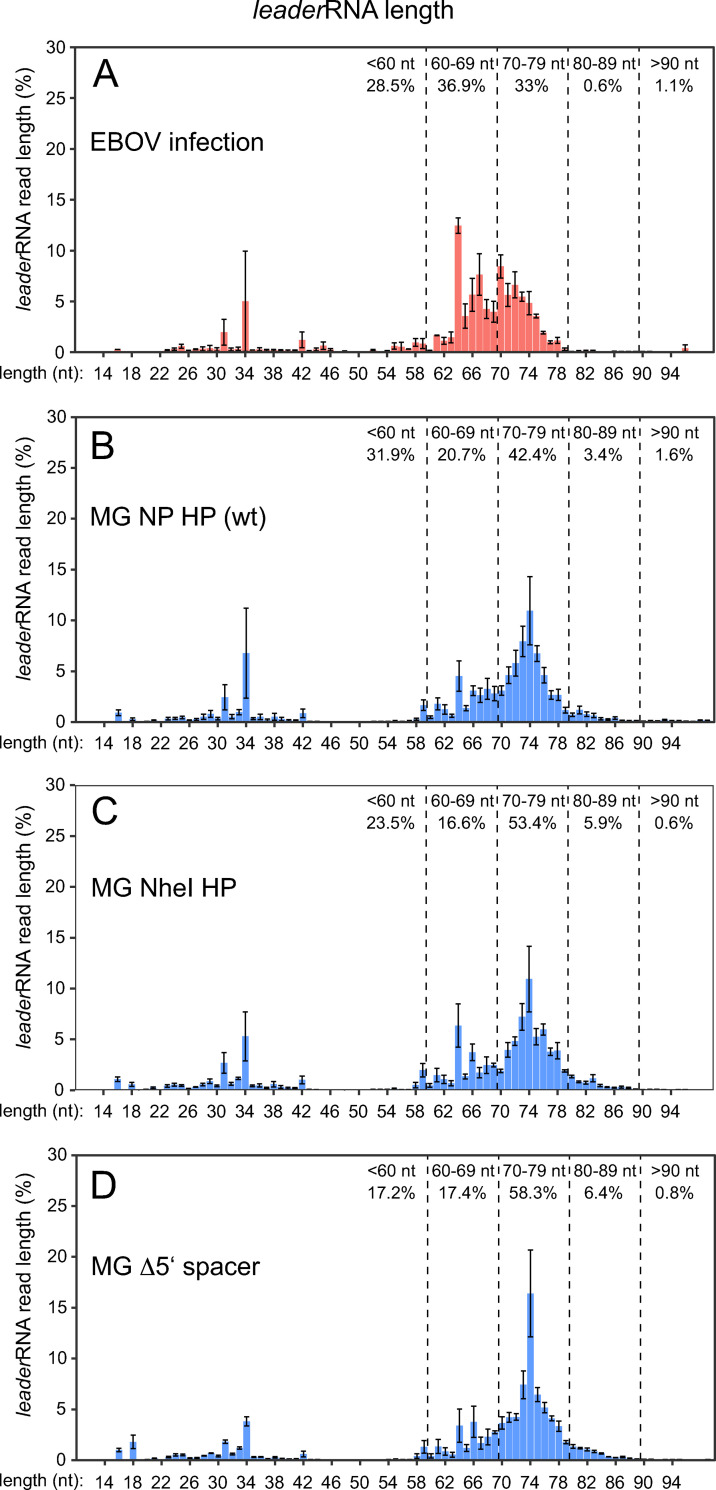
Comparative RNA-Seq analysis of *leader*RNA lengths in (**A**) EBOV-infected HuH7 cells or HEK293 cells transfected with (**B**) MG NP HP (wt), (**C**) MG NheI HP or (**D**) MG Δ5’ spacer. Panel A is identical to Fig 2A and included for comparison; the mean vales (± SEM) in panels B to D are based on four (each wt MG and MG NheI) or five (MG Δ5’ spacer) biological replicates. For more details, see legend to Fig 2A and S3 Table.

Overall, 3’-ends of *leader*RNAs, as inferred from RNA-Seq, map to the same region for EBOV-infected cells as well as cells transfected with the wt (NP) MG and the two aforementioned mutant constructs (Fig 9A–9D; for more details, see Discussion). Of note, reads terminating in the nt 70 region contain an increased fraction of non-templated nucleotides, which seems even more pronounced in the MG-transfected versus EBOV-infected system (S6A–S6D Fig). As the *leader*RNA length profiles for wt (NP) MG and the Δ5’ spacer MG devoid of any secondary structure are very similar, we can conclude that termination of leader transcripts is not influenced by the potential to form RNA structures in the spacer between PE1 and PE2.

### Initiation and termination sites for *leader* transcripts are independent of VP30

We then comparatively analyzed the small RNA libraries for *leader*RNA initiation and termination sites in RNA samples derived from cells transfected with the wt (NP) MG versus the NheI NP and Δ5 ‘spacer MGs, either in the presence or absence of VP30. For all three MG variants and independent of VP30, *leader*RNA 5’-ends mapped to position 2 (S7A Fig). Only ~0.2% of reads mapped to position 1, similar to *leader*RNAs isolated from EBOV-infected cells (Fig 4D). In all cases, the RNA-Seq data provided no evidence for substantial changes in the respective 3’-end patterns of *leader*RNAs upon omission of VP30 (cf. S6E–S6G and S6B–S6D Fig). We conclude that VP30 neither affects the position of *leader*RNA initiation nor the pattern of termination sites.

### Leader transcripts are not synthesized if hexamer spacing between PE1 and PE2 is violated

**Fig 10:**
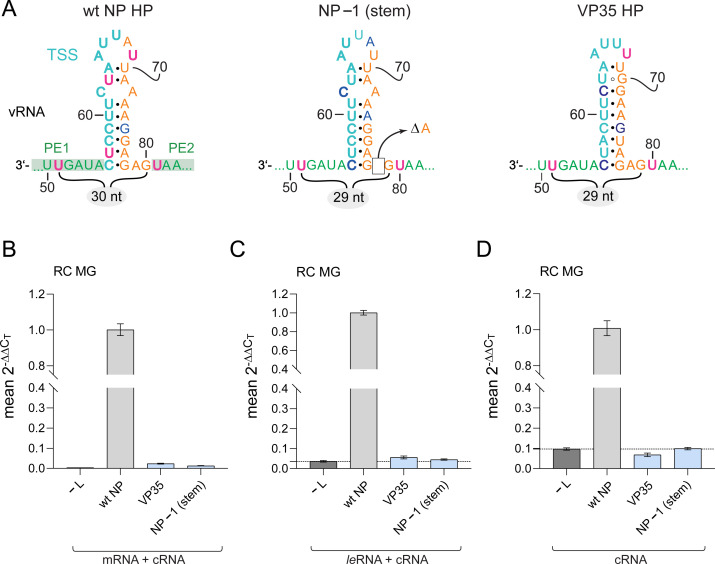
qRT-PCR analysis of *leader*RNA synthesis from MG templates that deviate from hexamer phasing. (**A**) Illustration of RC MG variants, either encoding the wt NP HP, a derivative thereof with a single nt deletion, construct NP-1 (stem), or a variant in which the NP HP was replaced with the corresponding hairpin of the VP35 gene; in the latter two constructs, the distance between nt 51 and 80 is not a multiple of 6 (29 nt). For detailed information on color code see legend to Fig 1B; ΔA, deletion of a single A residue. (**B**-**D**) qRT-PCR analysis (performed as described in Fig 7 and S2B Fig, strategy 2) of MG variants illustrated in panel A; (**B**) mRNA+cRNA level, (**C**) *le*RNA+cRNA level, (**D**) cRNA level. Mean 2^-ΔΔC_T_^ values derive from 3 independent experiments with 3 technical replicates each.

The NP hairpin represents the major part of the spacer region separating PE1 and PE2 of the leader promoter (Fig 1B). It was recently shown that hexamer phasing in the EBOV 3’-leader promoter is not only crucial for replication [44] but also for initiation of viral transcription [18]. This raised the question whether hexamer phasing affects *leader*RNA synthesis as well. To address this issue, we used two MG variants deviating from hexamer phasing in the promoter, one carrying the NP hairpin with a 1-nt deletion near the stem and a variant in which the NP hairpin was replaced with the corresponding element of the VP35 gene (Fig 10A). In contrast to the wt (NP) hairpin conforming to hexamer phasing (with 30 nt between genome pos. -51 to -80), both mutant MGs comprised only 29 nt in this region. As shown by qRT-PCR, the two mutant MGs were defective in antigenome (cRNA), mRNA and *leader*RNA synthesis (Fig 10B–10D). We conclude that hexamer phasing is generally essential for initiation of RNA synthesis in the leader promoter.

### Abortive trailer transcripts

We also identified putative abortive trailer transcripts in our RNA-Seq libraries from EBOV-infected and MG-transfected cells, attributable to prematurely terminated vRNA synthesis initiated on antigenomic cRNA. *Trailer*RNAs can be differentiated from *leader*RNAs by sequence differences at nt positions 15/16, 22/23 and 25/26 (Zaire EBOV genome, NC_002549.1). *Trailer*RNAs were less (~20-fold) abundant than *leader*RNAs in RNA-Seq libraries (cf. Figs 8B and S7C). As for *leader*RNA, the vast majority of *trailer*RNAs were initiated at position 2 of the template RNA (cf. S7A and S7B Fig). We observed a non-significant trend toward increased *trailer*RNA reads in libraries from MG-transfected cells without VP30 coexpression (S7C Fig), consistent with elevated cRNA template synthesis in the absence of VP30 (Fig 7B). Relative to *leader*RNAs, the fraction of *trailer*RNA reads in the length range of ~20–45 nt was increased compared with the nt ~60 to 80 fraction (cf. S6 and S8 Figs). This change was more pronounced for RNA libraries derived from MG-transfected compared with those from EBOV-infected cells (cf. S8A and S8B–S8D Fig). For MG-transfected cells, the patterns were essentially identical in the presence and absence of VP30 (S8B–S8D Fig versus S8E–S8G Fig). As observed for the *leader*RNAs, reads terminating in the nt 70 region showed an increased 3’-terminal deviation from the genome-encoded sequence (S8 Fig).

### Leader transcripts do not affect innate immunity

**Fig 11:**
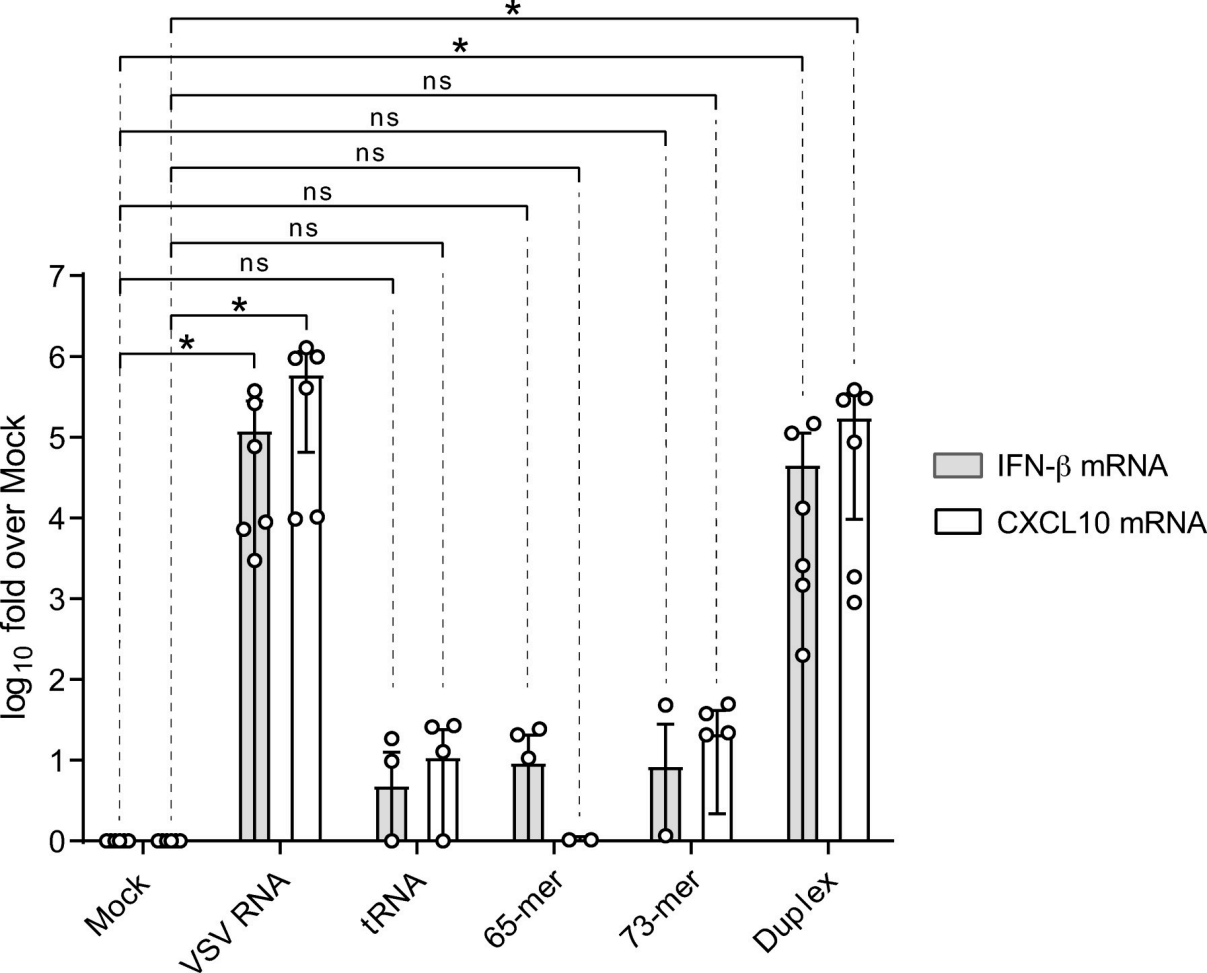
Analysis of innate immunity induction in HEK293 cells upon transfection with chemically synthesized, 5’-triphosphorylated EBOV *leader*RNA mimics (65-mer, 73-mer), using mRNA levels for IFN-β and CXCL10 as readout; mRNA levels were determined by qRT-PCR. Vesicular Stomatitis Virus (VSV) genomic RNA isolated as described [81] and a validated RIG-I-activating duplex RNA (3P-G/AS G24; [49]) were used as positive controls and bulk tRNA from *Saccharomyces cerevisiae* as negative control. In the mock control, transfection was performed with H_2_O instead of RNA. The graph with mean values and standard deviations is based on six independent experiments each. Statistical significance was evaluated using the Wilcoxon test *, p < 0.05; ns, not significant. For sequence and structure of the 65-mer, 73-mer and duplex 3P-G/AS G24, see S2 Table; for methodological details, see S1 Text.

T7-transcribed, 5’-triphosphorylated leader RNAs from NNS viruses, such as measles, rabies, vesicular stomatitis virus (VSV) or Newcastle disease virus (NDV) were reported to induce the antiviral type I interferon (IFN) system by activating the virus sensor RIG-I [46,47,48]. Likewise, an EBOV leader transcript mimic comprising nt 2–56, synthesized *in vitro* by T7 RNA polymerase as well, showed RIG-I-dependent activation of the IFN-β promoter [47]. It remained unclear, however, whether side products generated by T7 transcription were responsible for this observation. We revisited this issue by analyzing effects of chemically synthesized *leader*RNA mimics with 5’-triphosphate ends on innate immunity. We first tested this with a chemically synthesized 65-meric *leader*RNA mimic whose 5’-terminal nucleotide is complementary to the very 3’-end of the genomic RNA (S2 Table). The 65-mer was selected because its 3’-terminus was frequently represented in the small RNA-Seq libraries derived from EBOV-infected cells. After finding that *leader*RNAs are initiated at position 2 (Fig 4D), we further included a 73-mer mimicking *leader*RNA initiated at the 2nd 3’-terminal genome position, which represented the major size range of *leader*RNAs (Fig 2A). A validated synthetic duplex RNA, previously demonstrated to elicit RIG-I-mediated immune responses ([49] see S2 Table), as well as VSV RNA served as positive controls and tRNA as a negative control. Upon transfection of HEK293 cells with VSV RNA and the duplex RNA control, we observed significant increases in the mRNA levels of IFN-β and the cytokine CXCL10. By contrast, immunostimulatory effects were neither seen with tRNA nor with the 65- and 73-meric *leader*RNA mimics (Fig 11). Successful transfection of HEK293 cells with the *leader*RNA mimics was confirmed by qRT-PCR (S9 Fig). Also, in experiments where we cotransfected increasing amounts of the 65-mer with a constant amount of VSV RNA, we found the intracellular IFN-β and CXCL10 mRNA amounts to be elevated at constant levels irrespective of the 65-mer concentration in the transfection mix (S10 Fig). The absence of a significant inhibitory effect exerted by increasing amounts of 65-mer on VSV RNA-induced immunity argues against the possibility that *leader*RNAs might act as a bait to sequester RIG-I and shut down the protein’s activity.

## Discussion

In the present study, we identified and characterized, for the first time, abortive EBOV *leader*RNAs (and *trailer*RNAs). *Leader*RNAs, as cRNAs [16] are initiated at the penultimate 3’-nucleotide of the EBOV genome. This feature of a single initiation site at the genome’s 3’-end is shared with other NNS viruses such as VSV (*Rhabdoviridae*) and SeV (*Paramyxoviridae*) that, however, initiate *leader*RNAs and cRNAs at the genome’s very 3’-end [50,51], not at the penultimate nucleotide.

The widely accepted “single-entry and stop-start” transcription model proposes that NNS viruses synthesize *leader*RNAs antisense to the genome 3’-leader sequence before transcription initiation at the TSS representing the first GS signal [32–34,52]. For VSV and SeV, it was further proposed that leader transcripts are abortive cRNA products that are terminated because of failure to immediately encapsidate the nascent RNA by nucleoprotein (NP). The model predicts that RdRp, subsequent to abortive *leader*RNA synthesis, initiates transcription at the first gene and becomes programmed to recognize the GS/GE signals and to cap and polyadenylate the individual mRNA transcripts [51; reviewed in 32,33,35]. This notion received support from studies using transcription-competent VSV cores or purified SeV preparations [51,53], where the authors reported roughly equimolar amounts of *leader* and NP mRNA, respectively. However, a VSV mutant strain (polR1) produced about twofold more NP mRNA than *leader*RNA, suggesting the possibility that the viral polymerase complex might have the capacity to also initiate RNA synthesis directly at the TSS of the NP gene [51]. Indeed, more recent studies on VSV provided evidence that, within cells, distinct replication and transcription complexes exist that initiate RNA synthesis at separate sites in the 3’-*leader* promoter, i.e., at the genome 3’-end and at the more internally positioned TSS preceding the first gene, respectively [54,55]. For VSV, isolated replication complexes comprised L, P (the functional homolog of EBOV VP35) and N, whereas transcription complexes consisted of L, P and three host proteins, EF-1A, HSP60 and substoichiometric amounts of guanylyltransferase. *In vitro*, the isolated replicase or the RNP isolated from the virus, but not the purified transcriptase, synthesized *leader*RNA [55]. Interestingly, Whelan and Wertz [54] provided evidence that detergent-activated VSV virions give rise to sequential *leader*RNA synthesis followed by N mRNA transcription, thus contrasting the intracellularly observed direct initiation of RdRp at the TSS of the N gene. Thus, pure *in vitro* studies are at risk of not representing the intracellular situation. The aforementioned findings indicate that *leader*RNA synthesis is not necessarily a prerequisite for each transcription initiation event. Rather, these findings support the idea that different functional forms of RdRp complexes interact differently with the 3’-leader promoter, initiating RNA synthesis at different locations. Two distinct RdRp complexes are also conceivable for EBOV, considering that its unique viral transcription factor VP30 supports transcription but is completely dispensable for replicative RNA synthesis.

We analyzed *leader*RNA levels in EBOV-infected and MG-transfected systems (Figs 3 and 4). Quantification of mRNA:*leader*RNA ratio revealed a ~10-fold (based on C_T_ values only) and ~20 to 30-fold (based on qRT-PCR standard curves) NP mRNA excess over *leader*RNA during virus infection and a ~70- to 190-fold excess, respectively, of Rluc mRNA over *leader*RNA in MG-transfected cells (after 48 h in both cases). Considering that the MG setup provides constant excess amounts of NP molecules, the higher mRNA to *leader*RNA excess seems consistent with the abovementioned model predicting that termination of *leader*RNA synthesis is suppressed at high NP levels. Yet, the abovementioned model would also imply that replication is boosted in the MG system as most *leader*RNAs are elongated to cRNAs. However, the MG system yields ~100-fold higher amounts of mRNA than cRNA [18], suggesting that other factors than NP availability, such as the levels of unphosphorylated VP30, determine the relative levels of viral RNA synthesis products in the MG system. The excess of NP mRNA over *leader*RNAs, far off equimolarity also in the infection model, is consistent with *leader*RNAs representing aborted cRNAs, but argues against their synthesis as obligatory pre-products required to initiate mRNA transcription. The only argument to rescue the hypothesis of linked and equimolar synthesis of *leader*RNA and NP mRNA for the EBOV system is to assume that *leader*RNAs are degraded more rapidly than NP mRNA. This possibility cannot be entirely excluded at present, although coherent evidence in favor of this possibility is lacking, complicated by the fact that, for other NNS viruses, lower *leader*RNA levels in cells versus *in vitro*-transcription assays and different levels in different cell types were observed [50,53]. Against this background, we cannot rule out that the quantitative differences in mRNA excess over *leader*RNA in EBOV-infected HuH7 versus MG-transfected HEK293 cells also included cell type-specific contributions. In HEK293 cells infected with Borna disease virus (BoDV), which however replicates in the nucleus compared with cytoplasmic EBOV replication, *leader*RNAs were detected in equimolar amounts to the first mRNA [52]. This might be taken as evidence against rapid *leader*RNAs degradation in this cell line that we also used for our MG system. However, not excludable is the possibility that BoDV *leader*RNAs were produced in excess over the first mRNA, but were degraded more rapidly than mRNA, such that the steady-state levels of both RNA species were (fortuitously) equimolar. Altogether, the most straightforward interpretation of our findings is, analogous to VSV, the presence of distinct replicase and transcriptase complexes of EBOV RdRp that interact differently with the 3’-leader promoter and initiate RNA synthesis at distinct locations.

The qRT-PCR data in Fig 7D indicate that *leader*RNA levels in the MG system are similar to, though somewhat higher than cRNA levels. Both levels increase in the absence of VP30 (Fig 7D; for *leader*RNAs, see also Fig 8B), conditions where mRNA synthesis is shut down (Figs 7A and 8C). This also lends support to the notion that the absence of VP30 favors the replicase form of EBOV RdRp that synthesizes *leader*RNAs and cRNAs.

We assume that EBOV RdRp, as in other NNS virus models, gains access to replication and transcription promoter elements via binding to the genome 3’-end. Consistently, the present study suggests that, for EBOV transcription initiation, the viral polymerase needs to contact the very 3’-end directly (conceivably as part of the transcription promoter). The genome variant Δ1 lacking the G_1_ residue showed a ~fourfold decrease in reporter activity relative to the wt genome (Fig 5C) in the context of a RD MG backbone. In contrast, variant Δ1 was as active as the wt construct in the RC MG system (Fig 5B and 5D–5F) and retained ~70% of replication activity in another study [16]. This observation can be explained as follows: as long as viral replication is enabled, the 3’-terminal residue (nt 1) can be added to newly synthesized vRNAs by the viral polymerase, possibly in a terminal transferase reaction [16]. Furthermore, only low amounts of functional nucleocapsids are thought to be spontaneously assembled from MG vRNAs produced by non-viral RNA polymerases (such as T7 RNAP) and NP in the absence of viral replication [56], explaining the low levels of reporter activity in RD MGs [14,18,40]. Thus, viral replication in RC MG systems will not only generate vRNAs carrying the G_1_ residue, but those vRNAs will also be part of more functional nucleocapsids and thus outcompete the pre-synthesized T7 vRNAs as RNA templates for viral mRNA synthesis. This is expected to have masked those transcriptional defects that were revealed in the RD MG system (Fig 5C). Hence, we argue that the RD MG system is better suited to analyze the effect of genome 3’-end variations on transcription. It should be mentioned that RD MGs, although giving rise to rather low reporter activities, provide biologically relevant readouts that are fully in accordance with results obtained with RC MGs, as shown for the dependence of viral transcription on VP30 and hexamer phasing [14,18,40,41]. The +G variant was also more active than the wt construct in the RC MG system, and here most evident on the cRNA level (Fig 5B and 5D–5F). This variant showed only ~40% activity in the RD MG system (Fig 5C). This can be explained by the +G variant boosting the synthesis of cRNA and vRNA in the RC MG system, thus indirectly stimulating mRNA transcription by increasing the amount of vRNA templates. In summary, our findings demonstrate that the EBOV RdRp strongly prefers genome 3’-G_1_CC ends in the transcription mode, whereas replication is largely permissive to the absence of G_1_ and even seems to be enhanced by adding another G residue to the 3’-end. This suggests that genomes packaged into infective EBOV particles carry 3’-G_1_CC ends to support primary transcription during early infection.

The aforementioned findings imply mechanistic differences in 3’-leader promoter recognition by EBOV RdRp in the replication versus transcription mode. The second genome nt (C_2_), however, is essentially indispensable for transcription and cRNA synthesis [16]. It may be part of the replication and transcription promoter or it may be crucial for NP phasing. For paramyxoviruses, the only other family of NNS viruses that utilize bipartite replication promoters [57,11], a proper spacing and/or NP phasing of the genome 3’-end and the TSS was inferred to be important for transcription. In the paramyxoviruses SeV and HPIV3, transcription still occurred when most of the leader and/or PE2 were deleted, provided that a certain NP phase at the TSS was maintained [58–61]. In a recent study we showed that EBOV transcription was eliminated by a dinucleotide deletion (nt 54 and 55) directly 3’ of the TSS. These 2 nt may either be part of the transcription promoter or may have changed NP phasing of the leader by positioning the TSS closer to the genome 3’-end [18]. We have demonstrated here and previously [18] that hexamer phasing in the PE1-PE2 spacer is obligatory for mRNA, cRNA and *leader*RNA synthesis. We recently proposed a model according to which productive binding of the EBOV polymerase to the 3’-terminal region of the genome, as a prerequisite for any polymerization activity, requires that NP molecules are assembled in the correct register in the region of nt 51 to 80 and further upstream [18]. Based on this model, termination of *leader*RNAs in the region of nt ~ 60–80 may then be caused by inefficient removal of NP from the template RNA in this region or inefficient NP encapsidation of nascent *leader*RNAs. For viral transcription, NP molecules have to be removed from the TSS (nt 56 to 67), and this may be achieved with the help of VP30 and/or VP35 [62,63]. A key role of NP in *leader*RNA termination is in line with the lack of any clear RNA sequence or structure signature for termination of *leader*RNAs (Figs 6 and 9), which is in contrast to the GE signal sequences at internal genes.

For VSV, mutant NP was reported to suppress *leader*RNA termination and to favor *leader*RNA readthrough [64], also suggesting a key role of NP in *leader*RNA termination. It is thus not too farfetched to consider the possibility that binding of NP molecules to the UN_5_ hexamers in PE2 of EBOV may provide a barrier that induces termination of *leader*RNAs in the region preceding PE2. However, this can be excluded as well since *leader*RNAs derived from the Δ5’ spacer MG cover the same size range as those derived from the wt NP MG and thus predominantly terminate within PE2 (Fig 9D). For the VSV mutants that showed enhanced readthrough at the *leader*RNA termination site, the phenotype could be assigned to an Arg179His mutation in the VSV NP [65], while mutations in non-coding RNA genome regions were not identified to contribute to the phenotype. The mutation in NP went along with changed ATP requirements for viral RNA synthesis *in vitro*. Second-site suppressors of the leader readthrough phenotype were assigned to a mutation in L, whereas no suppressor mutations were identified in the P protein. The authors proposed that *leader*RNA termination is caused by an ATP-dependent interaction between the template-associated NP and the L subunit of the P–L polymerase complex [65].

The detection of abortive trailer transcripts (S7B, S7C and S8 Figs) shows that premature termination is not a feature restricted to initiation of viral RNA synthesis at the leader promoter, and thus seems to be an inherent mechanistic feature of the EBOV replicase, possibly triggered by interaction with template-associated NP molecules as discussed above for VSV. This finding raises new questions, for example regarding the possible function of *trailer*RNAs in replication, or why there is a shift toward shorter abortive transcripts (~20 to 40 nt) relative to *leader*RNAs, and why this shift is more pronounced in MG-transfected than EBOV-infected cells (S8 Fig). For Influenza A virus, short RNAs (~ 20–27 nt in length), most likely with 5’-triphosphate ends and corresponding to the 5’-ends of the genomic (vRNA) segments, were identified by RNA-Seq [66,67]. These small viral RNAs (svRNAs), which formally correspond to the EBOV *trailer*RNAs identified here, were found to accumulate when viral transcription was switched to replication [66,67]. SvRNAs were found to physically associate with RdRp, and antisense oligonucleotides targeting svRNAs led to a stronger reduction of vRNA than of mRNA or cRNA. It was thus proposed that svRNAs may reprogram RdRp from a transcriptase to a replicase, or might reconstitute a double-stranded RNA promoter *in trans* that allows RdRp to access the cRNA 3’-end for initiation of vRNA synthesis [66].

For pneumoviruses, such as RSV, the genomic leader includes U-rich sequence motifs preceding (3’ of) the TSS. Such sequence elements were previously shown to be important for transcription in some of the NNS viruses [68–71]. As GE signals contain U stretches as well, U-rich sequences preceding the TSS were considered as alternative leader termination signals. However, in contrast to mRNA transcripts, *leader*RNAs are not precisely terminated at a defined position, suggesting that the polymerase is in a rather unprocessive mode during their synthesis [36,50,53,72–74]. We experimentally tested the presence of cryptic GE signals in the EBOV leader, but were unable to detect such GE signals within the EBOV leader-NP-hairpin region (Fig 6) that might explain *leader*RNA termination. Thus, we rule out that a sequential stop-start transcription mechanism, as operational at internal EBOV genes, also applies to the EBOV leader-NP junction. Similar conclusions were drawn for the VSV leader-N gene junction [35]. The majority of EBOV *leader*RNAs terminate after RdRp has synthesized the first eight purines complementary to the transcription start sequence (Fig 1B, light blue nt; Fig 2A). Beyond this point, the genome template codes for a stretch of 11 nt only consisting of A and U residues including a U_4_ stretch (Fig 1B). We observed an increased proportion of mutations in *leader*RNAs terminating in this region, which pertains to RNAs from EBOV-infected and MG-transfected cells. This included a fraction of *leader*RNA reads with additional non-templated U residues and another fraction with an unrelated 3’-terminal sequence stretch that can be attributed to RdRp using the nascent transcript instead of the genomic RNA as template in a snap-back mechanism (S11 Fig; also observable in the *trailer*RNA libraries). These non-canonical, partly non-templated *leader*RNA termination products were generally confined to the nt 70 region, where its proportion was increased in the MG system relative to EBOV-infected cells (S6 Fig and S4 Table). Beyond these subtle differences observed between EBOV-infected and MG-transfected cells, the findings generally suggest differences in the mode of termination in the nt 20–40 region versus the nt 70 region at which RdRps may have longer dwell times. Evidently, these non-canonical *leader*RNAs are footprints of RdRp complexes that lost their grip on the template RNA but stayed in the polymerization mode for some time. It will also be interesting to see if the incorporation of non-templated nucleotides into *leader*-RNAs mechanistically relates to cotranscriptional editing (addition of non-template-encoded residues) at homooligomeric sequence stretches in the EBOV genome, such as the genomic U stretch at the GP mRNA editing site [31].

EBOV *leader*RNAs might be sheer by-products of replication. Yet, leader transcripts in other NNS viruses were suggested to be recognized by RIG-I, thus inducing an innate immune response [46–48]. Conversely, there are several examples of short leader/trailer RNAs subverting RIG-I mediated immune responses as a potential escape mechanism. In RSV infection, *leader*RNAs were suggested to revoke RIG-I recognition by binding to the cellular La autoantigen [75]. Trailer RNA transcripts of SeV were reported to bind to the cellular RNA binding protein TIAR, to exert anti-apoptotic effects and to avert the cellular stress granule response [76]. For RSV trailer RNAs, an involvement in subverting stress granule responses was proposed as well [77]. Considering that EBOV *leade*rRNAs are initiated opposite to genome position 2, it is conceivable that they anneal to non-encapsidated genome 3’-ends during RNA synthesis, generating a duplex with 1-nt 3’-overhang that is unfavorable for RIG-I activation [49]. The only other virus initiating RNA synthesis at position 2 is the Tacaribe virus, a segmented negative strand RNA virus of the arenavirus family. In a proposed prime-realign mechanism, the internally (at pos. 2) initiated RNA dinucleotide 5’-pppGpC slips backwards on the template RNA to generate a 1-nt 5’-overhang which prevents RIG-I recognition [78,79]. A 5’-triphosphorylated EBOV leader transcript corresponding to nt 2–56 (Fig 1B), synthesized by T7 RNA polymerase *in vitro* and purified by denaturing PAGE, was reported to elicit some RIG-I activation [47]. Here we used chemically synthesized EBOV *leader*RNA mimics, including a 73-mer with identical 5’-ppp end and representing a major EBOV *leader*RNA length variant, but did not observe any stimulatory or inhibitory effects on innate immunity (Fig 11). The reason for this discrepancy is unclear at present. It cannot be ruled out that copurified side products of T7 RNA polymerase were responsible for stimulation of innate immunity in the previous study [47]. Also, the previously analyzed 55-mer is not a major *leader*RNA length species according to our RNA-Seq analysis and may thus not be representative of the variant spectrum of EBOV *leader*RNAs, taking into account that different *leader*RNA variants might have different biological effects. The same considerations also apply to other NNS viruses. For example, in the study that reported shielding of RSV *leader*RNA from RIG-I by the cellular La protein, a *leader*RNA mimic of 44 nt was employed [75], although the length spectrum of RSV *leader*RNAs was later shown to peak at ~25 nt [36].

In summary, we demonstrated, for the first time, the existence of EBOV *leader*RNAs (and *trailer*RNAs), mapped their borders, identified hexamer phasing in the leader promoter as a prerequisite for their synthesis, could largely exclude RNA sequence and structure constraints and cryptic gene end signals as determinants of their termination, and were able to largely discard the possibility of a *leader*RNA role in IFN induction. We showed that *leader*RNA amounts are substantially lower than those of the first mRNA and provided evidence that *leader*RNAs, and also *trailer*RNAs, are synthesized by EBOV RdRp in its replicative mode. Additionally, we observed differential genome 3’-end constraints in viral transcription versus replication, providing first evidence for differences in 3’-leader promoter recognition by the filoviral RdRp in the transcriptional versus replicative mode. Our results favor the model of distinct replication and transcription complexes that directly initiate RNA synthesis at separate sites in the 3’-*leader* promoter, i.e., at the genome 3’-end and at the more internally positioned TSS preceding the first gene, respectively, as demonstrated for VSV [54,55]. VP30, in line with previous findings, favors 3’-leader promoter recognition in the transcription mode of RdRp. As basal transcription is initiated at the genuine TSS also in the absence of VP30 (at least in the MG system), the protein is concluded to change the equilibrium between the replicase and transcriptase states.

## Supporting information

S1 Text Methods description. (DOCX)

S1 Table Primers and DNA fragments used in this study for the construction of mutant minigenomes. (DOCX)

S2 Table Synthetic RNA oligonucleotides and T7 transcripts used in this study. (PDF)

S3 Table RNA-seq data analysis summary. For methodological details, see S1 Text, paragraph "RNA-Seq Analysis".
(XLSX)

S4 Table RNA-seq read numbers and read classification for *leader*RNAs and *trailer*RNAs. For more information, see S11 Fig.
(XLSX)

S1 Fig PCR-based strategies that were used for the construction of mutant minigenomes. (A) Inside-out primer deletion mutagenesis. (B) Overhang/inside-out primer insertion mutagenesis. (C) Complementary primer mutagenesis for introduction of insertions or substitutions. In approaches A and B the entire plasmid is amplified with 5‘-phosphorylated primers that introduce the desired insertions/deletions, followed by circularization of PCR products and template removal by Dpn I treatment before bacterial transformation. In approach C phosphorylation of 5’-ends and ligation are carried out by bacterial enzymes after DNA transformation.
(DOCX)

S2 Fig qRT-PCR strategies applied to the quantification of viral RNA species. (DOCX)

S3 Fig Mathematical approach to estimate mRNA:*leader*RNA ratios in EBOV-infected HuH7 cells. (DOCX)

S4 Fig qRT-PCR using standard curves. (A) qRT-PCR standard curves for the individual standard RNAs. The molar amount of the respective RNA fragment introduced into the qRT-PCR reaction is given on the x-axis in logarithmic scale. The equation of the linearized calibration curve is shown as inset. (B) RT-PCR products using total RNA from EBOV-infected or MG-transfected cells and the different primer pairs according to strategy 4 were analyzed on a 2% agarose gel; the PCR products obtained after 40 PCR cycles were stained with GelRed; M, 100 bp ladder (CytoGen GmbH, Sinn, Germany) used as size marker. (C) Example calculations of NP mRNA:*leader*RNA amounts in EBOV-infected cells based on the standard curve for the 73-meric *leader*RNA mimic, and Rluc mRNA:*leader*RNA amounts in MG-transfected cells based on the standard curve for the 65-meric *leader*RNA mimic.
(PDF)

S5 Fig **RNA-Seq reads representing Renilla mRNA 5’-ends in poly(A) RNA fractions** derived from cells transfected with the Δ5’ spacer MG, the NheI HP MG and the wt MG, either in the presence (A to C) or absence (D to F) of VP30. Panels A and D, identical to Fig 8D and 8E, are shown for comparison. Mean values (± SEM) are based on 2 to 4 biological replicates each. The dashed vertical line marks the canonical EBOV transcription start site (TSS). For more details, see legend to Fig 2B of the main text and S3 Table.
(DOCX)

S6 Fig **Comparative RNA-Seq analysis of *leader*RNA lengths in RNA libraries derived from (A) EBOV-infected cells and (B-G) cells transfected with MGs** (wt NP HP, NheI HP, Δ5’ spacer; illustrated in Fig 8A of the main manuscript) in the presence (B-D) or absence (E-G) of VP30. Mean values (± SEM) are based on 3 to 5 biological replicates each. Red bars or red part bars indicate reads with not more than 1 non-templated nt (= 1 mismatch) or 1 indel (insertion or deletion of 1 nt in the segemehl alignment) in the 3’-terminal 15 nt (canonical reads); blue bars or blue part bars indicate reads with at least 2 mismatches or indels in the 3’-terminal 15 nt. (error-prone reads). For more details, see legend to Fig 2A of the main text as well as S3 and S4 Tables.
(DOCX)

S7 Fig *Leader* and *trailer* transcript 5’-ends determined by RNA-Seq for EBOV-infected HuH7 cells and MG-transfected HEK293 cells. (A, B) For the MGs (wt NP HP, NheI HP and Δ5’ spacer), read numbers in the presence (+) as well as absence (-) of VP30 are given; *spike-in*: a synthetic 5’-triphosphorylated 65-meric *leader*RNA (sequence in S2 Table) was added to a small RNA preparation isolated from non-infected/non-transfected (mock-treated) HEK293 cells to control for authentic 5’-end representation in RNA-Seq libraries. The color code representing transcription starts at the first, second and third genome end position is indicated on the right. The number of biological replicates (n) is indicated above each column (for details, see S3 Table). (C) Comparison of *trailer*RNA read abundance in the small RNA-Seq libraries (< 200 nt) from EBOV-infected cells and from cells transfected with the different MG variants in the presence versus absence of VP30. Regarding the MG libraries, the difference between read numbers in the +VP30 versus –VP30 samples is neither significant if the three +VP30 and the three–VP30 MG libraries are each pooled (p = 0.211, Welch’s *t* test) nor if the individual MGs constructs are considered (p = 0.459 for wt NP HP, p = 0.451 for NheI HP, p = 0.767 for Δ5’ spacer; Welch’s *t* test). For number and details on biological replicates, see S3 Table.
(DOCX)

S8 Fig **Comparative RNA-Seq analysis of *trailer*RNA lengths in RNA libraries derived from (A) EBOV-infected cells and (B-G) cells transfected with MGs** (wt NP HP, NheI HP, Δ5’ spacer; illustrated in Fig 8A of the main text) in the presence (B-D) or absence (E-G) of VP30. Mean values (± SEM) are based on 3 to 5 biological replicates each. Red bars or red part bars indicate reads with not more than 1 non-templated nt (= 1 mismatch) or 1 indel (insertion or deletion of 1 nt) in the 3’-terminal 15 nt (canonical reads); blue bars or blue part bars indicate reads with at least 2 mismatches or indels in the 3’-terminal 15 nt (error-prone reads). For more details, see legend to Fig 2A of the main text and S3 and S4 Tables.
(DOCX)

S9 Fig qRT-PCR detection of transfected EBOV *leader*RNAs (65-mer, 73-mer). Following transfection of HEK293 cells with the RNAs specified at the bottom, the medium was removed and cells were gently washed with PBS. Subsequently, total cellular RNA was isolated and qRT-PCR was performed with the primer pair specific for the *leader*RNA 65- and 73-mer (for details, see S1 Text, paragraph "qRT-PCR assay for innate immune induction").
(DOCX)

S10 Fig Innate immunity response (inferred from increases in the levels of mRNAs coding for IFN-β and CXCL10) upon cotransfection of constant amounts of VSV RNA (50 ng/well) and increasing amounts of synthetic *leader*RNA (*le*RNA) 65-mer or bulk tRNA from yeast. qRT-PCR reactions were performed with primer pairs specific for IFN-β mRNA (top, sky blue columns), CXCL10 mRNA (dark blue columns), VSV RNA (light green bars) and the EBOV *leader*RNA 65-mer (pink columns). The slight reduction in IFN-β and CXCL10 mRNA levels at 500 and 750 ng *leader*RNA can be attributed to slightly reduced cellular uptake of VSV RNA at excess amounts of the *leader*RNA competitor in the transfection mix (see VSV graph, light green bars). For experimental details, see S1 Text, paragraph "qRT-PCR assay for innate immune induction".
(DOCX)

S11 Fig Examples of categorized *leader*RNA reads and their predicted structures. (DOCX)
