# Supplementary material for: Identification and characterization of short leader and trailer RNAs synthesized by the Ebola virus RNA polymerase
Source: PLoS Pathog. 2021 Oct 26;17(10):e1010002. doi: 10.1371/journal.ppat.1010002 (PMC8547711; doi:10.1371/journal.ppat.1010002)
Supplement: S1 Text — (DOCX) [file ppat.1010002.s001.docx]

**SUPPORTING INFORMATION**

**Identification and characterization of short leader and trailer RNAs synthesized by the EBOV RNA polymerase**

Simone Bach^1^, Jana-Christin Demper^1^, Paul Klemm^2^, Julia Schlereth^1^, Marcus Lechner^2^, Andreas Schoen^3^, Lennart Kämper^4^, Friedemann Weber^3^, Stephan Becker^4^, Nadine Biedenkopf^4,^*, Roland K. Hartmann^1,^*

1 Institut für Pharmazeutische Chemie, Philipps-Universität Marburg, Marburg, Germany

^2^ Zentrum für Synthetische Mikrobiologie, Philipps-Universität Marburg, Marburg, Germany

^3^Institut für Virologie, Justus-Liebig-Universität Gießen, Gießen, Germany

4 Institut für Virologie, Philipps-Universität Marburg, Marburg, Germany

*nadine.biedenkopf@staff.uni-marburg.de; *roland.hartmann@staff.uni-marburg.de

**Cloning of replication-competent (RC), replication-deficient (RD) monocistronic and RC bicistronic MG variants**

3'-leader monocistronic MG variants were derived from the replication-competent (RC) EBOV minigenome pANDY 3E5E. Mutant derivatives of the native leader 3’-terminus were generated by either deletion of the first 3’-G nucleotide (p3E5E_3’Δ1), deletion of the 3’-terminal GC dinucleotide (p3E5E_3’Δ2) or elongated by an additional G residue (p3E5E_3’+1) using Dpn I-based site-directed mutagenesis techniques (see S1 Fig). Cloning primers are specified in S1 Table. Corresponding RD MGs were based on a pANDY 3E5E derivative lacking the terminal 55 nucleotides of the antigenome 3’-trailer replication promoter [1] using the same primers and mutagenesis setup as described for the RC MG 3’-leader variants. Variants of the wt pANDY 3E5E MG comprising a destabilized NP hairpin structure at the first NP GS signal (p3E5E_NheI NP) or lacking 12 spacer nucleotides that separate the NP GS signal from replication PE2 (p3E5E_Δ5’ spacer) were described previously [2,3]. This also pertains to monocistronic RC MGs harboring spacer sequences deviating from hexamer phasing [p3E5E_VP35; p3E5E_NP-1 (stem)] [2-4]. EBOV leader and trailer sequences correspond to the EBOV Mayinga (Zaire, 1976) sequence, GenBank accession no. AF086833. All constructs were verified by DNA sequencing.

*Cloning of RC bicistronic MG variants*

The wt bici MG (pA-3E5E-RLuc-FFLuc) was constructed using a 5-step-cloning strategy (see S1 Table) based on the plasmids pA-3E5E-GLuc-GFP (used as backbone and harboring the EBOV leader, trailer, and the abovementioned UTRs), pCAGGS_Luc2 (used for exchange of GFP with FFluc) and wt pANDY 3E5E (used for exchange of GLuc with RLuc). Variants carrying the NP or VP40 instead of the VP35 5’-UTR hairpin structure at the 2nd cistron were cloned by restriction cloning using GeneArt Strings DNA Fragments (Thermo Fisher Scientific [TFS]). NP GE mutants were obtained by site-directed mutagenesis. Here, the GE signal was inactivated by mutating 3 out of 6 U residues to adenosines (Fig 6A) as reported previously [5].

**qRT-PCR**

qRT-PCR strategy 1 (S2A Fig) was used to quantify *leader*RNA+cRNA, cRNA and NP mRNA in RNA preparations derived from EBOV-infected cells. Strategy 2 (S2B Fig) was applied to quantify *leader*RNA+cRNA, mRNA + cRNA and cRNA alone (PCR primer pair shared with *leader*RNA+cRNA) in RNA preparations from MG-transfected cells. 5’-ends of the RT primers (dashed vertical lines and nt position) are indicated panel A and B. Strategy 3 [4,6] was used to quantify vRNA, cRNA+mRNA and cRNA in RNA preparations from MG-transfected cells. Strategy 4 was applied in the qRT-PCR experiments involving standard curves. RT primers are highlighted in red in S2 Fig.

*Strategy 1*

(EBOV infection samples; S2A Fig): 1 µg (EBOV infection) of total RNA were used for reverse transcription (RT) in a total reaction volume of 20 µL using the RevertAid H Minus Reverse Transcriptase (Thermo Fisher Scientific [TFS]) according to the manufacturer´s recommendations. An initial denaturing step at 80°C for 5 min was included for denaturation of structured RNAs. RT primers were: RT_leader+cRNA: 5’-ATC TTC CTC ATA GTT ATT CGC ACA CA-3’; RT_NP_mRNA: 5’-TTT TTT TTT TTT TTT TTT TTT TTT TTT TTT TTT TTC TTA ATT ATA AAA CG-3’; RT_cRNA_s1/s2: 5’-CAA ACC AGG TGT GAT TAC AGT AAC AAT T-3’. ~40 ng of the prepared cDNA (4 µL of a 1:5 dilution) were used for the qRT-PCR reaction that was performed in technical duplicates on a BioRadiQ5 system (BioRad) using the 2x ABsolute QPCR Capillary Mix, SYBR Green (TFS) and the respective forward and reverse primers (5 µM each) in a total volume of 20 µL. Primers for PCR amplification of *leader*RNA+cRNA: RT_leader+cRNA (see above) and qPCR_leader+cRNA: 5´-GCC GGA CAC ACA AAA AGA AAG AA-3’, yielding a 66 bp product; primers for PCR amplification of cRNA: RT_cRNA s1/s2 (see above) and qPCR_leader+cRNA, resulting in a product of 139 bp; primers for PCR amplification of NP mRNA; primers RT_NP_mRNA (see above) and qPCR_NP_mRNA 5 ‘-GTA ACT CAA TAT TCT AAC TAG CGA TTT ATC TAA ATT AAA TTA CA-3' (product length 141 bp). The temperature profile was 15 min at 95°C, followed by 30 cycles à 10 s at 95°C / 20 s at 60°C / 15 s at 72°C. Calculation of mRNA:*leader*RNA ratio was performed as depicted in S3 Fig.

*Strategy 2*

(MG-transfected samples; S2B Fig): 500 ng of total RNA were used for reverse transcription (RT) in a total reaction volume of 20 µL using the RevertAid H Minus Reverse Transcriptase (TSF) according to the manufacturer´s recommendations. An initial denaturing step was performed at 65°C for 5 min. The same RT primers as for strategy 1 were used to detect *leader*RNA+cRNA and cRNA only. Renilla mRNA+cRNA were reverse-transcribed with the RT primer luc (-): 5‘-AGA ACC ATT ACC AGA TTT GCC TGA-3’ hybridizing to the coding region of the Renilla luciferase reporter gene. cDNAs were diluted 1:10 and 2 µL of the dilution (~5 ng) were used in the respective PCR reaction; PCR reactions (final volume: 10 µL) further contained 1 pmol/µL each primer and 5 µL of the PowerUp SYBR Green Master Mix 2x (TFS); qPCR was performed in technical duplicates in a total volume of 10 µL on a QuantStudio3 Real-Time PCR System (TFS). PCR conditions were chosen according to the manufacturer’s fast cycling mode protocol: Uracil-DNA glycosylase (UDG) activation at 50°C for 2 min, initial denaturation of cDNA at 95°C for 2 min, followed by 40 cycles with denaturation at 95°C for 1 s, annealing and extension at 60°C for 30 s. RNA levels were quantified using the 2-ΔΔCT algorithm as described [4]. Primers for PCR amplification of *leader*RNA+cRNA and cRNA alone: RT_leader+cRNA and qPCR_leader+cRNA (sequences see above), yielding a product of 66 bp; note that specificity for cRNA was achieved by usage of RT primer RT_cRNA_s1/s2 that directed reverse transcription of positive strand RNA longer than ~120 nt and thus exceeding the maximal length of *leader*RNAs (~85 nt); primers for PCR amplification of Rluc mRNA+cRNA: luc (-) [see sequence above] and luc (+): 5‘-GGC CTC TTC TTA TTT ATG GCG A-3, yielding a product of 112 bp. Primer efficiencies were calculated according to [7] for the wt NP MG based on 3 independent experiments with 2 to 3 replicates, resulting in 2.00 ± 0.01 (standard deviation) for cRNA alone, 2.00 ± 0.02 for *leader*RNA+cRNA and 2.00 ±0.01 for mRNA+cRNA.

*Strategy 3*

(MG-transfected samples; S2C Fig): Reverse transcription was conducted as described for strategy 2 above and in [4]. RT primers were: luc (+) (see above) for the detection of negative-strand vRNA, primer luc (-) (see above) for RT of positive-stranded mRNA+cRNA, and primer RT_cRNA_s3: 5’-CAG TCC TGC CTT TTC TTT TAA TTT TAT C-3’ for specific detection of the cRNA trailer region. qPCR was conducted as for strategy 2; vRNA and mRNA+cRNA were amplified using primers luc (+) and luc (-). Specific amplification of cRNA was obtained with primers RT_cRNA s3 and qPCR_cRNA_s3: 5’- CGG TGA TAG CCT TAA TCT TTG TG-3’ (118 bp product).

In strategies 2 and 3, Firefly luciferase mRNA (encoded by the cotransfected pGL4.13 plasmid and used for normalization of transfection efficiency) was reverse-transcribed using a Random Hexamer Primer set (TFS), applying the same protocol as for the other RT primers utilized in strategies 2 and 3 (see above). PCR amplification was conducted with the validated primers [4] qPCR_FF_fwd: 5’-CGT GCA AAA GAA GCT ACC G-3’ and qPCR_FF_rev: 5’-GGT GGC AAA TGG GAA GTC AC-3’ (108 bp product) as described for strategy 2 above).

*Strategy 4*

(With standard curves, S2D Fig): qRT-PCR was performed as described for strategy 2; for the generation of standard curves, RT reactions were conducted with 0.5 pg/µL of the respective standard RNA; the cDNA samples were then subjected to serial dilutions (1:5 to 1:80,000) followed by qPCR (conditions as for strategy 2). Reverse transcription protocol: combine total RNA (final concentration 5 pg/μL), RT primer (final concentration 0.5 pmol/μL) and adjust the volume to 12.2 μL with ddH2O; heat up to 65°C for 5 min, followed by cooling to 25°C. Then add dNTPs to 1 nmol/μL each, adjust to 1 x RT buffer (50 mM Tris-HCl, 250 mM KCl, 4 mM MgCl2, 10 mM DTT, pH 8,3 bei 25°C), add 200 U RevertAid H minus RT (200 U/μL) and adjust the final volume to 20 µL with ddH2O if necessary. Incubate for 10 min at 25°C, 60 min at 42°C and 10 min at 70°C. The RT and qPCR primers were:

| **Name** | **sequence (5´🡪 3´)** |  |
| --- | --- | --- |
| RT_leader+cRNA | ATCTTCCTCATAGTTATTCGCACACA | RT and qPCR primer for the 65- and 73-mer |
| qPCR_leader + cRNA | GCCGGACACACAAAAAGAAAGAA | qPCR primer for 65-, 73- and 157-mer |
| RT_cRNA_s1/s2 | CAAACCAGGTGTGATTACAGTAACAATT | RT and qPCR primer for the 157-mer |
| RT_NP_mRNA | TTTTTTTTTTTTTTTTTTTTTTTTTTTTTTTTTTTCTTAATTATAAAACG | RT and qPCR primer for the 170-mer |
| qPCR_NP_mRNA | GTAACTCAATATTCTAACTAGCGATTTATCTAAATTAAATTACA | qPCR primer for the 170mer |
| RT_Rluc_3'-end | TTTTTTTTTTTTTTTTTTTTTTTTTTTTTATAATCTGTATGTTAATAACC | RT and qPCR Primer for the 181-mer |
| qPCR_Rluc_3'-end | TGAAATGGGAAAATATATCAAATCGTTCGTTGAG | qPCR primer for the 181-mer |

Interconversion of weight and molar concentration was performed at http://www.molbiol.ru/eng/scripts/01_07.html. The molecular weight of RNA molecules was calculated at https://www.aatbio.com/tools/calculate-RNA-molecular-weight-mw. The RNAs used for the generation of standard curves are summarized in S2 Table.

**Luciferase assays**

Luciferase assays were performed as described [4]. Cells were lysed in 200 μL 1× Reaction Lysis Buffer (2x Lysis-Juice; PJK). In case of RC monocistronic MG samples, lysates were further diluted 1:50 in ddH20 for Renilla Luciferase (Rluc) activity measurements. Undiluted lysates were used for Rluc measurements of RD monocistronic MG samples and bicistronic RC MG samples. Likewise, for FFLuc activity measurements of all MG variants, undiluted lysates were utilized. 10 μL of diluted or undiluted lysate were then mixed with either 50 μL of Rluc Reagent (Renilla-Juice Fluid; PJK) or 50 μL of FFluc Reagent (Beetle-Juice; PJK) and bioluminescene, as a measure of reporter gene activity, was detected using a Centro LB 960 luminometer (Berthold Technologies). In the case of monocistronic MGs (RC and RD), Renilla luciferase values were normalized to FFluc values to account for differences in transfection efficiency. Results obtained for monocistronic MGs carrying the native wt NP leader were set to 100%. Relative Renilla or Firefly activities of bicistronic MG samples were determined by normalization to the respective values obtained for the bici NP-NP construct (set to 100%).

**Northern blotting**

*Leader*RNA detection by Northern Blotting was performed as described [8] using 20-40 µg of total RNA extracted from EBOV-infected HuH7 cells or 10 ng of *in vitro* transcribed control RNAs (T7 transcripts covering the antigenomic EBOV leader sequences [5' to 3'] 2-78 and 56-158, as well as genomic leader nucleotides -154 to -1, as described in [9]. RNA samples were adjusted to 1x denaturing loading buffer containing 0.01% (w/v) bromophenol blue, 0.01 (w/v) xylene cyanol, 1.3 M urea, 33% (v/v) formamide and 1x TBE and were denatured for 3 min at 95°C, followed by rapid cooling on ice. Denatured RNAs were separated by native 20% polyacrylamide gel electrophoresis (PAGE) and afterwards transferred onto a positively charged nylon membrane (Roche) using overnight semidry electro-blotting and 0.5x TBE as transfer buffer. RNAs were crosslinked to the nylon membrane using 1-Ethyl-3-[3-dimethylaminopropyl]carbodiimide hydrochloride (EDC). Crosslinked membranes were transferred to a glass tube and preincubated with ~15 mL DIG Easy Hyb (Sigma Aldrich/Merck) hybridization solution for 2 h at 68°C. Afterwards, the pre-hybridization solution was exchanged with ~15 mL of hybridization solution containing 5 µL of the digoxigenin-labeled RNA probe nt 80-1 (identical in sequence to the 3'-terminal 80 nt of the genomic EBOV 3'-leader), followed by incubation overnight at 68°C. The probe was generated by *in vitro* T7 transcription using the DIG RNA labeling Kit (Sigma Aldrich/Merck) according to the manufacturer’s protocol. After hybridization, the membrane was washed twice with 50 mL stringency buffer 1 (2x SSC, saline sodium citrate; 0.1 % SDS) for 5 min each, followed by 2x washing with 50 mL stringency buffer 2 (0.1x SSC, 0.1% SDS) for 15 min each (20x SSC buffer was purchased from Sigma Aldrich/Merck). RNA was immunologically detected using the DIG northern starter Kit (Sigma Aldrich/Merck) following the manufacturer’s recommendations.

**qRT-PCR assay for innate immune induction**

To analyze the ability of the respective RNA to induce an innate immune response, 5 ×10^4^ HEK293 cells were seeded 24 h before stimulation with 250 ng of the respective RNA by transfection using the EndoFectin Max transfection reagent (BioCat, EF014-GC) according to the manufacturer’s instructions. Briefly, 1 µL of the respective RNAs (250 ng/µL) were mixed with 0.75 µL Endofectin and serum-free medium (OptiMEM, TFS, 31985070) to a final volume of 100 µL. In mock controls, the RNA was replaced by H2O. Furthermore, genomic Vesicular Stomatitis Virus (VSV) RNA, isolated as described [10], and yeast tRNA (Merck, 55714-250MG) were used as positive and negative RNA controls, respectively. Samples were mixed by gentle pipetting and incubated for 5 min at room temperature, followed by addition of the entire 100 µL to the cells. Cell transfection was performed at 37°C for 16 h at 5% CO2. Subsequently, the medium was removed and cells were gently washed once with PBS. Total cellular RNA was isolated using the RNeasy Mini Kit (Qiagen, 74106). A total of 100 ng of isolated RNA was subjected to cDNA synthesis using the PrimeScript RT Reagent Kit with gDNA Eraser (Takara, #RR047B), followed by qPCR employing the TB Green Premix Ex Taq (Tli RNase H Plus; Takara, #RR420B) and a StepOnePlus Real-Time PCR machine (Applied Biosystems). Human QuantiTect primers (Qiagen) were used to detect 18S ribosomal RNA (QT00199367), interferon-β (IFN-β) mRNA (QT00203763) and chemokine (C-X-C motif) ligand 10 mRNA (CXCL10 mRNA) (QT01003065); VSV RNA was detected using forward primer 5’-GAT AGT ACC GGA GGA TTG ACG ACT A-3’ and reverse primer 5’-TCA AAC CAT CCG AGC CAT TC-3’, and the EBOV leader RNAs using forward primer 5’-GCC GGA CAC ACA AAA AGA AAG AA-3’ and reverse primer 5’-ATC TTC CTC ATA GTT ATT CGC ACA CA-3’. CT-values were normalized against the corresponding 18S RNA value using the ΔΔCT method [11]. Graphs were generated by GraphPad Prism (version 7.03) and depicted as fold induction over mock, with mean values and standard deviations derived from six independent experiments.

**RNA-Seq: cDNA library preparations and Illumina Sequencing**

**A**) Isolation of total RNA from EBOV-infected HuH7 cells (experiment no. T1439, T1647, T1757) or from MG-transfected HEK293 cells (experiment no. T1484, T1647, T1757, T1842). Poly(A)+ RNA was isolated from total RNA. For libraries T1439 and T1484, mRNA was fragmented by ultrasound (2 pulses of 30 s at 4°C) before first-strand cDNA synthesis using N6 randomized primers. For construction of the other mentioned libraries, first-strand cDNA synthesis using N6 randomized primers was conducted first, followed by PCR in the presence of dUTP and uracil-specific DNA fragmentation [12] of DNA products. Then, the Illumina TruSeq sequencing adapters were ligated in a strand-specific manner to the 5'- and 3'-ends of the cDNA. Sample-specific barcodes were introduced by 5’ adapters only (T1439, T1484, T1647, T1757) or by both, 3’ and 5’ adapters (T1842). The cDNA was finally amplified with PCR (12 or 14 cycles) using a high fidelity DNA polymerase. For Illumina sequencing, the PCR products were purified (desalted) using the Agencourt AMPure XP kit (Beckman Coulter) and fractionated, that is, PCR products in the size range of 400 – 600 bp (exp. T1439, T1484), 350 - 700 bp (exp. T1647) or 300 - 500 bp (exp. T1842) were eluted from preparative agarose gels using the 'Nucleo Spin Gel and PCR Clean-up' Mini kit (Machery-Nagel).

**B**) Isolation of small RNA from EBOV-infected HuH7 or from MG-transfected HEK293 cells:

- exp. T 1439, EBOV-infected HuH7 cells: small RNAs (< 200 nt) were extracted and enriched using the mirVana miRNA Isolation Kit (with phenol; TFS) according to the manufacturer’s protocol;
- exp. T1757, small RNA fractions (< 200 nt) from EBOV-infected HuH7 cells were isolated from total RNA preparations using the RNeasy Plus Mini Kit (Qiagen, exp. T1757). Small RNAs (< 200 nt) from MG-transfected HEK293 cells were extracted and enriched using the mirVana miRNA Isolation Kit (see above);
- exp. T1842 and T2015, small RNAs (< 200 nt) from MG-transfected HEK293 cells were extracted and enriched using the mirVana miRNA Isolation Kit (see above).

For exp. T1842 and T2015, RNA samples were treated with CAP-Clip Acid Pyrophosphatase (T1842) or RppH (T2015) to generate 5'-monophosphate ends. Then rRNA was depleted using the Ribo-Zero rRNA Removal Kit GOLD for Human-Mouse-Rat (Illumina; T1842) or using an in-house protocol (vertis Biotechnologie AG). Oligonucleotide adapters were ligated to the 5'- and 3'-ends. Both, 3’ and 5’ adapters, harbored TruSeq barcode sequences that were specific for each sample. First-strand cDNA synthesis was performed using M-MLV reverse transcriptase and the 3’ adapter as primer. The resulting cDNAs were
PCR-amplified (12 cycles) using a high fidelity DNA polymerase. PCR products
were purified using the Agencourt AMPure XP kit (Beckman Coulter) and analyzed by
capillary electrophoresis. PCR product samples were pooled and size-fractionated to 140 – 270 bp by preparative agarose gel electrophoresis (see above). In the case of library T1842, fractionated DNAs were pooled with the poly(A) RNA-derived DNA pool of the same sequencing experiment [see (A) above]. Sequencing was performed on an Illumina NextSeq 500 system using 150 nt read length.

**C**) *1893 – spike-in control*

Small RNAs derived from non-infected/non-transfected HEK293 cells were prepared as described for exp- T1757 under B). Ribosomal RNA molecules were depleted using the Ribo-Zero rRNA Removal Kit GOLD for Human-Mouse-Rat (Illumina). 2% (referring to the RNA amount of the small RNA preparation) of a synthetic 65-mer *leader*RNA were added (spike-in) to the mock small RNA sample. Afterwards, CAP structures and 5’-triphosphate ends were removed using the CAP-Clip Acid Pyrophosphatase (Cellscript), and oligonucleotide adapters were ligated to the 5'- and 3'-ends. First-strand cDNA synthesis was performed using M-MLV reverse transcriptase and the 3’ adapter as primer. The resulting cDNA was PCR-amplified using a high fidelity DNA polymerase (12 cycles). The cDNA was purified using the Agencourt AMPure XP kit (Beckman Coulter Genomics), the combined length of the flanking sequences was 136 bp. The cDNA sample was size-fractionated in the size range of 160 – 500 bp using a preparative agarose gel. Sequencing was performed on an Illumina NextSeq 500 system using 75 nt read length.

*RNA-Seq Analysis*

RNA-Seq reads were trimmed using cutadapt [13] in three iterations removing the given 5’- and 3’-adapters and poly(A)-tails with a quality cutoff of 28. Libraries sequenced with two-color chemistry (NextSeq) typically contain reads with incorrect high-quality "G" calls at their 3’ end (no detected color encodes for  a "G"). These calls were removed using cutadapt's NextSeq-trimming option if the library was generated via NextSeq. Sequences shorter than 16 nt or with ≥10% unclear base identity ("N") were discarded. The trimmed sequences were mapped to the Human Transcriptome GRCh38 (NCBI, Genome Reference Consortium Human Build 38 patch release 13) and, specific for the sample, to the EBOV genome (NCBI, 1976 strain Mayinga / NC_002549.1) or to the respective MG variant using segemehl v0.2 [14] with an e-value threshold of 10-8. Ambiguously mapping reads were discarded.

sRNA and mRNA counts and statistics were derived using Perl-scripts. S3 Table gives an overview of all samples. A read was considered as a leader read if initiated complementary to nucleotides 1 to 3 of the EBOV genome 3'-end. Reads with their 5'-end mapping to the region between position 54 to 70 were included in transcription start site analyses (e.g. Fig 2B of the main text). Statistically relevant NP mRNA reads were derived from total RNA libraries enriched for poly(A)+ RNAs (see above) while leader reads were derived from small RNA libraries. Biological replicates were defined as RNA libraries derived from independent EBOV infection or MG transfection experiments. Figures were plotted using R [15].

**REFERENCES**

1. Hoenen T, Jung S, Herwig A, Groseth A, Becker S. Both matrix proteins of Ebola virus contribute to the regulation of viral genome replication and transcription. Virology. 2010; 403(1), 56–66. doi:10.1016/j.virol.2010.04.002. PMID: 20444481
2. Weik M, Modrof J, Klenk HD, Becker S, Mühlberger E. Ebola virus VP30-mediated transcription is regulated by RNA secondary structure formation. Journal of Virology. 2002; 76(17):8532–8539. doi: 10.1128/jvi.76.17.8532-8539.2002. PMID: 12163572
3. Bach S, Demper JC, Biedenkopf N, Becker S, Hartmann RK. RNA secondary structure at the transcription start site influences EBOV transcription initiation and replication in a length- and stability-dependent manner. RNA Biology. 2021; 18(4):523-536. doi: 10.1080/15476286.2020.1818459. PMID: 32882148
4. Bach S, Biedenkopf N, Grünweller A, Becker S, Hartmann RK. Hexamer phasing governs transcription initiation in the 3'-leader of Ebola virus. RNA. 2020; 26(4):439-453. doi: 10.1261/rna.073718.119. PMID: 31924730
5. Brauburger K, Boehmann Y, Krähling V, Mühlberger E. Transcriptional Regulation in Ebola Virus: Effects of Gene Border Structure and Regulatory Elements on Gene Expression and Polymerase Scanning Behavior. Journal of Virology. 2015; 90(4): 1898–1909. doi: 10.1128/JVI.02341-15. PMID: 26656691
6. Bach S, Demper JC, Grünweller A, Becker S, Biedenkopf N, Hartmann RK. Regulation of VP30-dependent transcription by RNA sequence and structure in the genomic Ebola virus promoter. Journal of Virology. 2020; 95(5):e02215-20. doi: 10.1128/JVI.02215-20. PMID: 33268520
7. Ramakers C, Ruijter JM, Lekanne Deprez RH, Moorman AF. Assumption-free analysis of quantitative real-time polymerase chain reaction (PCR) data. Neuroscience Letters. 2003; 339(1):62–66. doi:10.1016/S0304-3940(02)01423-4. PMID: 12618301
8. Hoch PG, Schlereth J, Lechner M, Hartmann RK (2016). *Bacillus subtilis* 6S-2 RNA serves as a template for short transcripts *in vivo*. RNA. 2016; 22(4): 614–622. doi: 10.1261/rna.055616.115. PMID: 26873600
9. Schlereth J, Grünweller A, Biedenkopf N, Becker S, Hartmann RK. RNA binding specificity of Ebola virus transcription factor VP30. RNA Biology. 2016; 13(9):783–798. doi:10.1080/15476286.2016.1194160. PMID: 27315567
10. Habjan M, Andersson I, Klingström J, Schümann M, Martin A, Zimmermann P, et al. Processing of genome 5' termini as a strategy of negative-strand RNA viruses to avoid RIG-I-dependent interferon induction. PLoS One; 2008; 3(4):e2032.
    doi: 10.1371/journal.pone.0002032. PMID: 18446221
11. Livak KJ, Schmittgen TD. Analysis of relative gene expression data using real-time quantitative PCR and the 2(-Delta Delta C(T)) Method. Methods. 2001; 25(4):402-408.
    doi: 10.1006/meth.2001.1262. PMID: 11846609
12. Müller KM, Stebel SC, Knall S, Zipf G, Bernauer HS, Arndt KM. Nucleotide exchange and excision technology (NExT) DNA shuffling: a robust method for DNA fragmentation and directed evolution. Nucleic Acids Research. 2005; 33(13): e117. doi: 10.1093/nar/gni116. PMID: 16061932
13. Martin M. Cutadapt removes adapter sequences from high-throughput sequencing reads. EMBnet.journal. 2011; 17(1):10. doi:10.14806/ej.17.1.200.
14. Hoffmann S, Otto C, Kurtz S, Sharma CM, Khaitovich P, Vogel J, et al. Fast mapping of short sequences with mismatches, insertions and deletions using index structures. PLoS Computational Biology. 2009; 5(9): e1000502. doi:10.1371/journal.pcbi.1000502. PMID: 19750212
15. The R Development Core Team. R: A Language and Environment for Statistical Computing Reference Index. R Foundation for Statistical Computing Available at: https://stat.ethz.ch/pipermail/r-help/2008-May/161481.html [Accessed November 29, 2019].
