# Supplementary material for: Identification and characterization of short leader and trailer RNAs synthesized by the Ebola virus RNA polymerase
Source: PLoS Pathog. 2021 Oct 26;17(10):e1010002. doi: 10.1371/journal.ppat.1010002 (PMC8547711; doi:10.1371/journal.ppat.1010002)
Supplement: S1 Table — (DOCX) [file ppat.1010002.s002.docx]

**S1 Table:** Primers and DNA fragments used in this study for the construction of mutant minigenomes.

| **no.** | **name** | **cloning**  **strategy** | | **primer fwd** | **primer sequence**  **fwd (5‘ to 3‘)** | **primer rev** | **primer sequence**  **rev (5‘ to 3‘)** |
| --- | --- | --- | --- | --- | --- | --- | --- |
| **Inside-out primer deletion mutagenesis (Fig. S1 A)** | | | | | | | |
| 1 | p3E5E_3’-Δ1 | mutagenesis of p3E5E_Rluc_RC | | pS_246 | GGGTCGGCATGGCATCTCC | pS_245 | GGACACACAAAAAGAAAGAAGAATTTTTAGGATCTTTTGTG |
| 2 | p3E5E_3’-Δ2 | mutagenesis of p3E5E_Rluc_RC | | pS_246 | GGGTCGGCATGGCATCTCC | pS_247 | GACACACAAAAAGAAAGAAGAATTTTTAGGATCTTTTGTGTG |
| 3 | RD_p3E5E_  3’-Δ1 | mutagenesis of p3E5E_Rluc_RD | | pS_246 | GGGTCGGCATGGCATCTCC | pS_245 | GGACACACAAAAAGAAAGAAGAATTTTTAGGATCTTTTGTG |
| 4 | RD_p3E5E_  3’-Δ2 | mutagenesis of p3E5E_Rluc_RD | | pS_246 | GGGTCGGCATGGCATCTCC | pS_247 | GACACACAAAAAGAAAGAAGAATTTTTAGGATCTTTTGTGTG |
| **Restriction cloning** | | | | | | | |
|  |  | |  |  |  |  |  |
| 5 | pA-3E5E-Gluc | | 1. Backbone -PCR pANDY RLuc to introduce restriction sites NdeI and NotI |  | GCAGCA*CATATG*GGTGGTATGAGACATA |  | GCAGCA*TCTAGA*GCGGCCGCATAG |
|  |  |  | 1. Insert: Gaussia Luciferase Gluc, amplified with primers containing NotI and NdeI restriction sites   from template pT7RiboSM2_vS_Gluc [1] |  | GCAGCA*GCGGCCGC* TTAGTCACCAC |  | GCAGCA*CATATG*GA GTCAAAGTTC |
|  |  |  | Restriction digestion of a) and b) with NotI (5’-GCGGCCGC) and NdeI (5'-CATATG) followed by T4 DNA ligase ligation | | | | |
| 6 | pA-3E5E-GLuc-GFP | | 1. Backbone pA-3E5E-Gluc |  |  |  |  |
|  |  |  | 1. Insert GeneArt DNA string fragment EBOV_IR_NP VP35-GFP | GeneArt Strings DNA fragment_EBOV IR NP VP35-GFP: GCAGCA***GCGGCCGC***ATGAGCATGGAACAATGGGATGATTCAACCGACAAA  TAGCTAACATTAAGTAGTCAAGGAACGAAAACAGGAAGAATTTTTGATGT  CTAAGGTGTGAATTATTATCACAATAAAAGTGATTCTTATTTTTGAATTT  AAAGCTAGCTTATTATTACTAGCCGTTTTTCAAAGTTCAATTTGAGTCTT  AATGCAAATAGGCGTTAAGCCACAGTTATAGCCATAATTGTAACTCAATA  TTCTAACTAGCGATTTATCTAAATTAAATTACATTATGCTTTTATAACTT  ACCTACTAGCCTGCCCAACATTTACACGATCGTTTTATAATTAAGAAAAA  ACTAATGATGAAGATTAAAACCTTCATCATCCTTACGTCAATTGAATTCT  CTAGCACTCGAAGCTTATTGTCTTCAATGTAAAAGAAAAGCTGGTCTAAC  AAGATGCTCGAGATGGTGAGCAAGGGCGAGGAGCTGTTCACCGGGGTGGT  GCCCATCCTGGTCGAGCTGGACGGCGACGTAAACGGCCACAAGTTCAGCG  TGTCCGGCGAGGGCGAGGGCGATGCCACCTACGGCAAGCTGACCCTGAAGTTCATCTGCACCACCGGCAAGCTGCCCGTGCCCTGGCCCACCCTCGTGACCACCCTGACCTACGGCGTGCAGTGCTTCAGCCGCTACCCCGACCACATGAAGCAGCACGACTTCTTCAAGTCCGCCATGCCCGAAGGCTACGTCCAGGAGCGCACCATCTTCTTCAAGGACGACGGCAACTACAAGACCCGCGCCGAGGTGAAGTTCGAGGGCGACACCCTGGTGAACCGCATCGAGCTGAAGGGCATCGACTTCAAGGAGGACGGCAACATCCTGGGGCACAAGCTGGAGTACAACTACAACAGCCACAACGTCTATATCATGGCCGACAAGCAGAAGAACGGCATCAA  GGTGAACTTCAAGATCCGCCACAACATCGAGGACGGCAGCGTGCAGCTCG  CCGACCACTACCAGCAGAACACCCCCATCGGCGACGGCCCCGTGCTGCTG  CCCGACAACCACTACCTGAGCACCCAGTCCGCCCTGAGCAAAGACCCCAA  CGAGAAGCGCGATCACATGGTCCTGCTGGAGTTCGTGACCGCCGCCGGGA  TCACTCTCGGCATGGACGAGCTGTACAAGTGA***GCGGCCGC***GCAGCA | | | |
|  |  |  | Restriction digestion of c) and d) with NotI (5’- GCGGCCGC -3’) followed by T4 DNA ligase ligation. | | | | |
| 7 | bici NP-VP35 | | **5-step cloning:**  1) Backbone-PCR  template: pA-3E5E-GLuc-GFP | 16 | TTT***TTAATTAA***ATAGTATCCTGATACTTGCAAAGGTTGG | 17 | TTT***CTCGAG***CTTGTTAGACCAGCTTTTCTTTTACATTG |
|  |  |  | 2) PCR to exchange GFP with Firefly Luc, template: pCAGGS_Luc2 [2] | 18 | TTT***CTCGAG***ATGGAAGATGCCAAAAACATTAAGAAGG | 19 | TTT***TTAATTAA***TTACACGGCGATCTTGCCG |
|  |  |  | 3) Restriction digestion of 1) and 2) with PacI (5’- TTAATTAA -3’) and XhoI (5’-CTCGAG -3’) followed by T4 DNA ligase ligation. | | | | |
|  |  |  | 4) PCR to introduce NotI and NdeI restriction sites; template: p3E5E_Rluc_RC | 20 | TTT***GCGGCCGC***TTATTGTTCATTTTTGAGAACTCGCTC | 21 | TTT***CATATG***ATGACTTCGAAAGTTTATGATCCAG |
|  |  |  | 5) Restriction digestion of 3) and 4) with NotI (5’- GCGGCCGC -3’) and NdeI (5’- CATATG -3’) followed by T4 DNA ligase ligation to exchange Gaussia Luc (GLuc) with Renilla Luc (RLuc). | | | | |
| 8 | bici NP-NP | | template backbone: bici NP-VP35; template insert: GeneArt Strings DNA fragment_NP | GeneArt Strings DNA fragment_NP: TTC***CTCGAG***CTTGTTAGACCAGCTTTTCTTTTACATTGAAGACAATAAGCTTCGAGTGCTAGAGAATTCAATTGACGTAAGGATGAGGAAAATTATTAATCTTCCTCATTAGTTTTTTCTTAATTATAAAACGATCGTGTAAATGTTGGGCAGGCTAGTAGGTAAGTTATAAAAGCATAATGTAATTTAATTTAGATAAATCGCTAGTTAGAATATTGAGTTACAATTATGGCTATAACTGTGGCTTAACGCCTATTTGCATTAAGACTCAAATTGAACTTTGAAAAACGGCTAGTAATAATAAGCTAGCTTTAAATTCAAAAATAAGAATCACTTTTATTGTGATAATAATTCACACCTTAGACATCAAAAATTCTTCCTGTTTTCGTTCCTTGACTACTTAATGTTAGCTATTTGTCGGTTGAATCATCCCATTGTTCCATGCTCAT***GCGGCCGC***GAG | | | |
|  |  | | Restriction digestion with XhoI (5’- CTCGAG -3’) and NotI (5’- GCGGCCGC -3’) followed by T4 DNA ligase ligation. | | | | |
| 9 | bici NP-VP40 | | template backbone: bici NP-VP35; template insert: GeneArt Strings DNA fragment _VP40 | GeneArt Strings DNA fragment _VP40: TTC***CTCGAG***CTTGTTAGACCAGCTTTTCTTTTACATTGAAGACAATAAGCTTCGAGTGCTAGAGAATTCAATTGACGTAAGGATGATGAAGGTTAATGAAAAAACACTCTCTCAGCCGAGGTAGGTTTTTCTTAATCTTCATCATTAGTTTTTTCTTAATTATAAAACGATCGTGTAAATGTTGGGCAGGCTAGTAGGTAAGTTATAAAAGCATAATGTAATTTAATTTAGATAAATCGCTAGTTAGAATATTGAGTTACAATTATGGCTATAACTGTGGCTTAACGCCTATTTGCATTAAGACTCAAATTGAACTTTGAAAAACGGCTAGTAATAATAAGCTAGCTTTAAATTCAAAAATAAGAATCACTTTTATTGTGATAATAATTCACACCTTAGACATCAAAAATTCTTCCTGTTTTCGTTCCTTGACTACTTAATGTTAGCTATTTGTCGGTTGAATCATCCCATTGTTCCATGCTCAT***GCGGCCGC***GAG | | | |
|  |  | | Restriction digestion with XhoI and NotI followed by T4 DNA ligase ligation. | | | | |
| **Complementary primer mutagenesis (S1C Fig)** | | | | | | | |
| 10 | bici NP GEmut-NP | | mutagenesis of bici NP-NP | pS_175 | CTTCCTCATTAGT**A**T**A**T**A**CTTAATTATAAAACGATCG | pS_176 | CGATCGTTTTATAATTAAG**T**A**T**A**T**ACTAATGAGGAAG |
| 11 | bici NP GEmut-VP40 | | mutagenesis of bici NP-VP40 | pS_173 | CTTCATCATTAGT**A**T**A**T**A**CTTAATTATAAAACGATCGTG | pS_174 | CACGATCGTTTTATAATTAAG**T**A**T**A**T**ACTAATGATGAAG |
| 12 | p3E5E_3' +1 | | mutagenesis of p3E5E_Rluc_RC | pS_248 | CTTCTTTCTTTTTGTGTGTCCG**C**GGGTCGGCATGGCATC | pS_249 | GATGCCATGCCGACCC**G**CGGACACACAAAAAGAAAGAAG |
| 13 | RD_p3E5E_  3' +1 | | mutagenesis of p3E5E_Rluc_RD | pS_248 | CTTCTTTCTTTTTGTGTGTCCG**C**GGGTCGGCATGGCATC | pS_249 | GATGCCATGCCGACCC**G**CGGACACACAAAAAGAAAGAAG |
| **Overhang/inside-out primer mutagenesis (S1B Fig)** | | | | | | | |
| 14 | bici NP GEmut-*le*NP | | mutagenesis of bici NPGEmut-NP | pS_189 | **CTAAAAATTCTTCTTTCTTTTTGTGTGTCCG**ATTAGTATATACTTAATTATAAAACGATCGTGTAAATG | pS_190 | **GATCTTTTGTGTGCGAATAACTAT**GAGGAAGATTAATAATTTTCCTCATCCTTAC |

The different PCR-based strategies for site-directed mutagenesis are illustrated in S1 Fig (except for the classical restriction cloning). The EBOV wt minigenome pANDY 3E5E is termed p3E5E_Rluc_RC; RC indicates the replication-competent and RD replication-deficient minigenomes. The EBOV wt bicistronic RC minigenome pA-3E5E-RLuc-FFLuc is termed bici_NP-VP35. Hyphens in primer sequences mark nucleotide deletions, boldface underlined nucleotides substitutions or insertions. In case of restriction cloning underlined nucleotides mark overhangs and boldface italic nucleotides mark restriction sites.

**References**

1. Devignot S, Bergeron E, Nichol S, Mirazimi A, Weber F. A virus-like particle system identifies the endonuclease domain of Crimean-Congo hemorrhagic fever virus. J Virol. 2015; 89(11):5957-5967. doi: 10.1128/JVI.03691-14.
2. Biedenkopf N, Hoenen T. Modeling the Ebolavirus Life Cycle with Transcription and Replication-Competent Viruslike Particle Assays. Methods Mol Biol. 2017; 1628:119–131. doi:10.1007/978-1-4939-7116-9_9.
