# Supplementary material for: Identification and characterization of short leader and trailer RNAs synthesized by the Ebola virus RNA polymerase
Source: PLoS Pathog. 2021 Oct 26;17(10):e1010002. doi: 10.1371/journal.ppat.1010002 (PMC8547711; doi:10.1371/journal.ppat.1010002)

S2 Table: Synthetic RNA oligonucleotides (1, 2, 3a, 3b) and T7 transcripts (4-6)

no.	name	sequence (5‘→3‘)	source
1	5’-PPP-*leader*RNA, 65-mer	5'-pppCGG ACA CAC AAA AAG AAA GAA GAA UUU UUA GGA UCU UUU GUG UGC GAA UAA CUA UGA GGA AGA UU	AXOlabs
2	5'-PPP-*leader*RNA, 73-mer	5'-pppGGA CAC ACA AAA AGA AAG AAG AAU UUU UAG GAU CUU UUG UGU GCG AAU AAC UAU GAG GAA GAU UAA UAA UUU U-3'	AXOlabs
3a	Oligo 3P-G	5'-pppGAC ACA CAC ACA CAC ACA CAC UUU-3’	AXOlabs
3b	Oligo AS G24	5'-OH-AAA GUG UGU GUG UGU GUG UGU GUC-3’	AXOlabs
4	(+) RNA 2-158, (157-mer, T7 transcript)	5‘-GGACACACAAAAAGAAAGAAGAAUUUUUAGGAUCUUUUGU GUGCGAAUAACUAUGAGGAAGAUUAAUAAUUUUCCUCUCA UUGAAAUUUAUAUCGGAAUUUAAAUUGAAAUUGUUACUGU AAUCACACCUGGUUUGUUUCAGAGCCACAUCACAAAG	T7 transcript
5	NP_mRNA (170-mer, T7 transcript)	5‘-GGCGUUAAGCCACAGUUAUAGCCAUAAUUGUAACUCAAU AUUCUAACUAGCGAUUUAUCUAAAUUAAAUUACAUUAUGCU UUUAUAACUUACCUACUAGCCUGCCCAACAUUUACACGAUC GUUUUAUAAUUAAGAAAAAAAAAAAAAAAAAAAAAAAAAAAAA AAAAAA	T7 transcript
6	Rluc_mRNA (181-mer, T7 transcript)	5‘-GGUCUUCAUUUUUCGCAAGAAGAUGCACCUGAUGAAAUG GGAAAAUAUAUCAAAUCGUUCGUUGAGCGAGUUCUCAAAAA UGAACAAUAAUUCUAGAGCGGCCGCAUAGUAUCCUGAUACU UGCAAAGGUUGGUUAUUAACAUACAGAUUAUAAAAAAAAAAA AAAAAAAAAAAAAAAAAA	T7 transcript

The predicted secondary structures for the synthetic RNA oligonucleotides are shown underneath the table. For the construction of T7 expression plasmids and preparation of the 157-, 170- and 181-meric RNAs used as standards in qRT-PCR, see paragraph “**qRT-PCR**“, subparagraph “**Strategy 4**“ below. The duplex formed by annealing of RNA oligonucleotides 3a and 3b was used in the innate immunity analysis presented in Fig 11 of the main text.

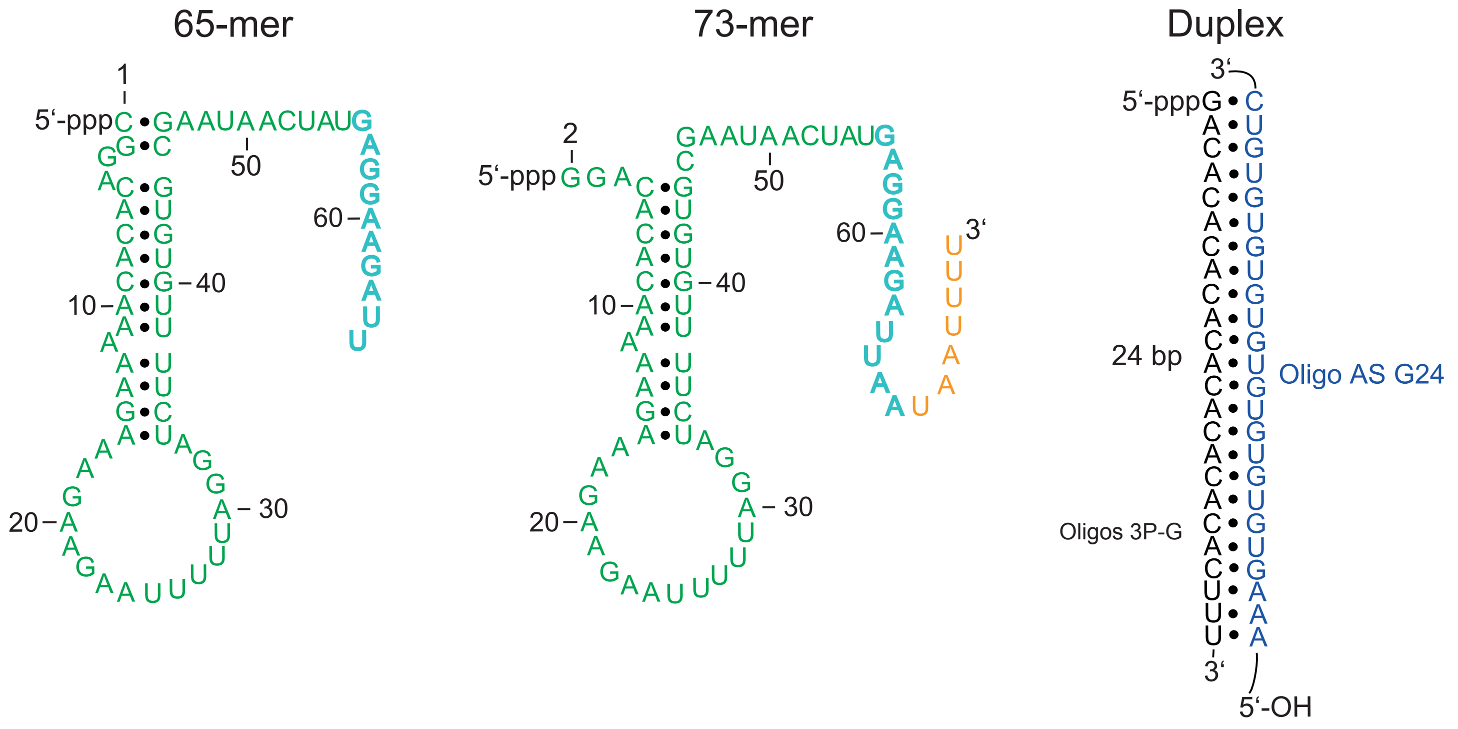

Supplement: S2 Table — (PDF) [file ppat.1010002.s003.pdf]
