# Supplementary material for: Identification and characterization of short leader and trailer RNAs synthesized by the Ebola virus RNA polymerase
Source: PLoS Pathog. 2021 Oct 26;17(10):e1010002. doi: 10.1371/journal.ppat.1010002 (PMC8547711; doi:10.1371/journal.ppat.1010002)
Supplement: S1 Fig — (A) Inside-out primer deletion mutagenesis. (B) Overhang/inside-out primer insertion mutagenesis. (C) Complementary primer mutagenesis for introduction of insertions or substitutions. In approaches A and B the entire plasmid is amplified with 5‘-phosphorylated primers that introduce the desired insertions/deletions, followed by circularization of PCR products and template removal by Dpn I treatment before bacterial transformation. In approach C phosphorylation of 5’-ends and ligation are carried out by bacterial enzymes after DNA transformation. (DOCX) [file ppat.1010002.s006.docx]

**S1 Fig.** PCR-based strategies (essentially performed as previously described, [1]) that were used for the construction of mutant minigenomes. (**A**) Inside-out primer deletion mutagenesis. (**B**) Overhang/inside-out primer insertion mutagenesis. (**C**) Complementary primer mutagenesis for introduction of insertions or substitutions. In approaches A and B the entire plasmid is amplified with 5‘-phosphorylated primers that introduce the desired insertions/deletions, followed by circularization of PCR products and template removal by Dpn I treatment before bacterial transformation. In approach C phosphorylation of 5'-ends and ligation are carried out by bacterial enzymes after DNA transformation.

**Reference:**

1. Bach S, Biedenkopf N, Grünweller A, Becker S, Hartmann RK. Hexamer phasing governs transcription initiation in the 3'-leader of Ebola virus. RNA. 2020; 26(4):439-453. doi: 10.1261/rna.073718.119. PMID: 31924730
