# Supplementary material for: Identification and characterization of short leader and trailer RNAs synthesized by the Ebola virus RNA polymerase
Source: PLoS Pathog. 2021 Oct 26;17(10):e1010002. doi: 10.1371/journal.ppat.1010002 (PMC8547711; doi:10.1371/journal.ppat.1010002)
Supplement: S2 Fig — (DOCX) [file ppat.1010002.s007.docx]

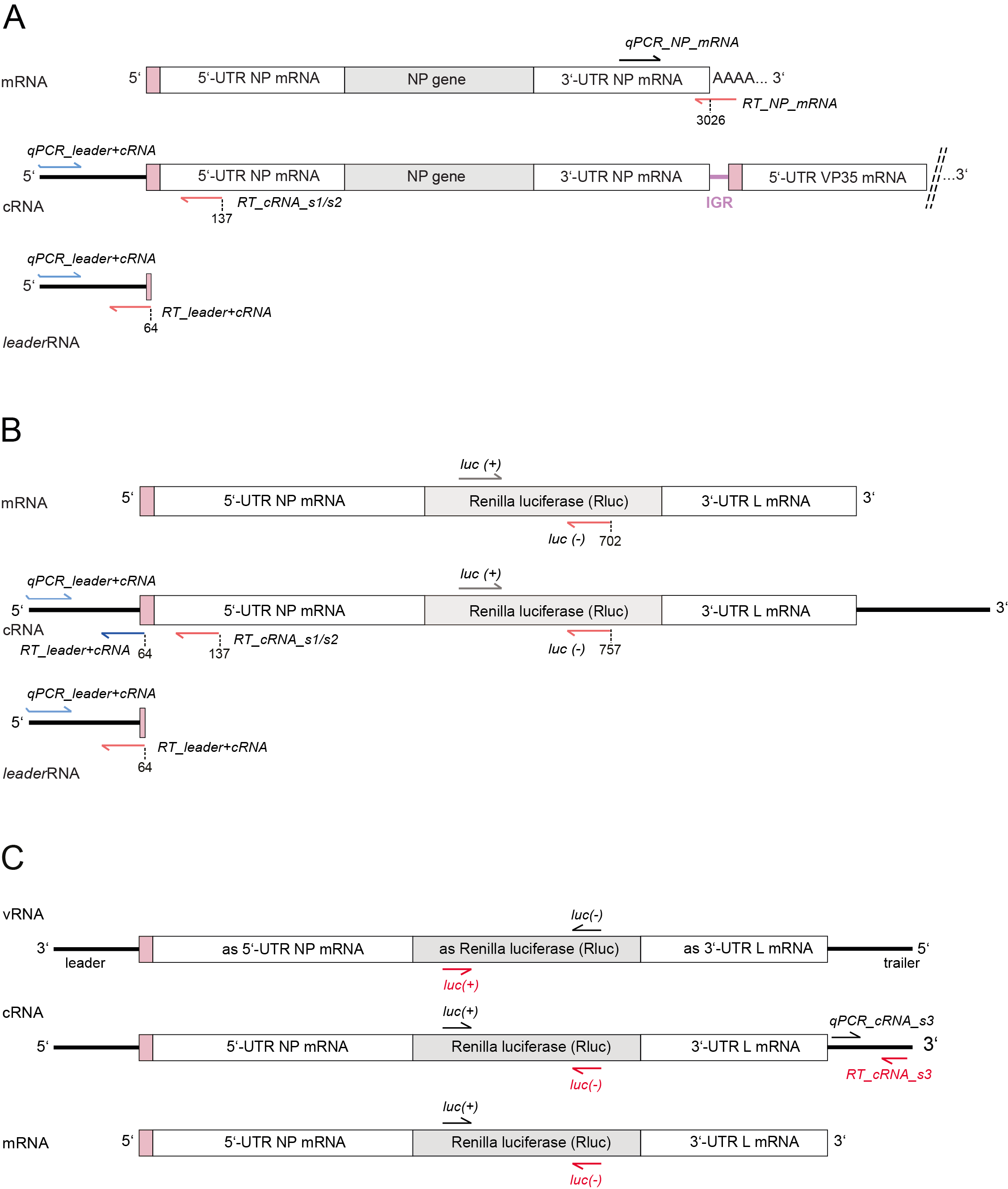


**
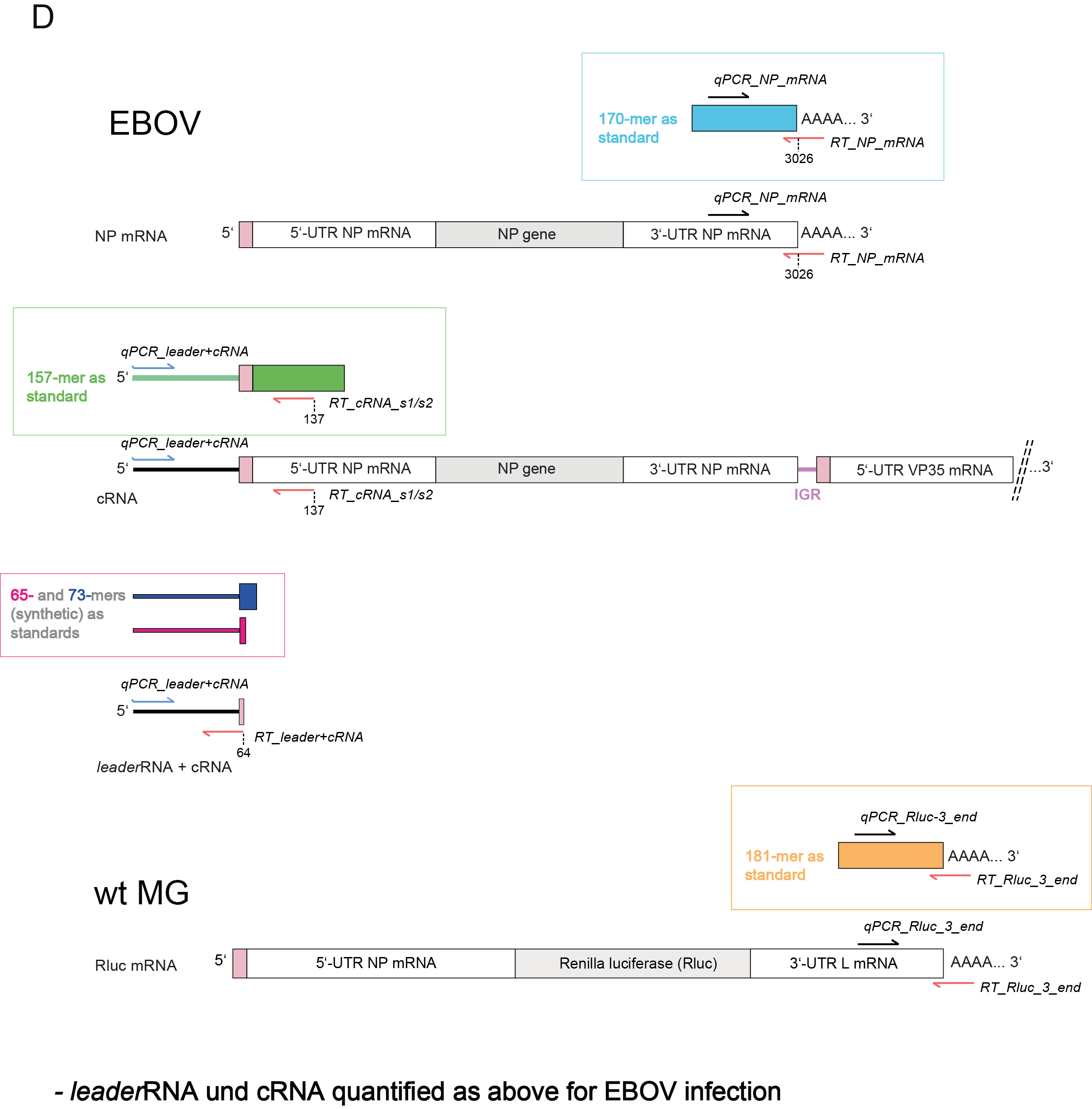
**

**S2 Fig. qRT-PCR strategies applied to the quantification of viral RNA species. (A)** RT-PCR strategy 1 for the detection of viral RNAs in total RNA preparations from EBOV-infected HuH7 cells. (**B**) RT-PCR strategy 2 for the detection of viral RNAs, including *leader*RNAs, in total RNA preparations from MG-transfected HEK293 cells. A peculiarity of this strategy was that the RT primer (RT_cRNA_s1/s2) was not included in the qPCR reaction; instead, primers RT_leader+cRNA and qPCR_leader+cRNA were used, resulting in the same PCR product (66 bp) and thus equalizing primer efficiencies for cRNA and *leader*RNA+cRNA PCR. The primer pair for the Rluc reporter gene was previously demonstrated to enable reliable quantification of mRNA and cRNA [1,2]. (**C**) RT-PCR strategy 3 for the detection of viral RNAs (vRNA, mRNA, cRNA) in total RNA preparations from MG-transfected HEK293 cells. Red arrows indicate reverse transcription (RT) primers, with their 5’-ends indicated by dashed vertical lines and nt position. PCR primers utilized for *leader*RNA amplification are depicted in blue. Pink areas mark the 5'-terminal RNA hairpin structures of viral mRNAs; IGR, intergenic region; vRNA, negative sense genomic RNA; cRNA, complementary positive sense antigenomic RNA. (**D**) RT-PCR concept (strategy 4) for the quantification of molar ratios of NP mRNA to *leader*RNA in EBOV-infected (top) based on qRT-PCR standard curves. The standard RNAs included the 170-mer (a T7 transcript covering the junction between NP mRNA and poly(A) tail), the 157-mer (a T7 transcript covering the 5'-terminal region of antigenomic cRNA) and synthetic 65- and 73-meric *leader*RNA mimics. For the quantification of molar ratios of Rluc mRNA to *leader*RNA in MG-transfected cells (bottom), a 181-meric T7 transcript covering the junction between Rluc mRNA and its poly(A) tail) was used for generating the standard curve; leaderRNA and cRNA were quantified as for the EBOV infection model above.

**References:**

1. Hoenen T, Jung S, Herwig A, Groseth A, Becker S. Both matrix proteins of Ebola virus contribute to the regulation of viral genome replication and transcription. Virology. 2010; 403(1), 56–66. doi:10.1016/j.virol.2010.04.002. PMID: 20444481
2. Bach S, Biedenkopf N, Grünweller A, Becker S, Hartmann RK. Hexamer phasing governs transcription initiation in the 3'-leader of Ebola virus. RNA. 2020; 26(4):439-453. doi: 10.1261/rna.073718.119. PMID: 31924730
