# Supplementary material for: Identification and characterization of short leader and trailer RNAs synthesized by the Ebola virus RNA polymerase
Source: PLoS Pathog. 2021 Oct 26;17(10):e1010002. doi: 10.1371/journal.ppat.1010002 (PMC8547711; doi:10.1371/journal.ppat.1010002)
Supplement: S3 Fig — (DOCX) [file ppat.1010002.s008.docx]

**
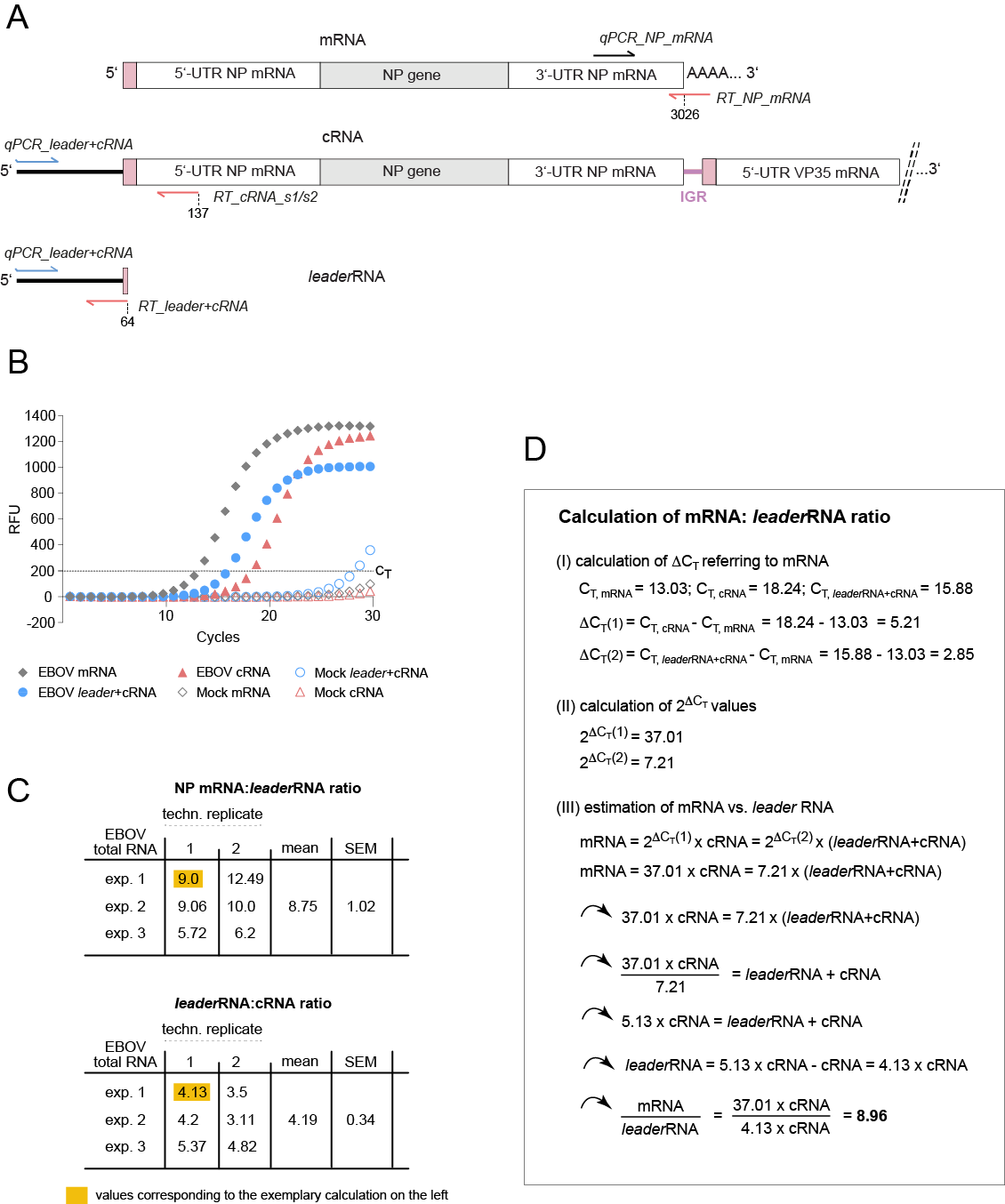
**

**S3 Fig.** Mathematical approach to estimate mRNA:*leader*RNA ratios in EBOV-infected HuH7 cells. (**A**) Applied RT-PCR strategy (identical to S2A Fig). (**B**) Exemplary qPCR amplification plot obtained by the three different primer sets for NP mRNA, *leader*RNA+cRNA or cRNA. Y-axis: relative fluorescence signal (RFU) per PCR cycle; Mock = non-infected cells. (**C**) Quantification of the NP mRNA:*leader*RNA and *leader*RNA:cRNA ratios for 3 biological replicates with 2 technical replicates each and the resulting mean ratio ± SEM. (**D**) An exemplary calculation for one technical replicate (marked in yellow in panel C), based on ΔCT values for mRNA, cRNA and *leader*RNA+cRNA.
