# Supplementary material for: Identification and characterization of short leader and trailer RNAs synthesized by the Ebola virus RNA polymerase
Source: PLoS Pathog. 2021 Oct 26;17(10):e1010002. doi: 10.1371/journal.ppat.1010002 (PMC8547711; doi:10.1371/journal.ppat.1010002)
Supplement: S4 Fig — (A) qRT-PCR standard curves for the individual standard RNAs. The molar amount of the respective RNA fragment introduced into the qRT-PCR reaction is given on the x-axis in logarithmic scale. The equation of the linearized calibration curve is shown as inset. (B) RT-PCR products using total RNA from EBOV-infected or MG-transfected cells and the different primer pairs according to strategy 4 were analyzed on a 2% agarose gel; the PCR products obtained after 40 PCR cycles were stained with GelRed; M, 100 bp ladder (CytoGen GmbH, Sinn, Germany) used as size marker. (C) Example calculations of NP mRNA:leaderRNA amounts in EBOV-infected cells based on the standard curve for the 73-meric leaderRNA mimic, and Rluc mRNA:leaderRNA amounts in MG-transfected cells based on the standard curve for the 65-meric leaderRNA mimic. (PDF) [file ppat.1010002.s009.pdf]

A

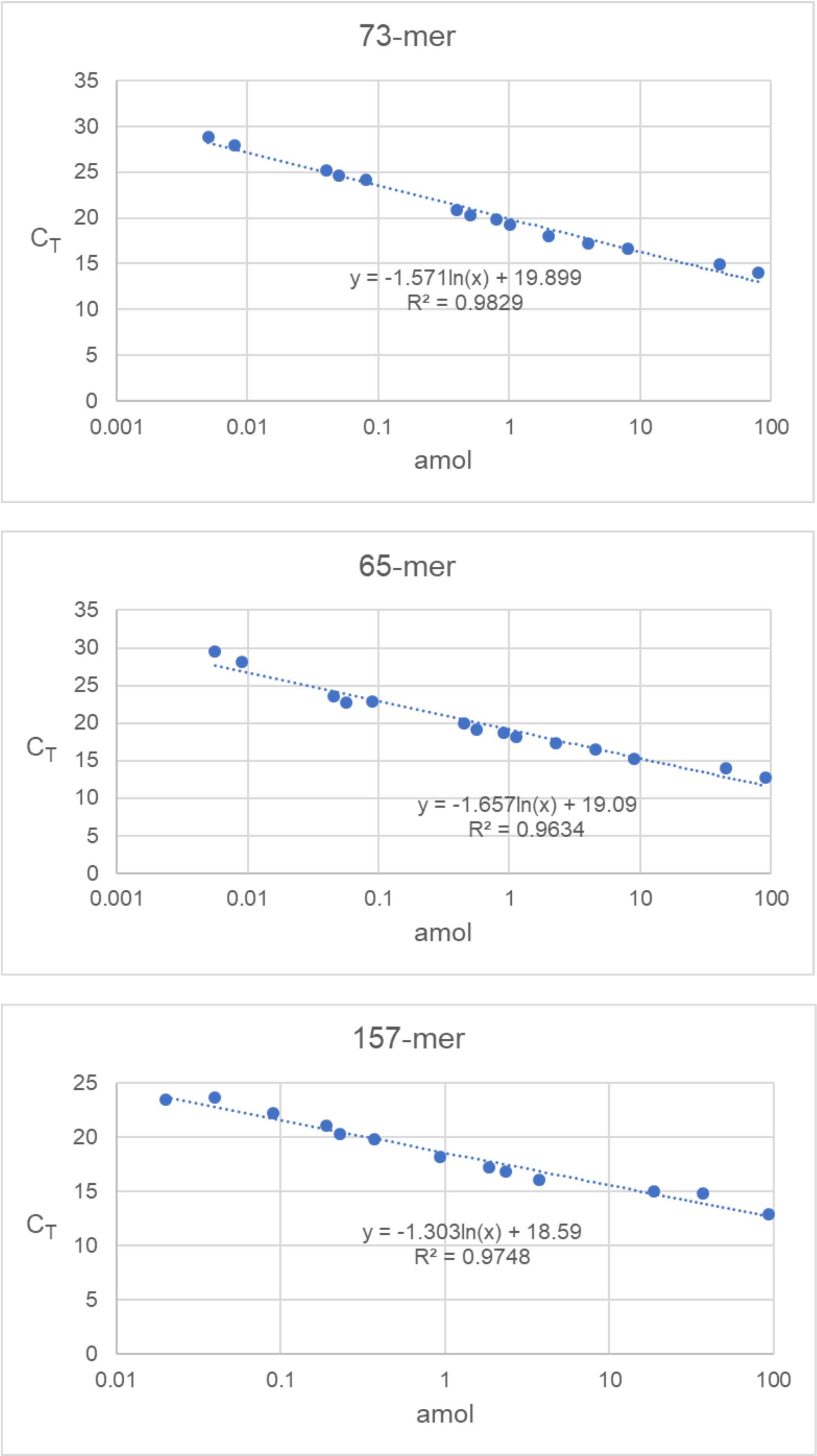

73-mer
35
30
25
20
15
10
5
0
CT
0.001
0.01
0.1
1
10
100
amol
y = -1.571ln(x) + 19.899
R² = 0.9829
65-mer
35
30
25
20
15
10
5
0
CT
0.001
0.01
0.1
1
10
100
amol
y = -1.657ln(x) + 19.09
R² = 0.9634
157-mer
25
20
15
10
5
0
CT
0.01
0.1
1
10
100
amol
y = -1.303ln(x) + 18.59
R² = 0.9748

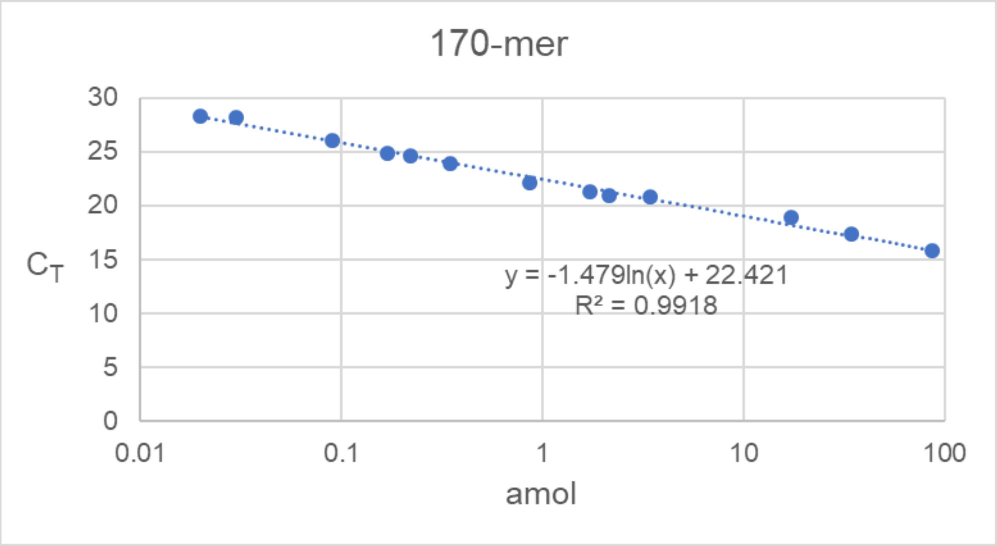
170-mer
30
25
20
15
10
5
0
C_T
0.01
0.1
1
10
100
amol
y = -1.479ln(x) + 22.421
R² = 0.9918

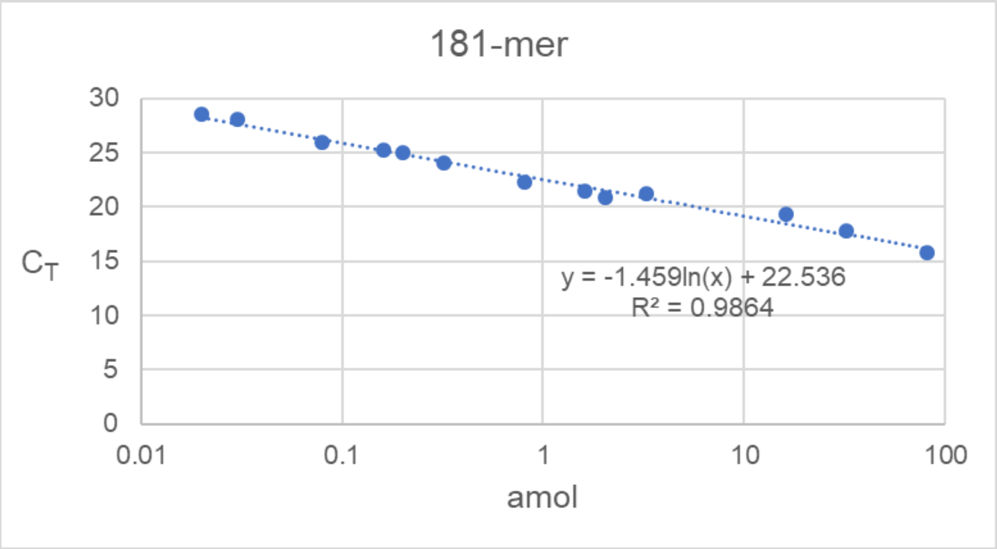
181-mer
30
25
20
15
10
5
0
C_T
0.01
0.1
1
10
100
amol
y = -1.459ln(x) + 22.536
R² = 0.9864

B

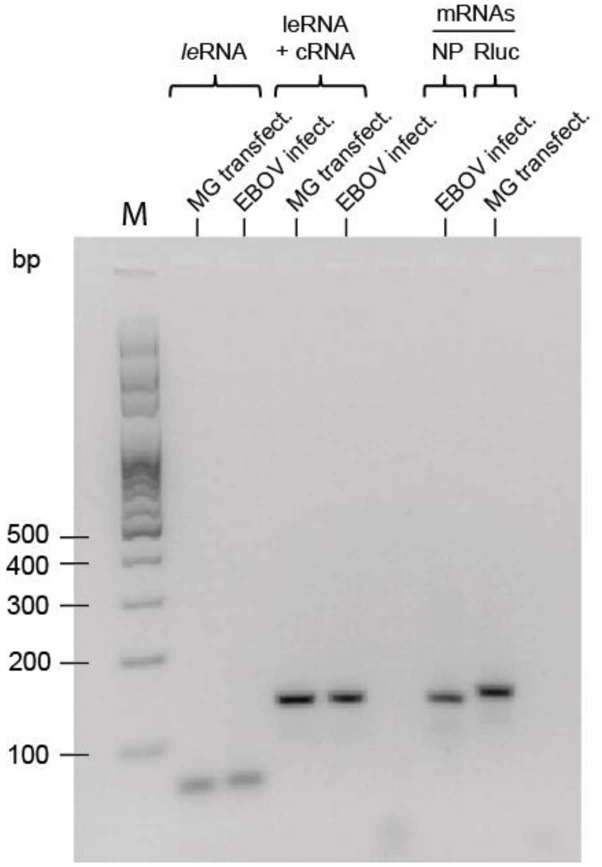

leRNA
+ cRNA
mRNAs
*le*RNA
NP
Rluc
MG transfect.
EBOV infect.
MG transfect.
EBOV infect.
EBOV infect.
MG transfect.
M
bp
500
400
300
200
100

C

## Calculation of EBOV mRNA: *leader*RNA ratio

**Calculation of EBOV *leader*RNA + cRNA**

I) Mean $C_T$ value of EBOV-infected cells (3 biological replicates)

EBOV-infected cells:
Mean $C_{T\ leaderRNA+\ cRNA}$ = 23.802

**II)** Linear equation from standard curve **73-mer**: y = -1.571 ln(x) + 19.899

Solved for x: $x = e^{\left(\frac{y-19.899}{-1.571}\right)}$

**III)** Insert $C_T$ value for y into the equation

$$x = e^{\left(\frac{23.802-19.899}{-1.571}\right)}$$

$$x = 0.083\ amol$$

**Calculation of MG cRNA**

I) Mean $C_T$ value of EBOV-infected cells (3 biological replicates)

EBOV-infected cells:
Mean $C_{T\ leaderRNA+\ cRNA}$ = 24.237

II) Linear equation from standard curve 157-mer: y = -1.303 ln(x) + 18.59

Solved for x: $x = e^{\left(\frac{y-18.59}{-1.303}\right)}$

**III)** Insert $C_T$ value into the equation as y

$$x = e^{\left(\frac{24.237-18.59}{-1.303}\right)}$$

$$x = 0.0131\ amol$$

Calculation of cRNA

I) 0.083 amol – 0.0131amol= **0.0699 amol** → leader RNA in EBOV infected cells

## Calculation of mRNA from EBOV infected cells

**Calculation of EBOV mRNA**

I) Mean $C_T$ value from EBOV infected cells (3 biological replicates)

EBOV infected cells:
Mean $C_{T\ mRNA}$ = 21.87

**II)** Linear equation from standard curve 170mer: y= -1.479ln(x)+ 22.421

Resolved for x:

$$x = e^{\left(\frac{y-22.421}{-1.479}\right)}$$

**III)** Insert $C_T$ values into the equation as y

$$x = e^{\left(\frac{21.87-22.421}{-1.479}\right)}$$

$$x = 1.451\ amol$$ mRNA in EBOV infected cells

**Ratio of mRNA:leader RNA in EBOV infected cells**

$$\frac{1.451amol}{0.0699amol} = \mathbf{20.76}$$

# Calculation of MG mRNA : *leader*RNA ratio

**Calculation of MG leader RNA+ cRNA**

I) Mean $C_T$ value of minigenome (MG)-transfected cells (3 biological replicates)

MG-transfected cells:
Mean $C_{T\ \mathit{leader}\mathrm{RNA+\ cRNA}}$ = 21.282

**II)** Linear equation from standard curve **65-mer**: y = -1.657 ln(x) + 19.09

Solved for x: $x = e^{\left(\frac{y-19.09}{-1.657}\right)}$

**III)** Insert $C_T$ value for y into the equation

$$x = e^{\left(\frac{21.282-19.09}{-1.657}\right)}$$

$$x = 0.266\ amol$$

**Calculation of MG cRNA**

I) Mean $C_T$ value of Minigenome (MG) transfected cells (3 biological replicates)

MG transfected cells:
Mean $C_{T\ \mathit{leader}\mathrm{RNA+\ cRNA}}$ = 21.467

II) Linear equation from standard curve 157-mer: y = -1.303 ln(x) + 18.59

Solved for x: $x = e^{\left(\frac{y-18.59}{-1.303}\right)}$

**III)** Insert $C_T$ value for y into the equation

$$x = e^{\left(\frac{21.467-18.59}{-1.303}\right)}$$

$$x = 0.1099\ amol$$

**Calculation of cRNA: (*leader*RNA+ cRNA) - cRNA = *leader*RNA)**

I) 0.266 amol – 0.1099 amol = **0.1561 amol** → *leader*RNA in MG-transfected cells

## Calculation of MG mRNA

II) Mean $C_T$ value from minigenome (MG)-transfected cells (3 biological replicates)

MG transfected cells:
Mean $C_{T\ mRNA}$ = 17.572

**II)** Linear equation from standard curve 181-mer: y = -1.459 ln(x) + 22.536

Solved for x: $$x = e^{\left(\frac{y-22.536}{-1.459}\right)}$$

**III)** Insert $C_T$ value for y into the equation

$$x = e^{\left(\frac{17.572-22.536}{-1.459}\right)}$$

$$x = 30.034\ amol$$ mRNA in MG-transfected cells

**Ratio of mRNA : *leader*RNA in MG-transfected cells**

$$\frac{30.034\ amol}{0.1561\ amol} = \mathbf{192.4}$$

**Fig. S4. qRT-PCR using standard curves. (A)** qRT-PCR standard curves for the individual standard RNAs. The molar amount of the respective RNA fragment introduced into the qRT-PCR reaction is given on the x-axis in logarithmic scale. The equation of the linearized calibration curve and the coefficient of determination ($R^2$) are shown as inset. (**B**) RT-PCR products using total RNA from EBOV-infected or MG-transfected cells and the different primer pairs according to strategy 4 were analyzed on a 2% agarose gel; the PCR products obtained after 40 PCR cycles were stained with GelRed; M, 100 bp ladder (CytoGen GmbH, Sinn, Germany) used as size marker. (**C**) Example calculations of NP mRNA:*leader*RNA amounts in EBOV-infected cells based on the standard curve for the 73-meric *leader*RNA mimic, and Rluc mRNA:*leader*RNA amounts in MG-transfected cells based on the standard curve for the 65-meric *leader*RNA mimic.
