# Supplementary material for: Identification and characterization of short leader and trailer RNAs synthesized by the Ebola virus RNA polymerase
Source: PLoS Pathog. 2021 Oct 26;17(10):e1010002. doi: 10.1371/journal.ppat.1010002 (PMC8547711; doi:10.1371/journal.ppat.1010002)
Supplement: S5 Fig — RNA-Seq reads representing Renilla mRNA 5’-ends in poly(A) RNA fractions derived from cells transfected with the Δ5’ spacer MG, the NheI HP MG and the wt MG, either in the presence (A to C) or absence (D to F) of VP30. Panels A and D, identical to Fig 8D and 8E, are shown for comparison. Mean values (± SEM) are based on 2 to 4 biological replicates each. The dashed vertical line marks the canonical EBOV transcription start site (TSS). For more details, see legend to Fig 2B of the main text and S3 Table. (DOCX) [file ppat.1010002.s010.docx]

**
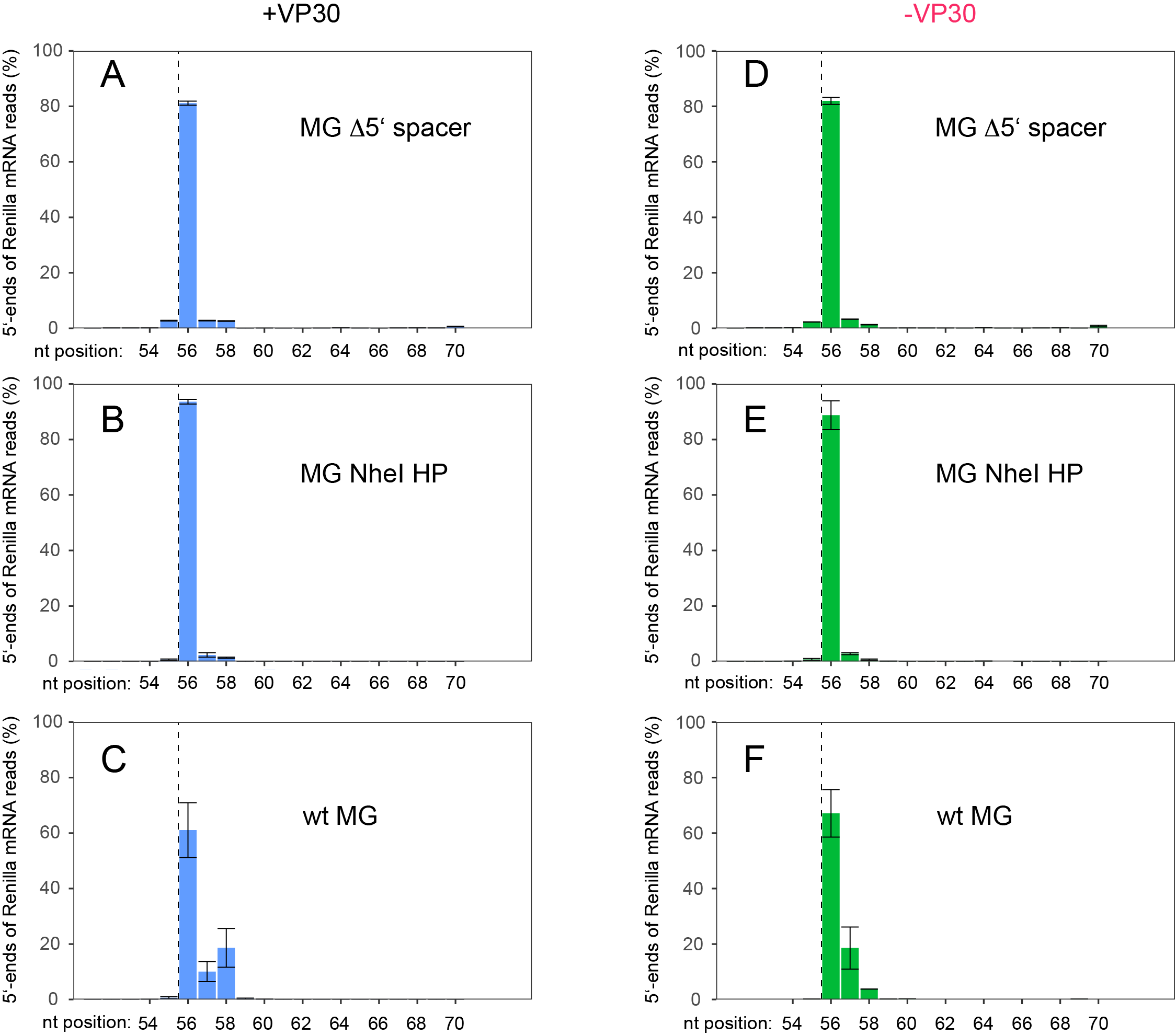
**

**S5 Fig.** RNA-Seq reads representing Renilla mRNA 5'-ends in poly(A) RNA fractions derived from cells transfected with the Δ5’ spacer MG, the NheI HP MG and the wt MG, either in the presence (**A to C**) or absence (**D to F**) of VP30. Panels A and D, identical to Fig 8D and 8E, are shown for comparison. Mean values (± SEM) are based on 2 to 4 biological replicates each. The dashed vertical line marks the canonical EBOV transcription start site (TSS). For more details, see legend to Fig 2B of the main text and S3 Table.
