# Supplementary material for: Identification and characterization of short leader and trailer RNAs synthesized by the Ebola virus RNA polymerase
Source: PLoS Pathog. 2021 Oct 26;17(10):e1010002. doi: 10.1371/journal.ppat.1010002 (PMC8547711; doi:10.1371/journal.ppat.1010002)
Supplement: S6 Fig — Comparative RNA-Seq analysis of leaderRNA lengths in RNA libraries derived from (A) EBOV-infected cells and (B-G) cells transfected with MGs (wt NP HP, NheI HP, Δ5’ spacer; illustrated in Fig 8A of the main manuscript) in the presence (B-D) or absence (E-G) of VP30. Mean values (± SEM) are based on 3 to 5 biological replicates each. Red bars or red part bars indicate reads with not more than 1 non-templated nt (= 1 mismatch) or 1 indel (insertion or deletion of 1 nt in the segemehl alignment) in the 3’-terminal 15 nt (canonical reads); blue bars or blue part bars indicate reads with at least 2 mismatches or indels in the 3’-terminal 15 nt. (error-prone reads). For more details, see legend to Fig 2A of the main text as well as S3 and S4 Tables. (DOCX) [file ppat.1010002.s011.docx]

**
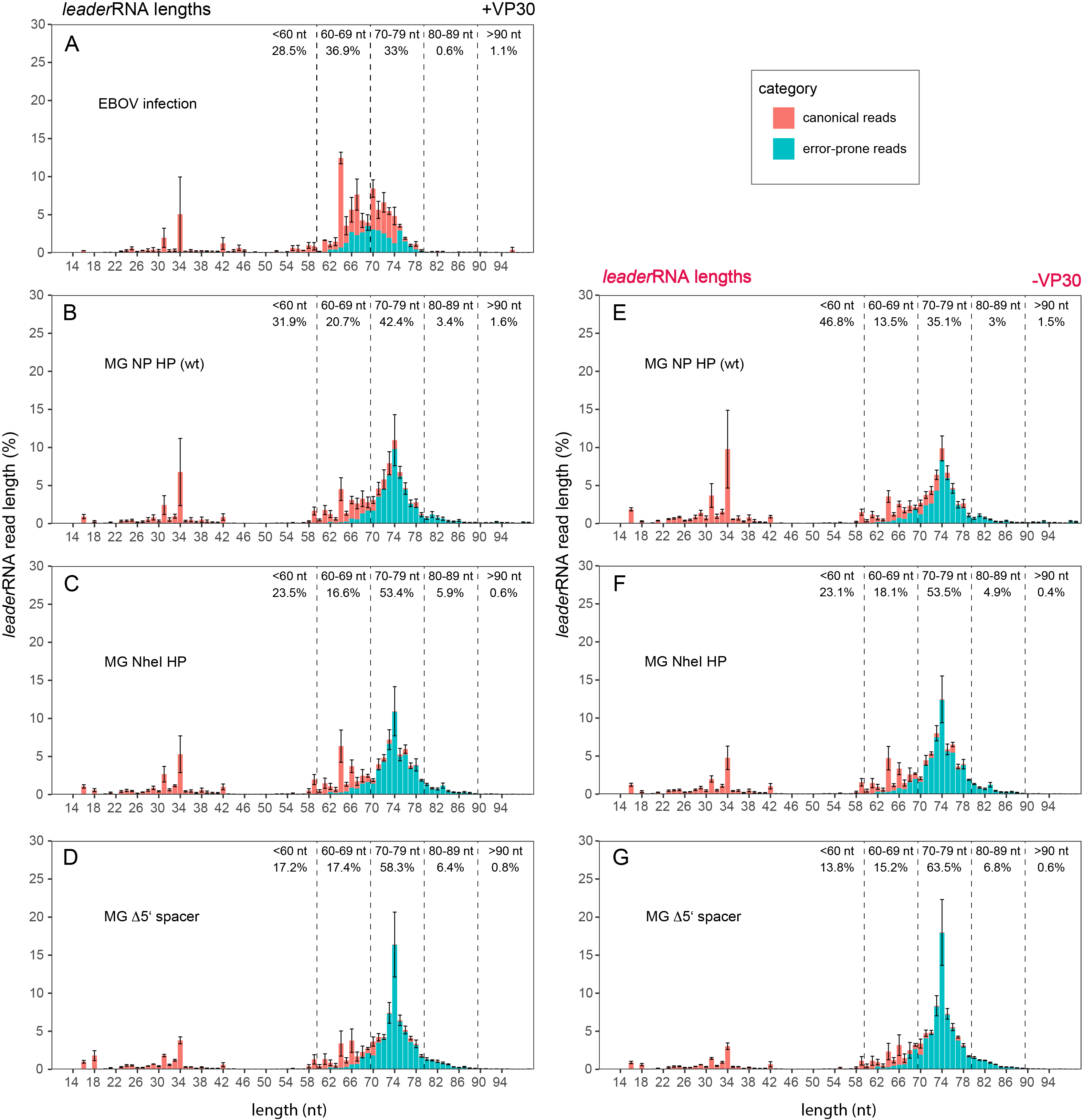
**

**S6 Fig.** Comparative RNA-Seq analysis of *leader*RNA lengths in RNA libraries derived from (**A**) EBOV-infected cells and (**B-G**) cells transfected with MGs (wt NP HP, NheI HP, Δ5’ spacer; illustrated in Fig 8A of the main manuscript) in the presence (**B-D**) or absence (**E-G**) of VP30. Mean values (± SEM) are based on 3 to 5 biological replicates each. Red bars or red part bars indicate reads with not more than 1 non-templated nt (= 1 mismatch) or 1 indel (insertion or deletion of 1 nt in the segemehl alignment) in the 3'-terminal 15 nt (canonical reads); blue bars or blue part bars indicate reads with at least 2 mismatches or indels in the 3'-terminal 15 nt. (error-prone reads). For more details, see legend to Fig 2A of the main text as well as S3 and S4 Tables.
