# Supplementary material for: Identification and characterization of short leader and trailer RNAs synthesized by the Ebola virus RNA polymerase
Source: PLoS Pathog. 2021 Oct 26;17(10):e1010002. doi: 10.1371/journal.ppat.1010002 (PMC8547711; doi:10.1371/journal.ppat.1010002)
Supplement: S7 Fig — (A, B) For the MGs (wt NP HP, NheI HP and Δ5’ spacer), read numbers in the presence (+) as well as absence (-) of VP30 are given; spike-in: a synthetic 5’-triphosphorylated 65-meric leaderRNA (sequence in S2 Table) was added to a small RNA preparation isolated from non-infected/non-transfected (mock-treated) HEK293 cells to control for authentic 5’-end representation in RNA-Seq libraries. The color code representing transcription starts at the first, second and third genome end position is indicated on the right. The number of biological replicates (n) is indicated above each column (for details, see S3 Table). (C) Comparison of trailerRNA read abundance in the small RNA-Seq libraries (< 200 nt) from EBOV-infected cells and from cells transfected with the different MG variants in the presence versus absence of VP30. Regarding the MG libraries, the difference between read numbers in the +VP30 versus –VP30 samples is neither significant if the three +VP30 and the three–VP30 MG libraries are each pooled (p = 0.211, Welch’s t test) nor if the individual MGs constructs are considered (p = 0.459 for wt NP HP, p = 0.451 for NheI HP, p = 0.767 for Δ5’ spacer; Welch’s t test). For number and details on biological replicates, see S3 Table. (DOCX) [file ppat.1010002.s012.docx]

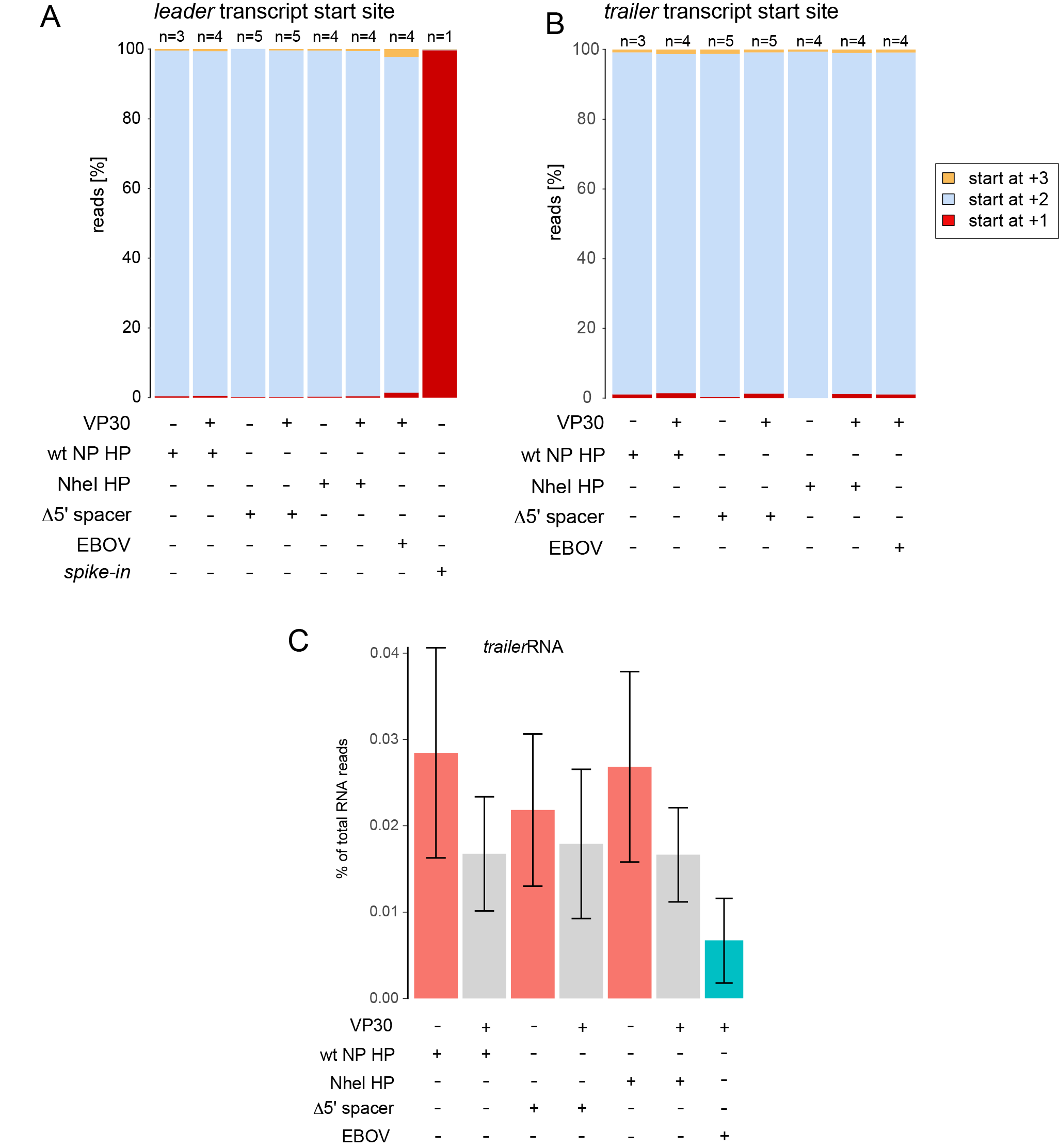


**S7 Fig.** *Leader* and *trailer* transcript 5'-ends determined by RNA-Seq for EBOV-infected HuH7 cells and MG-transfected HEK293 cells. (**A, B**) For the MGs (wt NP HP, NheI HP and Δ5’ spacer), read numbers in the presence (+) as well as absence (-) of VP30 are given; *spike-in*: a synthetic 5'-triphosphorylated 65-meric *leader*RNA (sequence in S2 Table) was added to a small RNA preparation isolated from non-infected/non-transfected (mock-treated) HEK293 cells to control for authentic 5'-end representation in RNA-Seq libraries. The color code representing transcription starts at the first, second and third genome end position is indicated on the right. The number of biological replicates (n) is indicated above each column (for details, see S3 Table). (**C**) Comparison of *trailer*RNA read abundance in the small RNA-Seq libraries (< 200 nt) from EBOV-infected cells and from cells transfected with the different MG variants in the presence versus absence of VP30. Regarding the MG libraries, the difference between read numbers in the +VP30 versus –VP30 samples is neither significant if the three +VP30 and the three –VP30 MG libraries are each pooled (p = 0.211, Welch’s *t* test) nor if the individual MGs constructs are considered (p = 0.459 for wt NP HP, p = 0.451 for NheI HP, p = 0.767 for Δ5’ spacer; Welch’s *t* test). For number and details on biological replicates, see S3 Table.
