# Supplementary material for: Identification and characterization of short leader and trailer RNAs synthesized by the Ebola virus RNA polymerase
Source: PLoS Pathog. 2021 Oct 26;17(10):e1010002. doi: 10.1371/journal.ppat.1010002 (PMC8547711; doi:10.1371/journal.ppat.1010002)
Supplement: S9 Fig — Following transfection of HEK293 cells with the RNAs specified at the bottom, the medium was removed and cells were gently washed with PBS. Subsequently, total cellular RNA was isolated and qRT-PCR was performed with the primer pair specific for the leaderRNA 65- and 73-mer (for details, see S1 Text, paragraph "qRT-PCR assay for innate immune induction"). (DOCX) [file ppat.1010002.s014.docx]

**
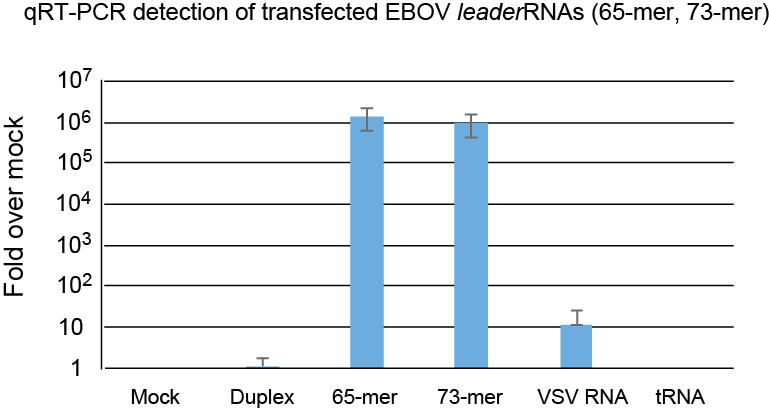
**

**S9 Fig.** Following transfection of HEK293 cells with the RNAs specified at the bottom, the medium was removed and cells were gently washed with PBS. Subsequently, total cellular RNA was isolated and qRT-PCR was performed with the primer pair specific for the *leader*RNA 65- and 73-mer (for details, see S1 text, paragraph "qRT-PCR assay for innate immune induction").
