# Supplementary material for: Identification and characterization of short leader and trailer RNAs synthesized by the Ebola virus RNA polymerase
Source: PLoS Pathog. 2021 Oct 26;17(10):e1010002. doi: 10.1371/journal.ppat.1010002 (PMC8547711; doi:10.1371/journal.ppat.1010002)
Supplement: S10 Fig — qRT-PCR reactions were performed with primer pairs specific for IFN-β mRNA (top, sky blue columns), CXCL10 mRNA (dark blue columns), VSV RNA (light green bars) and the EBOV leaderRNA 65-mer (pink columns). The slight reduction in IFN-β and CXCL10 mRNA levels at 500 and 750 ng leaderRNA can be attributed to slightly reduced cellular uptake of VSV RNA at excess amounts of the leaderRNA competitor in the transfection mix (see VSV graph, light green bars). For experimental details, see S1 Text, paragraph "qRT-PCR assay for innate immune induction". (DOCX) [file ppat.1010002.s015.docx]

**
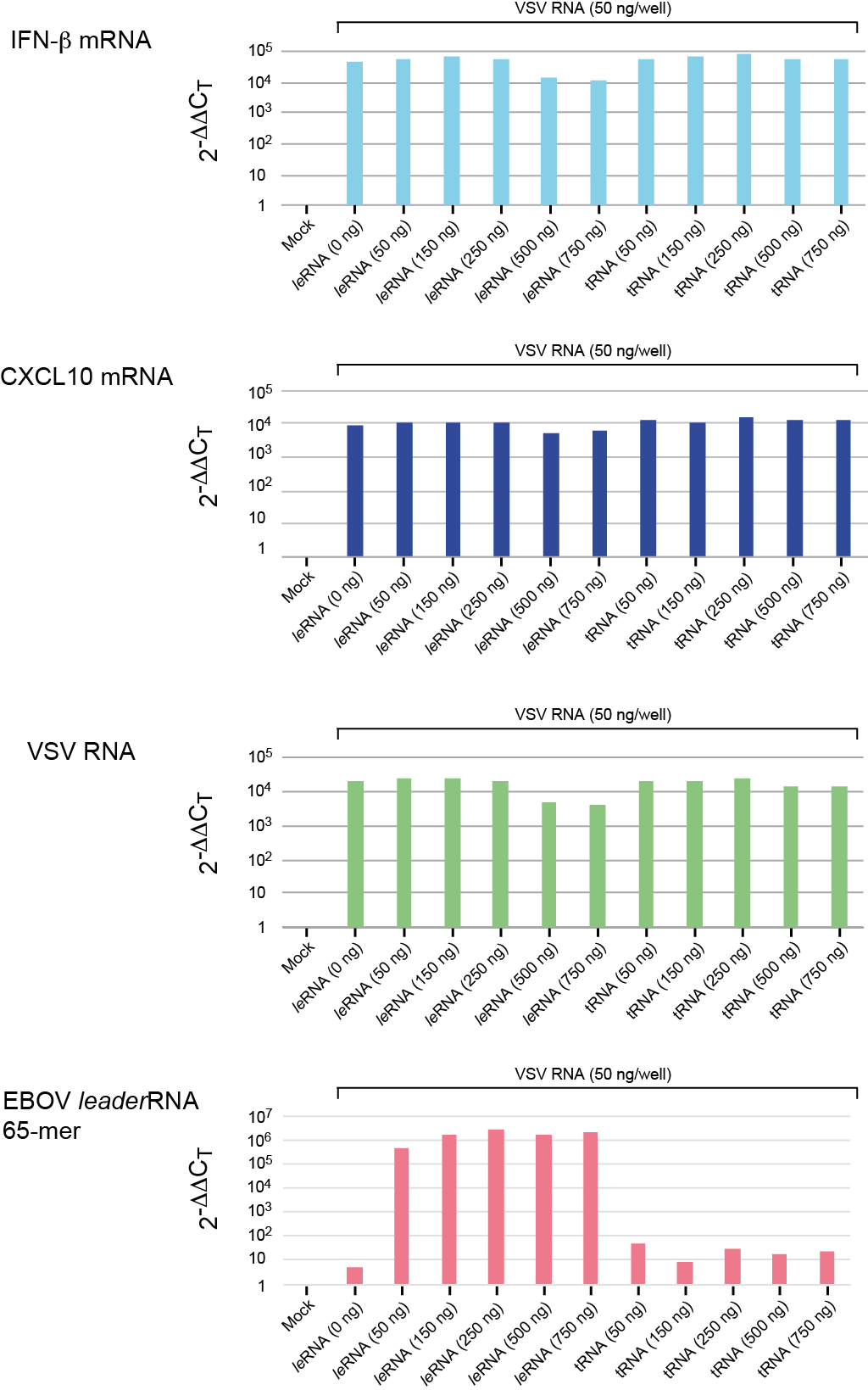
**

**S10 Fig.** Innate immunity response (inferred from increases in the levels of mRNAs coding for IFN-β and CXCL10) upon cotransfection of constant amounts of VSV RNA (50 ng/well) and increasing amounts of synthetic *leader*RNA (*le*RNA) 65-mer or bulk tRNA from yeast. qRT-PCR reactions were performed with primer pairs specific for IFN-β mRNA (top, sky blue columns), CXCL10 mRNA (dark blue columns), VSV RNA (light green bars) and the EBOV *leader*RNA 65-mer (pink columns). The slight reduction in IFN-β and CXCL10 mRNA levels at 500 and 750 ng *leader*RNA can be attributed to slightly reduced cellular uptake of VSV RNA at excess amounts of the *leader*RNA competitor in the transfection mix (see VSV graph, light green bars). For experimental details, see S1 text, paragraph "qRT-PCR assay for innate immune induction".
