# Supplementary material for: Identification and characterization of short leader and trailer RNAs synthesized by the Ebola virus RNA polymerase
Source: PLoS Pathog. 2021 Oct 26;17(10):e1010002. doi: 10.1371/journal.ppat.1010002 (PMC8547711; doi:10.1371/journal.ppat.1010002)
Supplement: S11 Fig — (DOCX) [file ppat.1010002.s016.docx]

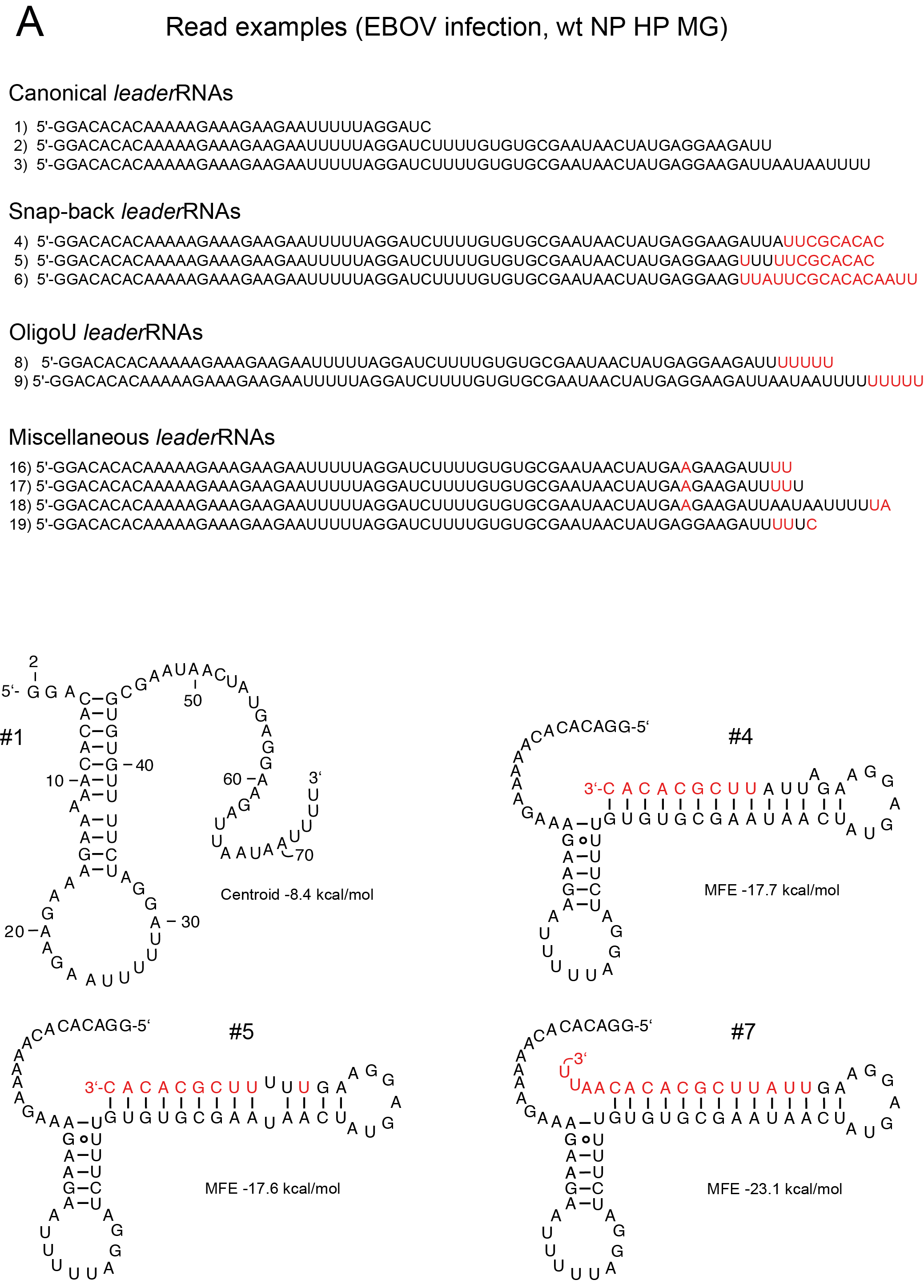


**
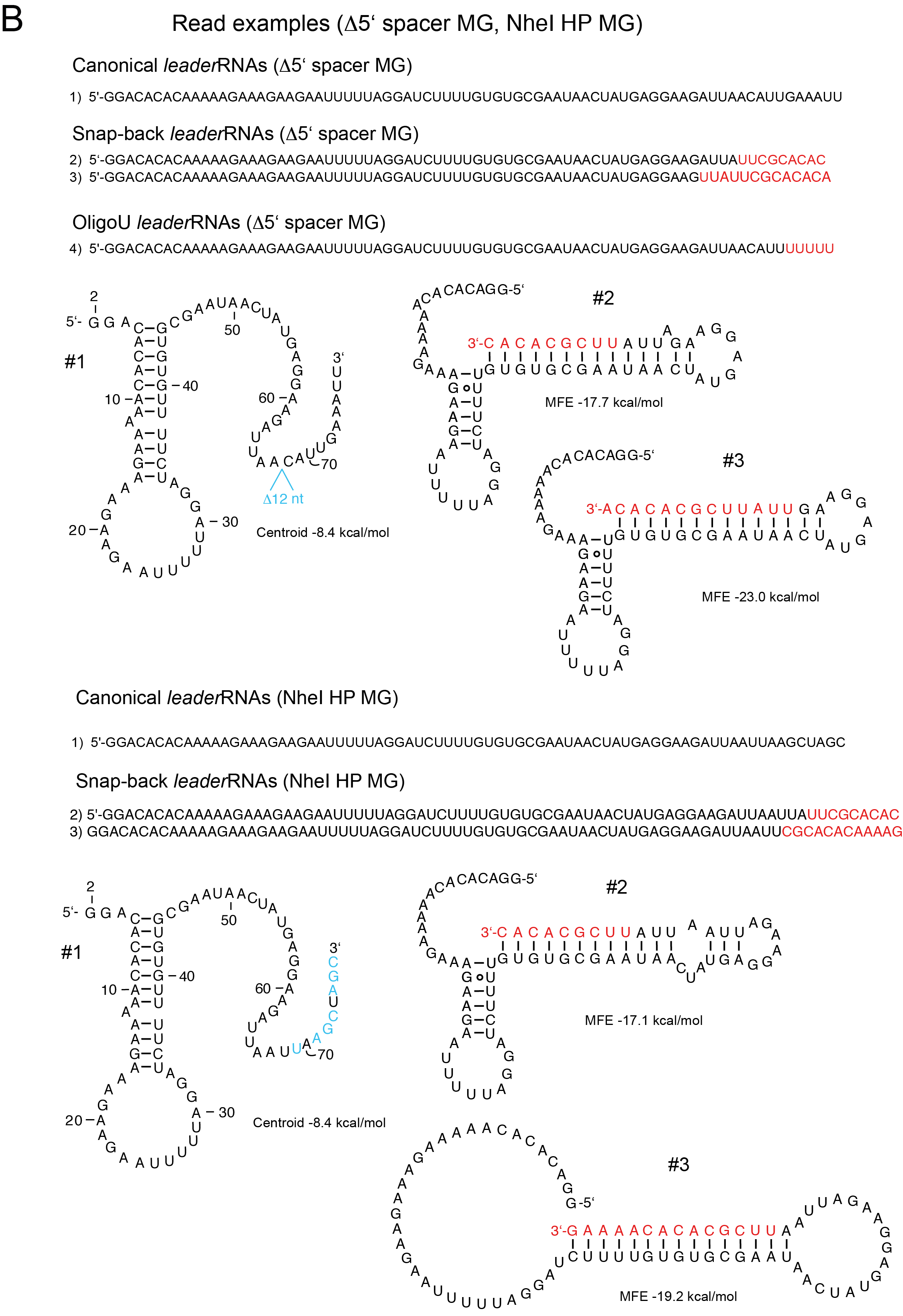
**

**S11 Fig.** Examples of categorized *leader*RNA reads and their predicted structures, (**A**) taken from libraries representing EBOV-infected cells and cells transfected with the wt NP HP MG, or (**B**) derived from cells transfected with the Δ5'-spacer or NheI HP mutant MGs. *Leader*RNA reads were categorized as follows: canonical: reads with not more than 1 non-templated nt (= 1 mismatch) or 1 indel (insertion or deletion of 1 nt in the segemehl alignments) in the 3'-terminal 15 nt (canonical reads); all other reads had at least 2 mismatches or indels in the 3'-terminal 15 nt and were further grouped as oligoU, snap-back and miscellaneous; oligoU: at least seven 3'-terminal U residues with up to 1 mismatch or indel; snap-back: the last 25 nt can form a hairpin (MFE structure, RNAfold) with a stability of at least -5 kcal/mol and the last 15 nt contain one of the following sequences: 5'-UUCGCAC, 5'-CGCACAC or 5'-CGCACACAA with up to 1 mismatch or indel; the snap-back reads are attributable to RdRp releasing the genomic template strand and utilizing instead the newly synthesized RNA transcript as template for the synthesis of several nucleotides. Miscellaneous: all other non-canonical reads that did not entriely adhere to the above criteria for oligoU and snap-back reads. Red nt: not encoded in the genomic RNA; the 12-nt deletion in the Δ5'-spacer MG and the mutations in NheI HP MG are marked in light blue.
